# Enhancing molecular property prediction of transformer models with dual graph representation

**DOI:** 10.1038/s41467-026-75005-9

**Published:** 2026-06-27

**Authors:** Shuyuan Zhang, Alexei A. Lapkin

**Affiliations:** 1 Department of Chemical Engineering and Biotechnology, University of Cambridge, Philippa Fawcett Drive, Cambridge, UK; 2 Innovation Centre in Digital Molecular Technologies, Yusuf Hamied Department of Chemistry, University of Cambridge, Lensfield Road, Cambridge, UK

**Keywords:** Cheminformatics, Method development, Computational models

## Abstract

Accurate prediction of molecular properties is central to advancing chemistry, materials science, and drug discovery. Machine learning on molecular graphs depends critically on representations that capture the topology and structure of molecules. Here we propose the dual graph transformer (DGT), a self-attention architecture that jointly models atom and bond graphs to achieve comprehensive molecular encodings. DGT fuses atom and bond features, graph topology and structure, and stereogeometric information within its self-attention module for an effective molecular representation. We benchmark DGT across a range of datasets for molecular property prediction, showing that it considerably outperforms the current state of the art. DGT demonstrates performance contributions from its dual graph representation, relative positional and structural encodings, and stereogeometric information incorporation while also offering interpretability at the molecular structural level. We envision DGT advancing molecular machine learning by improving both the prediction accuracy and interpretability of molecular properties.

## Abstract (web summary)

Molecular representation is critical for property prediction. Here authors introduce a dual graph transformer that fuses atom, bond, topology, structure, and stereogeometric features within self-attention to enhance molecular property prediction.

## Introduction

Molecular science sits as a cornerstone of many application domains, as it underpins our understanding and manipulation of matter at the fundamental level^1,2^. Within molecular sciences, accurate prediction of molecular properties plays a critical role in exploring chemical spaces across versatile domains where wet-lab experiments and first-principles calculations are often costly and time-consuming^3,4^. Deep learning methods, which bypass the need for hand-crafted feature design through automatic feature extraction, offer a promising avenue toward high-throughput screening of molecules for desired properties, such as chemical reactivity^5^, solubility parameters^6^, HOMO–LUMO gaps^7^, and binding affinities to proteins^8^. Beyond accelerating molecular screening, these methods also provide new insights into the underlying factors that drive molecular behaviours of great interest.

At the core of molecular property prediction lies the approach for representing molecules, which is closely connected to model architectures and critically influences learning performance^9^. A wide spectrum of molecular representations has been explored: Fingerprints and descriptors encode molecules as fixed-length vectors by summarising substructures or cheminformatics-derived features^10^; string-based representations, such as the simplified molecular-input line-entry system, tokenise molecules as sequence data, which are readily handled by recurrent neural networks and large language models^11^; and graph-based representations capture atom and bond features alongside connectivity patterns, providing a more faithful depiction of molecular structures^12^. Such molecular graphs support graph neural networks (GNNs) to directly learn representations from molecular structures instead of pre-computed features, attracting growing interest. However, the message-passing mechanism underlying GNNs comes with challenges of over-smoothing and limited *k*-hop neighbourhoods, constraining the expressivity and receptive field of node features^13,14^. Meanwhile, the attention mechanism achieves substantial success in modelling sequence data, motivating its application to molecular representation learning^15^. The graph transformer, which harnesses the self-attention mechanism to enable global dynamic node–node interactions regardless of their graph distances, is surging recently^16^.

Employing the self-attention mechanism on molecules requires the comprehensive encoding of molecular information, including atom and bond features, graph topology and structure, and stereogeometric information, if available, in the query-key-value operation^17,18^. Graph-specific encodings—including relative positional encoding (RPE)^19^ and ring structural encoding (RSE)^20^—can be incorporated into the graph transformer layer, along with edge features, to capture complex dependencies within molecules^21^. In some graph transformer implementations, edge features are also aggregated as characterisation of local neighbourhoods for updating node features before the attention calculation^22^. Despite that molecules are often intuitively depicted as topological graphs with atoms as nodes, reinterpreting bonds as nodes in molecular graphs can provide an alternative perspective to enrich molecular representation in the attention mechanism and thereby promote property prediction^23^.

To this end, we propose the dual graph transformer (DGT), a novel deep learning architecture that leverages the self-attention mechanism for enhancing molecular property prediction by integrating the atom graph with its bond counterpart. Atom and bond features are mutually fused into the attention score matrices of their counterparts. RPE and RSE are incorporated into the attention matrices of both atom and bond graphs. DGT also enables encoding bond lengths, atom–atom distances, and bond–bond angles into the self-attention mechanism for the optional adoption of three-dimensional (3D) information of different fidelities. Chirality and *E/Z* isomerism information can also be integrated into DGT for stereoselective tasks. DGT is benchmarked across 10 datasets and 58 tasks for molecular property prediction and considerably outperforms the current state of the art. Further performance investigation validates contributions of dual graph representation, graph-specific encodings, 3D and stereochemical structure incorporation, and model pretraining to molecular property prediction. We also demonstrate that the attention-based interpretation can reveal crucial substructures for molecular properties on the dual graph representation. DGT presents as a powerful deep learning architecture for effective molecular representation learning to advance prediction and understanding of molecular properties.

## Results

### Overview of DGT

**Fig. 1:**
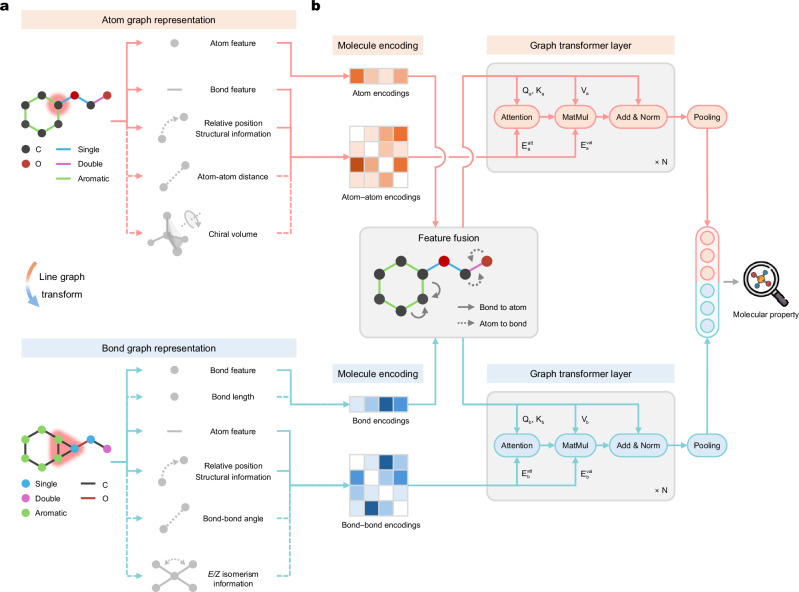
An illustration of the Dual Graph Transformer (DGT). **a** Atom and bond graph representations of molecules. Positions of atoms and bonds are exchanged in the dual graph representation, accommodating atom and bond features, relative position, and structural information. For instance, the highlighted *ipso*-carbon in the atom graph corresponds to the three highlighted edges in the corresponding bond graph. Bond length, atom–atom distance, and bond–bond angle can also be integrated into DGT as geometric information when available. DGT is able to incorporate chiral volume and *E/Z* isomerism information to enhance performance on stereoselective tasks. **b** Molecule encodings, feature fusion, and graph transformer structure. DGT encodes molecules into atom and bond vectors, atom–atom matrices, and bond–bond matrices. Atom and bond encodings are fused before the query-key-value operation by stacked graph transformer layers, followed by the pooling layer for predicting molecular properties. In the atom graph, ${{{\rm{Q}}}}_{{{\rm{a}}}}$, ${{{\rm{K}}}}_{{{\rm{a}}}}$, and ${{{\rm{V}}}}_{{{\rm{a}}}}$ denote the query, key, and value in the self-attention mechanism, while ${{{\rm{E}}}}_{{{\rm{a}}}}^{{{\rm{att}}}}$ and ${{{\rm{E}}}}_{{{\rm{a}}}}^{{{\rm{val}}}}$ represent edge features incorporated into the attention and value computations, respectively. ${{{\rm{Q}}}}_{{{\rm{b}}}}$, ${{{\rm{K}}}}_{{{\rm{b}}}}$, ${{{\rm{V}}}}_{{{\rm{b}}}}$, ${{{\rm{E}}}}_{{{\rm{b}}}}^{{{\rm{att}}}}$ and ${{{\rm{E}}}}_{{{\rm{b}}}}^{{{\rm{val}}}}$ are their counterparts in the bond graph.

The proposed DGT is illustrated in Fig. 1, encompassing stages of graph representation, molecule encoding, stacked graph transformer layers, and a final readout module for molecular property prediction. The bond graph representation is derived by reinterpreting the intuitive atom graph with the line graph transform, which exchanges the roles of atoms and bonds in the topological structure. This provides an alternative perspective to enrich molecular features, thereby enhancing the property prediction performance of deep learning approaches. As the exemplified phenyl formate in Fig. 1a, carbon and oxygen atoms are regarded as edges in the bond graph representation, depicting connections of single, double, and aromatic bonds. The highlighted *ipso*-carbon in the atom graph corresponds to the three highlighted edges, sharing the same feature vector, in the bond graph.

Such a dual graph representation realises the encoding of comprehensive molecular information—including atom and bond features, graph topology, specific structures, and 3D information if available—into deep learning architectures for molecular learning tasks. In detail, atom and bond features are represented as node vectors in their respective graphs and as edge vectors in their mutual graphs. Shortest path distance encoding (SPDE) and random walk positional encoding (RWPE) are employed as the RPE of nodes; RSE is added to deliver ring information, given its importance for many molecular properties (Figure S1). Together, these graph-specific encodings are combined with edge features to form node–node matrices as pairwise features for both atom and bond graphs. Additionally, this dual graph representation accommodates diverse stereogeometric information: Bond lengths are embedded within bond features, while atom–atom distances and bond–bond angles are mapped onto the pairwise feature matrices of atom and bond graphs, respectively. The chiral volume is encoded as a stereochemical descriptor in the atom–atom encodings; relative orientation of vicinal bonds in *E/Z* isomers is integrated into the bond–bond encodings. To smoothen the continuous geometric information of bond–bond angles, the normalised spherical harmonics are employed to provide an orthonormal representation of angular dependencies (Figure S2).

The feature fusion module positioned upstream stacked graph transformer layers employs a bidirectional aggregation strategy to synthesise an integrated representation: bond embeddings are augmented by concatenating their feature vectors with the mean representation of their incident atoms. Conversely, atomic embeddings are enriched by incorporating the average of their adjacent edge representations (Fig. 1b). The graph transformer layer extends the conventional query-key-value operation by merging the pairwise feature matrices from atom and bond graphs into their respective attention score matrices and passing messages, allowing DGT to learn both local and global dependencies for atoms and bonds. In addition, the pairwise feature matrices are also utilised to update node vectors within the self-attention calculation, providing an effective means to enhance node messages, as demonstrated in recent studies^24,25^. Batch normalisation is applied in each graph transformer layer to stabilise training. Details of molecule encoding, feature fusion, and graph transformer layer are provided in the “Methods” section and Figure S3. After stacked graph transformer layers, the global average pooling is employed as a readout function, followed by the concatenation operation of outputs of both graph representations and fully connected layers for molecular property prediction.

### Molecular property prediction benchmark

We conducted a comprehensive benchmark study of DGT on molecular property prediction across 10 datasets and 58 tasks, spanning four domains: physiology, biophysics, physical chemistry, and quantum mechanics (Table S1). DGT was evaluated against nine state-of-the-art machine learning models to demonstrate its superior performance in learning on molecules, with configuration parameters listed in Table S2. As a classical baseline, the random forest (RF) model was built using ECFP4 fingerprints as input features^26^. Attentive FP^15^ and D-MPNN^27^ served as representative GNN baselines. In addition, pretrained GNN^28^, GraphMVP^29^, MolCLR^30^, and MoleBERT^31^ were included as GNN models leveraging large-scale molecular corpora for pretraining followed by task-specific fine-tuning. GROVER was implemented as a graph transformer framework capturing rich structural and semantic information within molecular graphs^32^. UniMol is represented as a transformer-based 3D geometric deep learning model for molecular property prediction^33^.

**Fig. 2:**
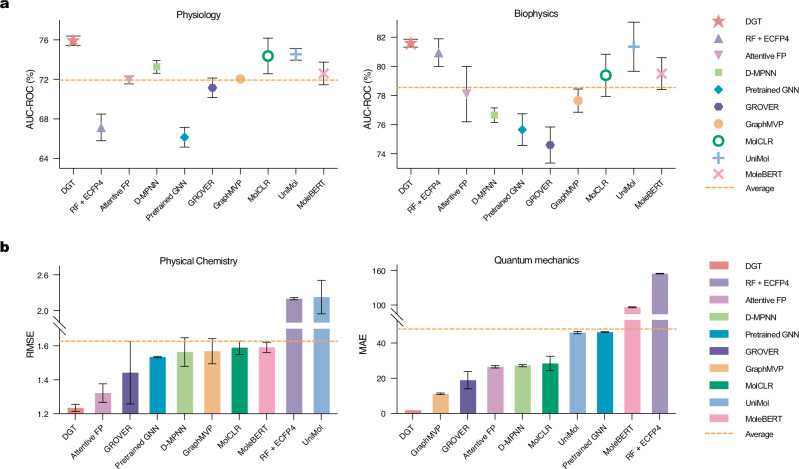
Benchmarking DGT against baselines across four domains. **a** Performance comparison of DGT against baselines for predicting categorical molecular properties in physiology and biophysics domains. **b** Performance comparison of DGT against baselines for predicting numerical molecular properties in physical chemistry and quantum mechanics domains. All error bars represent the standard deviation derived from four runs. Source data are provided as a Source Data file.

DGT outperformed all baseline models on classification tasks in the physiology and biophysics domains, as illustrated in Fig. 2a. Within the physiology domain, DGT displayed a substantial uplift in the area under the receiver operating characteristic curve (AUC-ROC), delivering a 1.36% improvement over the second-best model’s 74.55% and vastly exceeding the average AUC-ROC of 71.92%. Concurrently, in the biophysics domain, DGT likewise attained the top performance, registering an AUC-ROC of 81.10% and performing competitively with the pretrained transformer-based UniMol model. Model performance breakdowns for subtasks in the physiology and biophysics domains are detailed in Figures S4 and S5. These collective results affirm DGT’s broad applicability and high accuracy in predicting molecular properties relevant to drug discovery, including pharmacokinetics, toxicity, bioactivity, and potential adverse effects.

In the physical chemistry and quantum mechanics domains, DGT also observed significant advantages over all baselines (Fig. 2b). For physical chemistry tasks, DGT suggested a low root mean square error (RMSE) of 1.187, representing a notable 10.21% performance advancement over the next best model. In comparison, GNN baselines with pretraining reached a minimum RMSE of 1.443, and the RMSE values of RF and UniMol were even larger than 2.2. In the quantum mechanics domain, DGT outstood all other models, achieving a markedly low mean absolute error (MAE) of 1.840, which is an order of magnitude lower than that of the second-best model, GraphMVP. Figures S6 and S7 display model performance breakdowns for subtasks of physical chemistry and quantum mechanics domains, respectively. These results unveil the exceptional ability of DGT to precisely predict numerical physicochemical and quantum mechanical properties for molecules.

DGT surpasses other models by providing a rational approach to molecular representation and intimately coupling molecule encodings with the self-attention mechanism in the context of graph-structured molecular data. Unlike natural languages, where attention was initially devised to model sequential word dependencies, molecular graphs pose unique challenges arising from their complex topological structures and the absence of an inherent sequential order. Consequently, executing the attention mechanism in molecular machine learning necessitates specialised adaptations to capture both local and long-range interactions among atoms and bonds. The graph transformer layer addresses this by enabling nodes to aggregate information directly from the entire graph, rather than being restricted to their *k*-hop neighbourhoods, while embedding graph-specific encodings into the attention score matrix, including topological and structural information. Furthermore, the DGT’s outstanding performance is underscored by its ability to outperform baselines with pretraining, such as GraphMVP, despite their extensive molecular data exposure.

### Performance investigation of DGT

We investigated the performance of DGT through comparative analyses focused on the prediction of highest occupied and lowest unoccupied molecular orbital energies (*ε*_HOMO_ and *ε*_LUMO_), two pivotal electronic properties governing reactivity and stability, using the QM9 dataset^26^. To evaluate the specific contributions of stereochemical encodings, ablation analysis was also conducted on the Tox21 Aromatase and Oestrogen Receptor (ER) classification tasks. These targets are selected because their biological activity is highly sensitive to the steroidal scaffold, where both *R/S* chirality and *E/Z* isomerism have a prominent impact^34–36^.

**Fig. 3:**
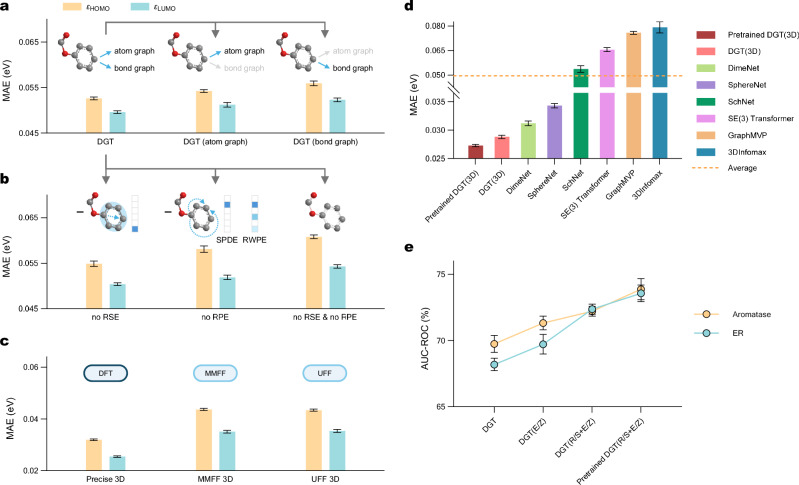
Performance investigation of DGT and stereogeometric information incorporation. The efficacy of incorporating 3D information was evaluated through HOMO and LUMO energy predictions on the QM9 dataset, while the impact of R/S chirality and E/Z isomerism was validated using the Tox21 Aromatase and Oestrogen Receptor (ER) classification tasks. **a** Ablation study evaluating the dual graph representation. **b** Ablation study of graph-specific encodings. **c** Effect of incorporating 3D information at different fidelity levels on DGT’s predictive performance. **d** Comparative assessment of the 3D information-augmented DGT, denoted as DGT(3D), against state-of-the-art models for 3D molecular learning using the average prediction MAE of HOMO and LUMO energies. The pretraining of DGT(3D) is conducted on molecules from the PCQM4Mv2 dataset^43^ targeting the HOMO–LUMO energy gap. **e** Ablation study evaluating the contribution of *R/S* chirality and *E/Z* isomerism to DGT performance. A reported PubChem3D subset for enantiomer recognition is adopted for pretraining^61^. All error bars represent the standard deviation derived from four runs. Source data are provided as a Source Data file. (RPE relative positional encoding, RSE ring structural encoding, SPDE shortest path distance encoding, RWPE random walk positional encoding, MMFF Merck molecular force field, UFF universal force field).

As shown in Fig. 3a, both atom and bond graph representations demonstrably contributed to predicting *ε*_HOMO_ and *ε*_LUMO_. Removing the bond graph representation from DGT led to an average MAE increment of 3.1%, while omitting the atom graph representation resulted in a more substantial 5.9% increment. Similarly, RPE and RSE, which were included in the self-attention calculation as structural and topological information, proved critical (Fig. 3b). Their exclusion produced average MAE increases of 3.0% and 7.5%, respectively. Collectively, DGT revealed an average performance degradation of 12.5% when both RPE and RSE were omitted. These findings substantiate the rationale of the dual graph representation to integrate comprehensive molecular information, underscoring the synergistic effects of DGT components in enhancing the graph transformer’s predictive power for molecular properties.

Fig. 3c elucidates the predictive capabilities of DGT when augmented with 3D structural information, including bond length, atom–atom distance and bond–bond angle, of varying fidelities. We investigated DGT’s performance using precise spatial coordinates derived from Density Functional Theory (DFT) calculations for constructing the QM9 dataset, alongside approximate geometries generated using the Merck Molecular Force Field (MMFF) and the Universal Force Field (UFF) implemented in RDKit^37^. The results demonstrated DGT’s ability to utilise 3D information across different fidelities for improving *ε*_HOMO_ and *ε*_LUMO_ predictions. Specifically, the precise DFT-derived 3D information yielded a significant average MAE reduction of 43.9%. Even the coarser MMFF- and UFF-generated geometries, while less effective, still contributed to a notable MAE reduction of about 23.1%.

Fig. 3d compares DGT(3D) against some established state-of-the-art models designed for 3D molecular learning. The benchmark models included SchNet^38^, DimeNet^39^, SphereNet^40^, SE(3) Transformer^41^, GraphMVP^29^, and 3DInfomax^42^. SchNet, DimeNet, and SphereNet represented 3D GNNs that use rotationally invariant features to predict scalar molecular properties. The SE(3) Transformer was included as a typical equivariant GNN that is robust to 3D roto-translations of molecules. GraphMVP and 3DInfomax were models adopting contrastive learning to enrich 2D graph structure embeddings with 3D conformational information. DGT(3D) outperformed all other evaluated models, firmly establishing it as a highly effective 3D machine learning architecture for molecular property prediction (detailed results in Figure S8). When pretrained on 10 K random molecules sampled from the PCQM4Mv2 dataset^43^, DGT(3D) attained prediction MAEs of 0.0306 eV for *ε*_HOMO_ and 0.0240 eV for *ε*_LUMO_, though no significant correlation was observed between pretraining dataset size and downstream prediction accuracy (Figure S9 and Table S3).

The efficacy of integrating chiral volume and *E/Z* isomerism with DGT was validated on the Tox21 Aromatase and ER tasks, both of which exhibit high stereoselectivity due to their steroidal-binding motifs. As hypothesised, the inclusion of stereochemical descriptors consistently improved the classification performance (Fig. 3e). Incorporating *E/Z* isomerism information into the bond–bond encodings (DGT(E/Z)) yielded an approximate 1.5% increase in AUC-ROC across both tasks. This performance was further enhanced in DGT(R/S + E/Z), which incorporates chiral volume into the atom–atom encodings to resolve enantiomeric ambiguity. Based on this architecture, a pretraining strategy was leveraged using a PubChem3D subset, where DGT outperformed other stereochemical models in enantiomer recognition (Figure S10). This specific pretraining stage facilitated an additional gain of about 1.4% in AUC-ROC for both Tox21 tasks.

These results highlight the potential of leveraging stereogeometric information in the dual graph representation to enhance the self-attention mechanism in molecular machine learning. Furthermore, model parameter amount and floating-point operations during inference are summarised in Table S4, demonstrating that DGT’s performance stems from the dual graph representation rather than parameter scaling.

### Attention-based interpretation

**Fig. 4:**
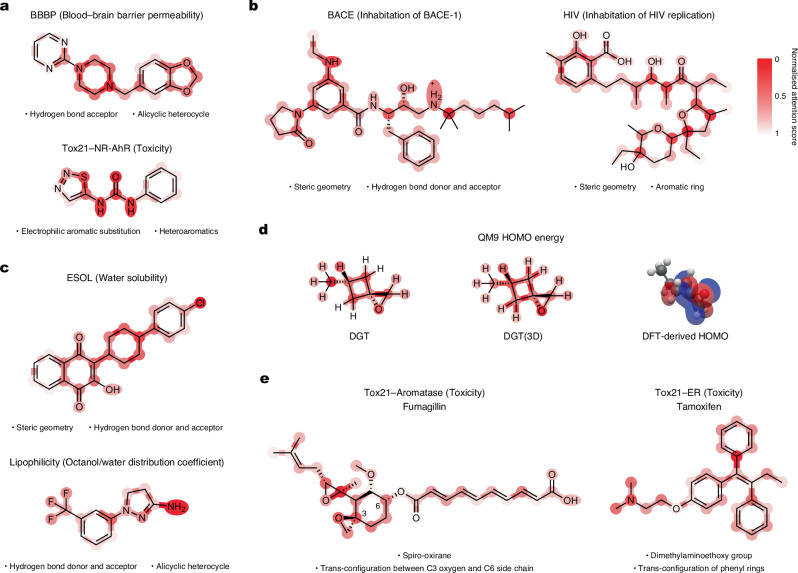
Attention-based interpretation analysis results. **a**–**d** Attention-based interpretation is conducted on molecular property prediction tasks across all four scientific domains: physiology (**a**), biophysics (**b**), physical chemistry (**c**), and quantum mechanics (**d**). Attention scores calculation concurrently considers the attention score matrices of atoms and bonds, as well as the absolute gradients of the prediction with respect to them. Darker colours denote a higher level of attention score assigned to the atom or bond. Attention scores calculated using DGT(3D) are provided for the QM9 HOMO energy prediction example, along with the DFT-derived HOMO at the B3LYP/6-31 G(2df,p) level of theory. **e** Attention-based stereochemical interpretability analysis using DGT with chirality and *E/Z* isomerism encodings incorporated. Fumagillin and tamoxifen are presented as representative stereoisomeric inhibitors for the Tox21 Aromatase and ER assays, respectively.

The global attention score matrices of DGT reveal the intricate interplay of molecular features for molecular property prediction, unravelling insights into the model’s decision-making and identifying key contributing molecular substructures. To jointly account for the model’s focus and the sensitivity of its prediction, the multi-head attention scores are multiplied with the absolute gradients of the prediction with respect to each element^44,45^. The resulting dot products qualify the significance values of pairwise relationships for the specific task, which are subsequently aggregated to evaluate the contribution of atoms and bonds (see Supplementary Information for details). Fig. 4 presents molecular examples illustrating this attention-based interpretation across benchmark datasets.

Within the physiology domain, hydrogen bond acceptor atoms and alicyclic heterocycles are highlighted in the example molecule from the BBBP dataset (Fig. 4a). This observation aligns with established principles governing blood–brain barrier penetration, which generally favour molecules with high lipophilicity and a limited number of hydrogen bond donors and acceptors^46^. For the Tox21–NR-AhR toxicity prediction task, ligands of aryl hydrocarbon receptor, which is a transcription factor, are predominantly electron-deficient aromatic systems, closely correlated with the attentive region identified in the illustrated molecule^47^.

The BACE and HIV datasets are representative for examining the attention-based interpretation in the biophysics domain. In both cases, attention weights are predominantly focused on atoms with steric geometries, with secondary attention distributed across hydrogen-bonding sites and aromatic rings for predicting the inhibition of human β-secretase 1^48^ and HIV replication^49^, respectively (Fig. 4b). These structural factors are widely recognised as determinants of inhibitor binding at target active sites.

Fig. 4c showcases the calculated attention scores for water solubility and lipophilicity predictions, two properties that are generally inversely correlated^50^. Hydrogen bond donors and acceptors, polar groups, and hydrophobic surface area are vital factors for them, to which the highlighted molecular substructures in the shown molecules conform.

In the QM9 HOMO energy prediction task, 3D information helps disentangle key molecular sections when incorporated into the DGT model^51^. Compared to DGT, DGT(3D) performs better to distribute greater attention to the epoxide and cyclobutane rings in the illustrated molecule, which is corroborated by the DFT-derived HOMO (Fig. 4d).

Fumagillin and tamoxifen are incorporated in Fig. 4e as representative stereoisomeric inhibitors for the Tox21 Aromatase and ER classification assays, respectively. For fumagillin, the attention is primarily localised on the spiro-oxirane moiety and the chiral centres of the cyclohexane ring. This attention distribution aligns with the established inhibitory mechanism^52^: the highly strained and electrophilic spiro-oxirane acts as a reactive site for irreversible covalent bonding with a histidine residue in the enzyme pocket. Meanwhile, the 1,4-*trans*-configuration between C3 oxygen and C6 side chain is structurally critical to ensure the precise spatial orientation of the spiro-oxirane toward the active site residues. In the case of tamoxifen, the normalised attention scores highlight the dimethylaminoethoxy side chain and the triphenylethylene core. Upon binding to the ER, the triphenylethylene core serves as a structural anchor to occupy the hydrophobic binding pocket, while the bulky, basic dimethylaminoethoxy chain protrudes out of the binding pocket, preventing Helix 12 from folding over the pocket^53^. Notably, the *E*-isomer (where the phenyl rings are *cis*-positioned) exhibits significantly lower affinity for the ER^35^.

These findings underscore the potential of DGT to offer valuable insights for molecular properties at the molecular structural level.

## Discussion

In this work, we present DGT as a graph transformer model which harnesses the dual graph representation for molecular property prediction. By enriching information in the molecular representation, the potential of the self-attention mechanism within the graph transformer model is unlocked for learning on molecules. DGT achieves state-of-the-art performance across 58 tasks from 10 datasets across four domains of the MoleculeNet benchmark. Further investigation reveals the effectiveness of the dual graph representation, graph-specific encodings, and stereogeometric information incorporation for enhancing the prediction accuracy, as well as the attention mechanism’s ability to identify molecular substructures critical to the target molecular property.

Deep learning has emerged as a powerful approach to expedite the exploration of molecules with desired properties across versatile domains. The graph transformer, which enables global dynamic node–node interactions regardless of their graph distances, is attracting wide interest in the field. Our work underscores the rationale to bridge the gap between molecular representation and the powerful attention mechanism for molecular property prediction. However, despite the fact that DGT demonstrates massive benefits from utilising stereogeometric information in molecular machine learning, the potential to generate high-fidelity molecular spatial structure is yet to be explored. Such a structure generation model can be added to the current workflow of DGT as an end-to-end approach. The node–node embeddings within DGT can be further extended to incorporate structural information derived from specific molecular motifs^54^ and topological descriptors derived from the Laplacian matrix^55^, graph kernels^56^, or Green’s functions^57^. In addition, some recent publications report improved pretraining on molecules using node-level context prediction over the graph-level attribute prediction^28^. DGT also holds the potential to promote more advanced domains such as conformation generation, molecular generative modelling, and chemical reaction prediction. It is also notable that the integration of atom–atom and bond–bond encodings introduces a computational complexity that scales quadratically with the molecular size. Model parallelism and the adoption of hierarchical or sparse attention mechanisms^58^ can be considered to extend the DGT’s utility on large molecules. Furthermore, despite the performance on MoleculeNet, we recognise that a gap remains between benchmark performance and real-world molecular development^59,60^. Bridging this divide necessitates metrics tailored for extreme class imbalance, rational dataset splitting strategies that evaluate both inter- and intra-scaffold generalisation, and the accounting of side effects, such as off-target binding liabilities in drug discovery.

Looking ahead, DGT is poised to dedicate to high-throughput molecular screening and discovery, driven by the growing demand for efficient exploration of vast chemical spaces and rapid identification of novel compounds with tailored properties. We foresee DGT advancing deep learning on molecules and constituting a component of the upcoming AI-driven scientific research, improving both the prediction accuracy and interpretability of molecular properties.

## Methods

### Datasets and splitting strategy

We thoroughly evaluated DGT across four domains for molecular property prediction, encompassing ten datasets and a total of 58 subtasks from the MoleculeNet benchmark^26^. Datasets included in the physiology were BBBP, ClinTox, and Tox21, which comprises 12 classification tasks ranging from NR-AR to SR-p53, as well as SIDER, which covers 27 tasks related to adverse drug reactions. BACE and HIV were selected from the biophysics domain to assess classification performance on drug activity and antiviral screening, respectively; For physical chemistry, three regression tasks were employed: ESOL, FreeSolv, and Lipophilicity. Finally, the quantum chemistry domain was represented by the QM9 dataset, comprising 12 regression tasks targeting molecular quantum properties, ranging from basic properties such as dipole moments to electronic properties including frontier orbital energies. Randomly sampled subsets of the PCQM4Mv2 dataset were utilised as the training set for investigating the pretraining of DGT(3D). The chiral classification ability of DGT(R/S) was validated on a subset of PubChem3D released by Adams et al.^61^, which is available at (https://figshare.com/s/e23be65a884ce7fc8543).

We employed the scaffold split^62,63^ method to partition datasets from the MoleculeNet benchmark into training, validation, and test sets with an 80:10:10 ratio. In this approach, splitting is based on the molecular scaffold—essentially the core structure of a molecule, essentially the fundamental ring system or backbone without any substituent groups. Compared to the random split, the scaffold split is more challenging by ensuring that the test set contains molecules with scaffolds that were not encountered during training, thereby requiring the model to generalise to new chemical structures. To ensure a fair comparison between DGT and baseline models, the model training in this work was repeated four times with the same data split to get the average result. The scaffold split method was implemented following the publicly available codes from the chemprop GitHub repository (https://github.com/aamini/chemprop.git).

The BBBP dataset comprises 2039 molecules annotated with binary labels indicating whether each compound can penetrate the blood–brain barrier, a key consideration in central nervous system drug development. The ClinTox dataset contains 1478 compounds categorised according to whether they have been approved or withdrawn from clinical trials due to severe toxicity. Tox21 is a public database consisting of 7831 instances qualitatively evaluated across 12 biological targets, including nuclear receptors and stress response pathways, providing a diverse set of multi-label toxicity prediction tasks. The SIDER dataset maps 1427 marketed drugs to 27 types of clinically reported adverse drug reactions, supporting the association between molecular structures and side-effect profiles for developing safer pharmaceuticals.

The BACE dataset consists of 1513 small-molecule inhibitors annotated for their binding activity toward the human β-secretase 1 enzyme, a critical target in Alzheimer’s disease research. With 41,127 compounds, the HIV dataset provides profound data on molecules’ ability to inhibit HIV replication, offering a large-scale benchmark for antiviral activity classification.

The ESOL dataset collects 1128 molecules with experimentally measured aqueous solubility values, supporting the investigation of how molecular features influence solubility. The FreeSolv dataset is a curated collection of 642 small organic compounds with hydration free energies, serving as a data source for studying drug solubility and stability. Lipophilicity, which describes how well a substance interacts with non-polar environments, is captured in a dataset with 4200 compounds with experimental octanol–water partition coefficients, offering essential data for analysing the pharmacokinetic behaviour.

The quantum mechanics domain is represented by the QM9 dataset, comprising 133,885 small molecules composed of up to nine heavy atoms (C, O, N, and F), each annotated with quantum mechanical properties computed at the B3LYP/6-31 G(2df,p) level of theory. The dataset includes 12 regression targets that capture fundamental quantum molecular properties crucial for versatile domains, with units listed in Table S5. These properties encompass dipole moment, polarisability, squared radius, zero-point vibrational energy, heat capacity at constant volume, highest occupied molecular orbital (HOMO) energy, lowest unoccupied molecular orbital (LUMO) energy, HOMO–LUMO energy gap, internal energy at 0 K, internal energy at standard state, enthalpy, and Gibbs free energy.

A subset of PubChem3D was adopted to validate the introduced atom–atom chiral-volume encoding for chiral centre classification. The dataset comprises multiple OMEGA-generated conformers of organic molecules containing up to 50 heavy atoms and 15 rotatable bonds. The subset consists of 466 K conformers of 78 K enantiomers with one tetrahedral chiral centre. Each stereoisomer has at least two conformers, and each graph has at least two stereoisomers. The dataset was partitioned into training, validation, and testing sets using a 70/15/15 split while ensuring that all conformers associated with the same 2D graphs were assigned to the same data partition.

### Experimental settings

The experimental setting section consists of evaluation metrics and pretraining setup.

We benchmarked DGT across 58 molecular property prediction tasks spanning four distinct domains, each evaluated using domain-appropriate metrics. The AUC-ROC was utilised for classification tasks within the physiology and biophysics domains, as it quantifies a model’s ability to distinguish between classes across various threshold settings; a higher AUC-ROC value indicates better classification performance. Notably, for multi-label classification tasks, the macro-average AUC-ROC is adopted, where the AUC is first calculated for each class separately and subsequently averaged across all classes, thereby providing a balanced evaluation of model performance across heterogeneous labels. For physical chemistry, the RMSE was adopted to assess the difference between predicted and true values. MAE was employed for the quantum mechanics domain as a routine metric to measure the average magnitude of prediction errors.

We investigated the applicability of the pretraining strategy on DGT(3D) using the large-scale PCQM4Mv2 dataset^43^. In this setup, DGT(3D) was first pretrained on the PCQM4Mv2 dataset to predict the HOMO–LUMO energy gap, a relevant task intended to enhance the downstream prediction of HOMO and LUMO energies within the QM9 dataset. To assess the impact of the pretraining dataset size, we carried out a series of pretraining runs using subsets with 10 K, 100 K, and 1 M molecules randomly sampled from PCQM4Mv2. DGT(3D) was pretrained on these sampled molecules for 100 epochs incorporating a linear warmup strategy over the first 5 epochs, using the AdamW optimiser with a batch size of 128 and a small learning rate of 0.0002, scheduled via cosine annealing. The resulting pretrained weights were then utilised to initialise DGT(3D) for training on the QM9 dataset. Specifically, PCQM4Mv2 involves a total of 22 elements, covering those present in the QM9 dataset (H, C, O, N, and F); relevant atomic embeddings were accordingly extracted and concatenated to initialise the atom embedding matrix for QM9.

### DGT modules

For the DGT architecture, atom encodings are constructed from a comprehensive suite of categorical descriptors—including atomic number, chirality, formal valency, hybridisation state, aromaticity, and a ring membership indicator. Parallel to this, bond encodings of DGT encompass bond order, stereoisomerism type, and a conjunction indicator. DGT can be supplemented with bond lengths, atom–atom distances, and bond–bond angles as 3D molecular descriptors. Additionally, chiral volume and inter-bond *E/Z* isomeric descriptor can be captured as stereogeometric information for DGT.

SPDE and RWPE are employed as node relative positional encodings. SPDE assign each node–node pair an embedding vector based on its shortest path length in the molecular graph. RWPE encodes a node’s position through the distribution of random walks originating from that node, thereby capturing multi-hop connectivity patterns into the embedding space (Supplementary Note 1). For the bond–bond angle encoding, we employed the normalised spherical harmonics to provide an orthonormal representation of angular dependencies (Supplementary Note 2).

Specifically, the chiral volume is defined as the scalar triple product of the vectors originating for a directed bond from a stereocentre. To ensure a unique and physically meaningful value, its three neighbouring bonds are ordered as vectors ${{{\boldsymbol{\upsilon }}}}_{1}$, ${{{\boldsymbol{\upsilon }}}}_{2}$ and ${{{\boldsymbol{\upsilon }}}}_{3}$ according to the Cahn-Ingold-Prelog (CIP) rules^64^. The signed chiral volume is then calculated as $({{{\boldsymbol{\upsilon }}}}_{1}\times {{{\boldsymbol{\upsilon }}}}_{2})\cdot {{{\boldsymbol{\upsilon }}}}_{3}$. The inter-bond *E/Z* isomeric descriptor embeds the relative *cis/trans* orientation of vicinal bonds across the double bond into the bond–bond encodings.

The feature fusion module provides a mutual extension of atom and bond encodings. It concatenates the feature vector of each bond with the mean representations of its incident atoms; likewise, each atomic feature vector is appended with the average of its adjacent bond features. Formally, we define atom and bond graphs as ${{{\mathscr{G}}}}_{a}=({{{\mathscr{N}}}}_{a},{{{\mathscr{E}}}}_{a})$ and ${{{\mathscr{G}}}}_{b}=({{{\mathscr{N}}}}_{b},{{{\mathscr{E}}}}_{b})$, respectively, where ${{{\mathscr{N}}}}_{a}$ indicates the set of atoms, ${{{\mathscr{E}}}}_{a}\subseteq {{{\mathscr{N}}}}_{a}\times {{{\mathscr{N}}}}_{a}$ the set of directed bonds, ${{{\mathscr{N}}}}_{b}$ the set of undirected bonds, and ${{{\mathscr{E}}}}_{b}\subseteq {{{\mathscr{N}}}}_{b}\times {{{\mathscr{N}}}}_{b}$ the set of neighbour pairs of undirected bonds. Given the atom feature dimension size as ${d}_{a}$, the atom features are encoded as rows in a matrix ${{{\bf{N}}}}_{a}\in {{\mathbb{R}}}^{\left|{{{\mathscr{N}}}}_{a}\right|\times {d}_{a}}$; likewise, the bond features are encoded as ${{{\bf{N}}}}_{b}\in {{\mathbb{R}}}^{\left|{{{\mathscr{N}}}}_{b}\right|\times {d}_{b}}$, and ${d}_{b}$ is the bond feature dimension size. The feature fusion operation can be expressed as:

1 $${{{\bf{N}}}}_{a,i}={{{\bf{N}}}}_{a,i\,\,}^{0}{{\boldsymbol{\parallel }}}\frac{1}{\left|{{{\mathcal{N}}}}_{b,i}\right|}{\sum}_{j\in {{{\mathcal{N}}}}_{b,i}}{{{\bf{N}}}}_{b,j}^{0}$$

2 $${{{\bf{N}}}}_{b,i}={{{\bf{N}}}}_{b,i\,\,}^{0}{{\boldsymbol{\parallel }}}\frac{1}{2}{\sum}_{j\in {{{\mathcal{N}}}}_{a,i}}{{{\bf{N}}}}_{a,j}^{0}$$

where ${{{\bf{N}}}}_{a,i}$ and ${{{\bf{N}}}}_{b,i}$ indicate the *i*-th node embedding of atom and bond graphs, respectively; ${{{\bf{N}}}}_{a,i}^{0}$ and ${{{\bf{N}}}}_{b,i}^{0}$ their counterparts before concatenation; ${{{\mathscr{N}}}}_{b,i}$ the index set of bonds incident to the *i*-th atom; and ${{{\mathscr{N}}}}_{a,i}$ the index set of the pair of atoms terminating the *i*-th bond. After the concatenation operation, multilayer perceptron (MLP) is applied to both atom and bond encodings for feature fusion.

The biased multi-head attention mechanism can be formulated as:

3 $${a}_{i,j,k}=exp\left(\frac{{{{\bf{Q}}}}_{i}\cdot {{{\bf{K}}}}_{j}}{\sqrt{d}}+{e}_{i,j,k}^{att}\right)$$

4 $${s}_{i,j,k}={a}_{i,j,k}/{\sum}_{j}{a}_{i,j,k}$$

5 $${{{\bf{H}}}}_{i}={\sum }_{j}{{{\bf{S}}}}_{i,j}\odot ({{{\bf{V}}}}_{j}+{{{\bf{E}}}}_{i,j}^{val})$$

where ${{\bf{Q}}}$, ${{\bf{K}}}$, and ${{\bf{V}}}$ are respective projections of the input ${{\bf{X}}}$ for query, key, and value; ${{{\bf{E}}}}^{{att}}$ and ${{{\bf{E}}}}^{{val}}$ are respective projections of the node–node encoding ${{\bf{E}}}$ to attention bias and edge feature injection. d, which denotes dimensionality of each head, is involved for stabilising the training process. ${a}_{i,j,k}$ is the calculated attention of node *i* to node *j* at the hidden index *k*, and ${s}_{i,j,k}$ is the corresponding attention score. ${{\bf{H}}}$ is the output hidden embedding.

The node–node encoding ${{\bf{E}}}$ is the sum of encodings of relative position, ring structure and stereogeometric descriptors. DGT(3D) incorporates bond lengths to ${{{\bf{N}}}}_{b}$, atom–atom distances into ${{{\bf{E}}}}_{a}$, and bond–bond angles to ${{{\bf{E}}}}_{b}$. To inform the model with enriched local geometric representation, bond angular information is encoded with spherical harmonics functions defined on the unit sphere to ensure rotational consistency^65^. In addition to that, chiral volume and *E/Z* isomerism information are integrated into ${{{\bf{E}}}}_{a}$ and ${{{\bf{E}}}}_{b}$, respectively, as stereogeometric descriptors to enhance DGT’s performance on stereoselective tasks.

With this attention calculation hereafter referred to as ${{\rm{MHA}}}({{\bf{X}}},\,{{{\bf{E}}}}^{{att}},\,{{{\bf{E}}}}^{{val}})$, the DGT layer can be given as:

6 $$\bar{{{\bf{H}}}}={{\rm{BatchNorm}}}\left({{\bf{X}}}+{{\rm{MHA}}}\left({{\bf{X}}},\,{{{\bf{E}}}}^{{att}},\,{{{\bf{E}}}}^{{val}}\right)\right)$$

7 $$\bar{{{\bf{X}}}}={{\rm{BatchNorm}}}\left({{\rm{FFN}}}\left(\bar{{{\bf{H}}}}\right)\right)$$

Here, the input hidden node feature matrix **X** is added to ${{\rm{MHA}}}({{\bf{X}}},\,{{{\bf{E}}}}^{{att}},\,{{{\bf{E}}}}^{{val}})$ and transformed by MLP. Feed forward network (FFN) follows to implement nonlinear transformations to each token embedding to obtain the output node features matrix $\bar{{{\bf{X}}}}$. The batch normalisation (BatchNorm) is applied to avoid overfitting.

For the readout part, atom and bond features are globally averaged and concatenated for predicting the molecular property as ${{\rm{MLP}}}({{\rm{GAP}}}({{{\bf{X}}}}_{a}){{\boldsymbol{\parallel }}}{{\rm{GAP}}}({{{\bf{X}}}}_{b}))$, where GAP is the global average pooling operation. Activation function is attached after each hidden layer of the MLP.

### Baselines

DGT was comprehensively compared with state-of-the-art machine learning methods on datasets from the MoleculeNet benchmark. Random forest (RF) is adopted as a baseline machine learning model^26^. We also compared DGT with some advanced graph neural network (GNN) models, including Attentive FP^15^, D-MPNN^27^, pretrained GNN^28^, GraphMVP^29^, MolCLR^30^, and MoleBERT^31^. GROVER presented as a representation model for the graph transformer^32^. UniMol was included as a representation of transformer-based 3D geometric deep learning models for molecular property prediction^33^. Performances of GraphMVP, MolCLR, MoleBERT, GROVER, and UniMol are derived from ref.^66^ with the same data splitting. Attentive FP and D-MPNN is implemented using the DeepChem package (https://github.com/deepchem/deepchem.git), random forest is based on scikit-learn (https://github.com/scikit-learn/scikit-learn.git), and pretrained GNN is reproduced according to (https://github.com/snap-stanford/pretrain-gnns.git).

In detail, RF employs ECFP4 as input features—a widely adopted circular fingerprint in cheminformatics and molecular machine learning, which encodes molecules by capturing local atom-centred substructures. Attentive FP extends conventional GNNs by incorporating the attention mechanism that dynamically adjusts the importance weights of neighbouring atoms and bonds during message passing. D-MPNN refines standard message passing neural networks by directing messages over bonds instead of atoms, which improves the encoding of bond-level interactions critical for accurate molecular property prediction. The pretrained GNN baseline demonstrates the performance gains of a GNN architecture by informing it with large-scale molecular datasets before fine-tuning on downstream tasks. GraphMVP leverages a multi-view contrastive learning strategy, aligning information from the 2D molecular graph and its corresponding 3D conformer to encourage the model to learn representations consistent across different structural modalities.

MolCLR further advances contrastive learning by generating diverse graph augmentations to improve the robustness of learnt features against input perturbations. MoleBERT adapts language model pretraining principles to molecular graphs by introducing masked atom and bond prediction tasks, facilitating the learning of versatile graph-level encoders that can generalise across molecular prediction tasks. GROVER combines self-supervised learning objectives with a graph transformer backbone, learning rich contextualised molecular representations from millions of unlabelled molecules.

For 3D molecular learning, DGT(3D) was benchmarked against SchNet^38^, DimeNet^39^, SphereNet^40^, and SE(3) Transformer^41^, besides GraphMVP^29^ and 3DInfomax^42^ that leverage 3D information to enrich 2D molecular embedding. SchNet represents one of the earliest deep learning architectures explicitly designed for molecules and materials, incorporating continuous-filter convolutional layers to directly model quantum interactions. By treating interatomic distances as continuous inputs, SchNet effectively captures long-range dependencies for predicting quantum mechanical properties. Based on that, DimeNet introduces directional message passing by encoding angular information between bonded atoms, thereby enabling the model to learn higher-order geometric interactions. SphereNet extends this paradigm by leveraging spherical message passing, integrating both angular and radial basis functions to achieve a more complete characterisation of local geometric environments. The SE(3) Transformer generalises attention mechanisms to 3D Euclidean space by enforcing equivariance under SE(3) group transformations, allowing the model to learn molecular representations invariant to rotations and translations while preserving geometric symmetries. Packages for implementing these compared models are as follows: SchNet and DimeNet, (https://github.com/pyg-team/pytorch_geometric.git); SphereNet, (https://github.com/divelab/DIG.git); SE(3) Transformer, (https://github.com/chao1224/Geom3D.git).

## Supplementary information

Supplementary Information

Transparent Peer Review file

## Data Availability

Datasets used for benchmarking the DGT model are available at (https://moleculenet.org/datasets). The PCQM4Mv2 dataset was used in pretraining DGT(3D), which is accessible via (https://ogb.stanford.edu/docs/lsc/pcqm4mv2). The PubChem3D subset for chiral classification can be found at (https://figshare.com/s/e23be65a884ce7fc8543). Source data are available at (10.6084/m9.figshare.30665129).

## Code availability

All codes are available via GitHub at (https://github.com/zhangsy-ryan/DGT.git) or Zenodo at (10.5281/zenodo.20009509).
