# Supplementary material for: Enhancing molecular property prediction of transformer models with dual graph representation
Source: Nat Commun. 2026 Jun 27;17:8039. doi: 10.1038/s41467-026-75005-9 (PMC13454579; doi:10.1038/s41467-026-75005-9)
Supplement: Supplementary file 1 [file 41467_2026_75005_MOESM1_ESM.pdf]

Supplementary Information

Enhancing molecular property prediction of transformer models with dual graph representation

Shuyuan Zhang[1,2,*] and Alexei A. Lapkin[1,2]

[1]Department of Chemical Engineering and Biotechnology, University of Cambridge, Philippa Fawcett Drive, Cambridge, CB3 0AS, UK

[2]Innovation Centre in Digital Molecular Technologies, Yusuf Hamied Department of Chemistry, University of Cambridge, Lensfield Road, Cambridge, CB2 1EW, UK

[*]Corresponding author.
E-mail address: sz469@cam.ac.uk

Table of Contents

Suppl. Note 1. Graph-specific encodings 3
Suppl. Note 2. Angle embedding 5
Suppl. Note 3. DGT layer architecture 6
Suppl. Note 4. Dataset information 7
Suppl. Note 5. DGT configurations for all tasks 8
Suppl. Note 6. Performance benchmark details 9
Suppl. Note 6.1 Detailed performance benchmark of DGT in the physiology domain 9
Suppl. Note 6.2 Detailed performance benchmark of DGT in the biophysics domain 9
Suppl. Note 6.3 Detailed performance benchmark of DGT in the physical chemistry domain 10
Suppl. Note 6.4 Detailed performance benchmark of DGT in the quantum mechanics domain 11
Suppl. Note 7. Detailed performance comparison of DGT(3D) against other models 12
Suppl. Note 8. Impact of pretraining data scale on DGT(3D) performance 13
Suppl. Note 9. Model parameter amounts and FLOPs during inference 14
Suppl. Note 10. Performance benchmark of DGT(R/S) for chiral classification 15
Suppl. Note 11. Units for QM9 tasks 16
Suppl. Note 12. Attention-based interpretation 17
Supplementary References 18

Suppl. Note 1. Graph-specific encodings

Relative position encoding (RPE)[1] and ring structural encoding (RSE)[2] are involved to introduce topological and molecular modular features to the attention mechanism calculation. Shortest path distance encoding (SPDE)[3] and random walk positional encoding (RWPE)[4] are employed as the RPE of nodes.

In SPDE, a node–node pair is assigned an embedding vector derived from the length of its shortest path in the underlying molecular graph. SPDE encodes higher-order connectivity patterns by differentiating between nodes that are directly connected, separated by a few edges, or positioned further apart in the molecular graph. Such a representation provides a natural means of integrating long-range structural information. In DGT, shortest-path distances are mapped to a discrete embedding space using learnable vectors, enabling the model to assign distinct representations to different edge lengths (e.g., 1-hop, 2-hop, etc.) while retaining the flexibility to adapt during training.

In RWPE, the position of a node is encoded by the distribution of random walks originating from it, integrating multi-hop connectivity patterns into the embedding space. RWPE is able to provide a probabilistic description of a node’s neighborhood that naturally reflects both local and global graph topology. Formally, we consider the random walk transition matrix $\mathrm{T} = \mathrm{D}^{-1}\mathrm{A}$, where A is the adjacent matrix of the graph and D is the degree matrix. The k-step random walk distribution can be then given as T^{k}. The embedding for a node–node pair is obtained by aggregating its visitation probabilities over multiple walk lengths. This construction yields positional features that are permutation-equivariant and expressive for downstream neural architectures.

The representation of cyclic substructures plays a critical role in molecular machine learning, as conventional atom- and bond-level graph embeddings often fail to capture the higher-order connectivity and geometric constraints imposed by ring systems. By embedding the ring size as learnable vectors, we introduce RSE to encode ring systems as distinct, information-rich entities within molecular graphs, producing a latent representation that complements the node-level embedding.

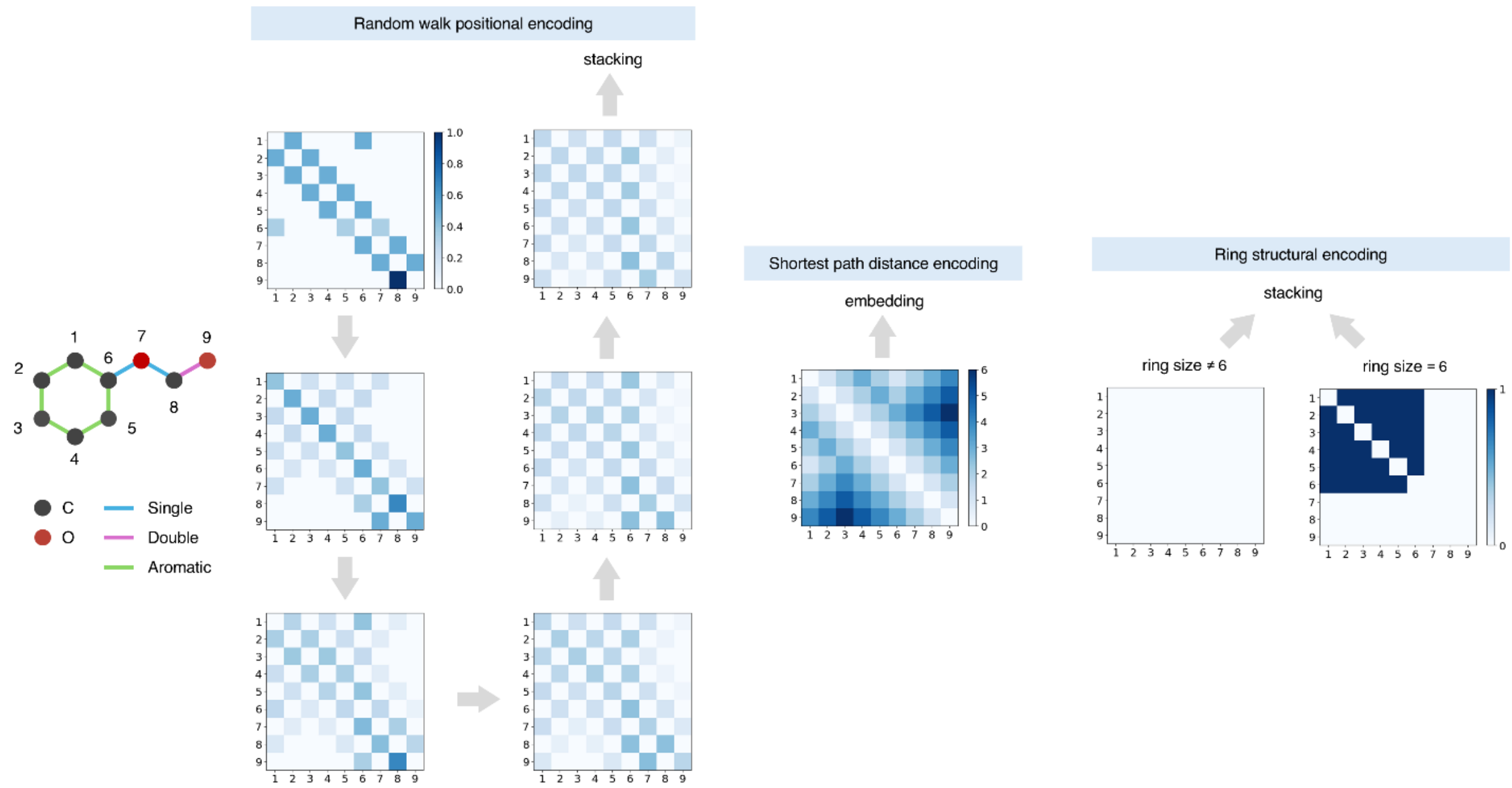

Supplementary Fig. S1 Illustration of graph-specific encodings of the example phenyl formate molecule. Random walk positional and shortest path distance encodings are included as relative positional encodings. Rings of different sizes are identified in the molecule and then stacked as node–node pairwise features.

Suppl. Note 2. Angle embedding

The representation of continuous geometric information in an expressive and computationally tractable form is a central challenge in molecular machine learning[5]. Directly using raw angles as features often fails to capture the spatial relationship. To overcome this limitation, we employ normalised spherical harmonics Y_l^m to represent bond–bond angles to provide an orthonormal representation of angular dependencies.

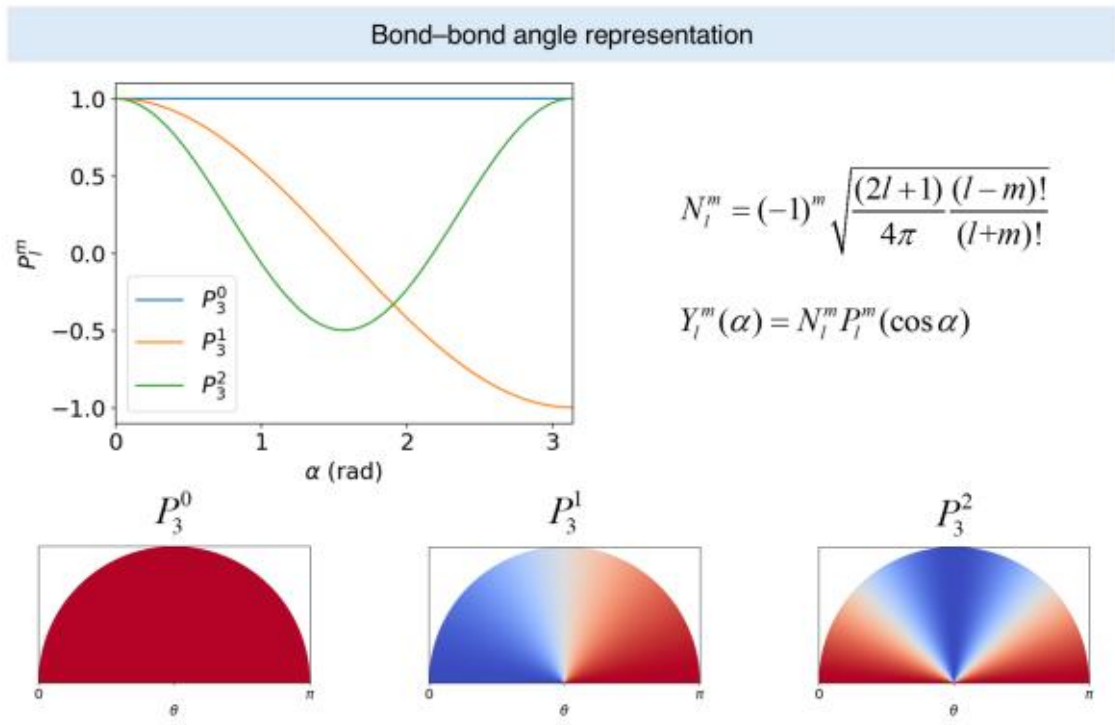

Supplementary Fig. S2 Embedding of bond–bond angles. The bond–bond angle is embedded with normalised spherical harmonics Y_l^m, where N_l^m is the pre-factor and P_l^m is the associated Legendre polynomials.

Suppl. Note 3. DGT layer architecture

The DGT layer highlights the incorporation of node pairwise features into of the attention score and the passing message. Unlike standard self-attention mechanisms that rely on node embeddings, DGT introduces pairwise terms that encode topological and structural information between nodes for both atom and bond graphs. In practice, the attention coefficient $s_{i,j}$ between two nodes i and j is computed as

$$a_{i,j} = \exp\left(\frac{Q_i \cdot K_j}{\sqrt{d_k}} + E_{i,j}^{att}\right)$$

$$s_{i,j} = a_{i,j} \Big/ \sum_j a_{i,j}$$

where Q_i and K_j denote the query and key projections of the respective node embeddings, and $E_{i,j}^{att}$ is a learnable bias term derived from pairwise features such as SPDE, RWPE, and RSE.

During message aggregation, the pairwise features are further integrated by modulating the passing message:

$$h_i = \sum_j s_{i,j}\left(V_j + E_{i,j}^{val}\right)$$

Similarly, $E_{i,j}^{val}$ is also a learnable term derived from pairwise features such as SPDE, RWPE, and RSE for enriching the passing message.

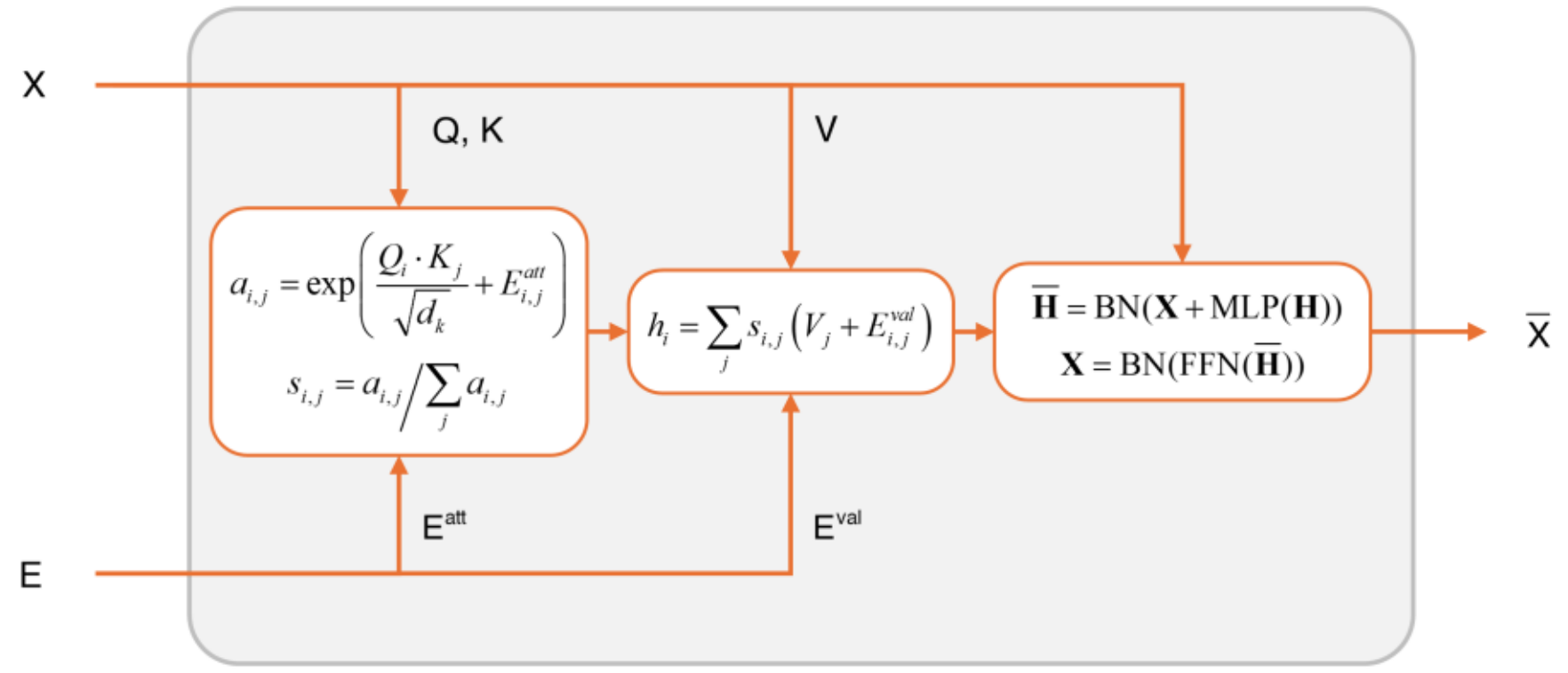

Supplementary Fig. S3 Detailed DGT layer architecture. This DGT layer highlights the incorporation of node–node pairwise features into both attention score calculation and passing message. Notably, only the node feature matrix is layer-wisely updated; the node–node pairwise feature matrix is the same for all DGT layers. Q, K, and V denote the query, key, and value in the self-attention mechanism, while E^{att} and E^{val} represent edge features incorporated into the attention and value computations, respectively. d_k is the dimensionality of each head, $s_{i,j}$ the calculated attention score, and h_i the updated hidden vector.

Suppl. Note 4. Dataset information

Supplementary Table 1 Statistical information of MoleculeNet datasets[6] for benchmarking DGT.

Domain	Dataset	Type	Tasks	Compounds	Metric
Physiology	BBBP	Classification	1	2,053	ROC-AUC
	Tox21	Classification	12	8,014	ROC-AUC
	ClinTox	Classification	2	1,491	ROC-AUC
	SIDER	Classification	27	1,427	ROC-AUC
Biophysics	BACE	Classification	1	1,522	ROC-AUC
	HIV	Classification	1	41,913	ROC-AUC
Physical chemistry	ESOL	Regression	1	1,128	RMSE
	FreeSolv	Regression	1	643	RMSE
	Lipophilicity	Regression	1	4,200	RMSE
Quantum mechanics	QM9	Regression	12	133,885	MAE

Suppl. Note 5. DGT configurations for all tasks

Supplementary Table 2 DGT configuration parameters for different datasets.

Dataset	Batch size	Layers	Heads	Hidden size	Learning rate	Randon walk steps	Max shortest path	Max ring size
BBBP	32	4	16	128	4e-4	16	8	18
Tox21	32	5	16	128	1e-4	8	6	8
ClinTox	16	3	32	128	1e-1	6	6	6
SIDER	8	5	16	64	1e-4	-	12	-
BACE	128	5	8	64	2e-5	-	6	6
HIV	16	5	16	128	1e-5	-	8	18
ESOL	128	6	32	128	5e-5	16	8	18
FreeSolv	64	4	16	128	5e-5	6	-	6
Lipophilicity	24	5	48	384	5e-5	16	6	6
QM9	256	10	16	128	2e-4	16	8	18

Note: DGT(3D) is applied with the same configuration parameters as DGT on the QM9 dataset.

Suppl. Note 6. Performance benchmark details

Suppl. Note 6.1 Detailed performance benchmark of DGT in the physiology domain

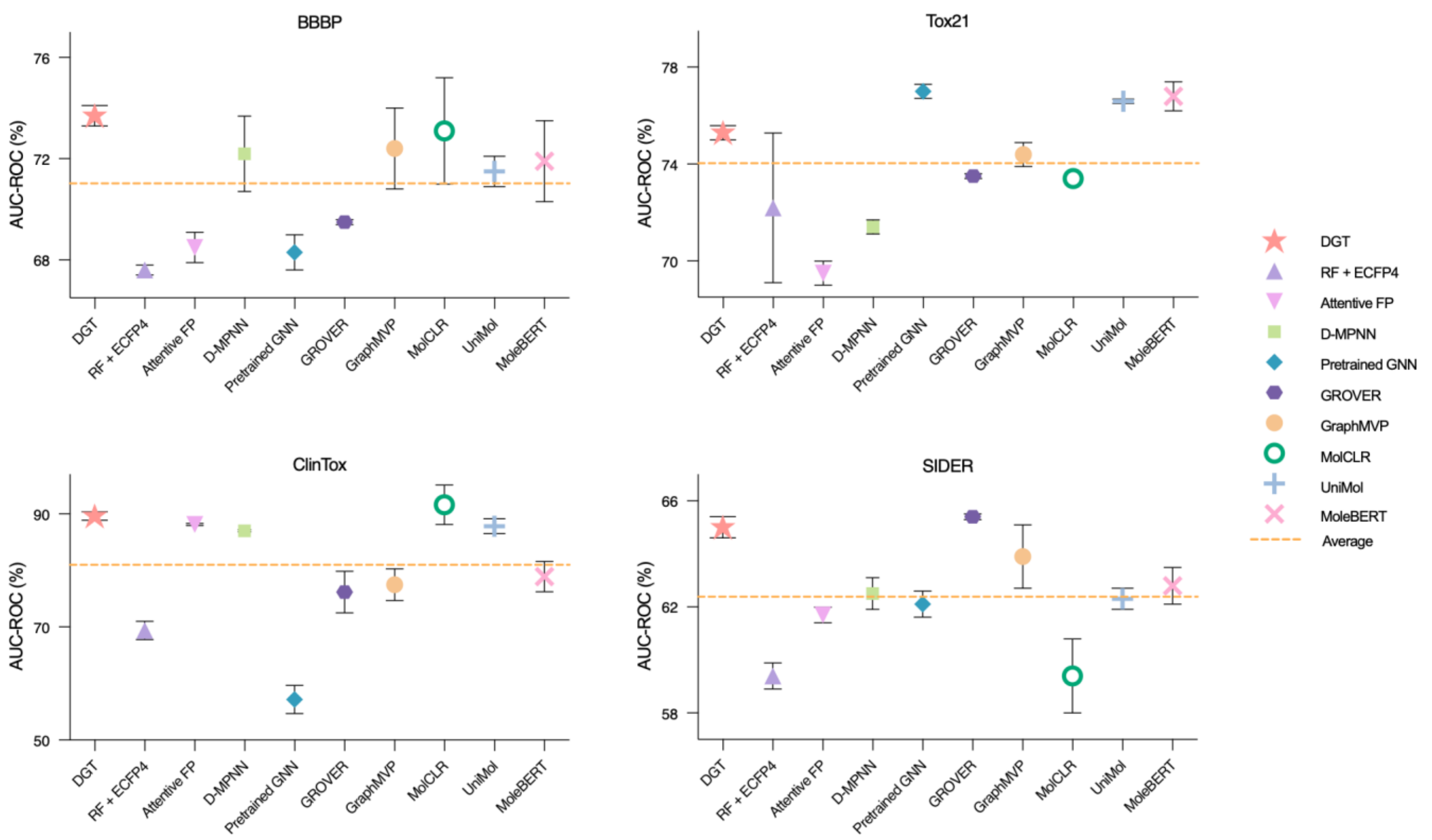

Supplementary Fig. S4 Detailed Performance comparison of DGT against nine baselines on BBBP, Tox21, ClinTox, and SIDER datasets within the physiology domain. DGT achieves the best performance on BBBP and ranks second on ClinTox and SIDER. On Tox21, DGT remains highly competitive, with performance slightly below that of Pretrained GNN, UniMol, and MolBERT. All error bars represent the standard deviation derived from four runs. Source data are provided as a Source Data file.

Suppl. Note 6.2 Detailed performance benchmark of DGT in the biophysics domain

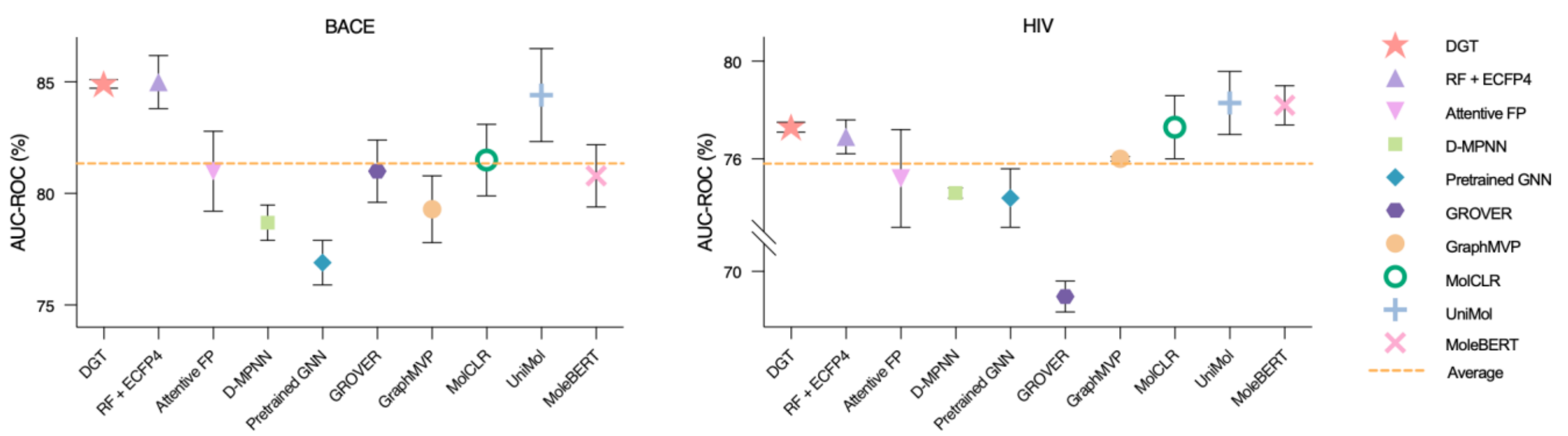

Supplementary Fig. S5 Detailed Performance comparison of DGT against nine baselines on BACE and HIV datasets within the biophysics domain. DGT delivers competitive performance against other best-performance models in both datasets: BACE, RF + ECFP4 and UniMol; HIV, UniMol and

MoleBERT. All error bars represent the standard deviation derived from four runs. Source data are provided as a Source Data file.

Suppl. Note 6.3 Detailed performance benchmark of DGT in the physical chemistry domain

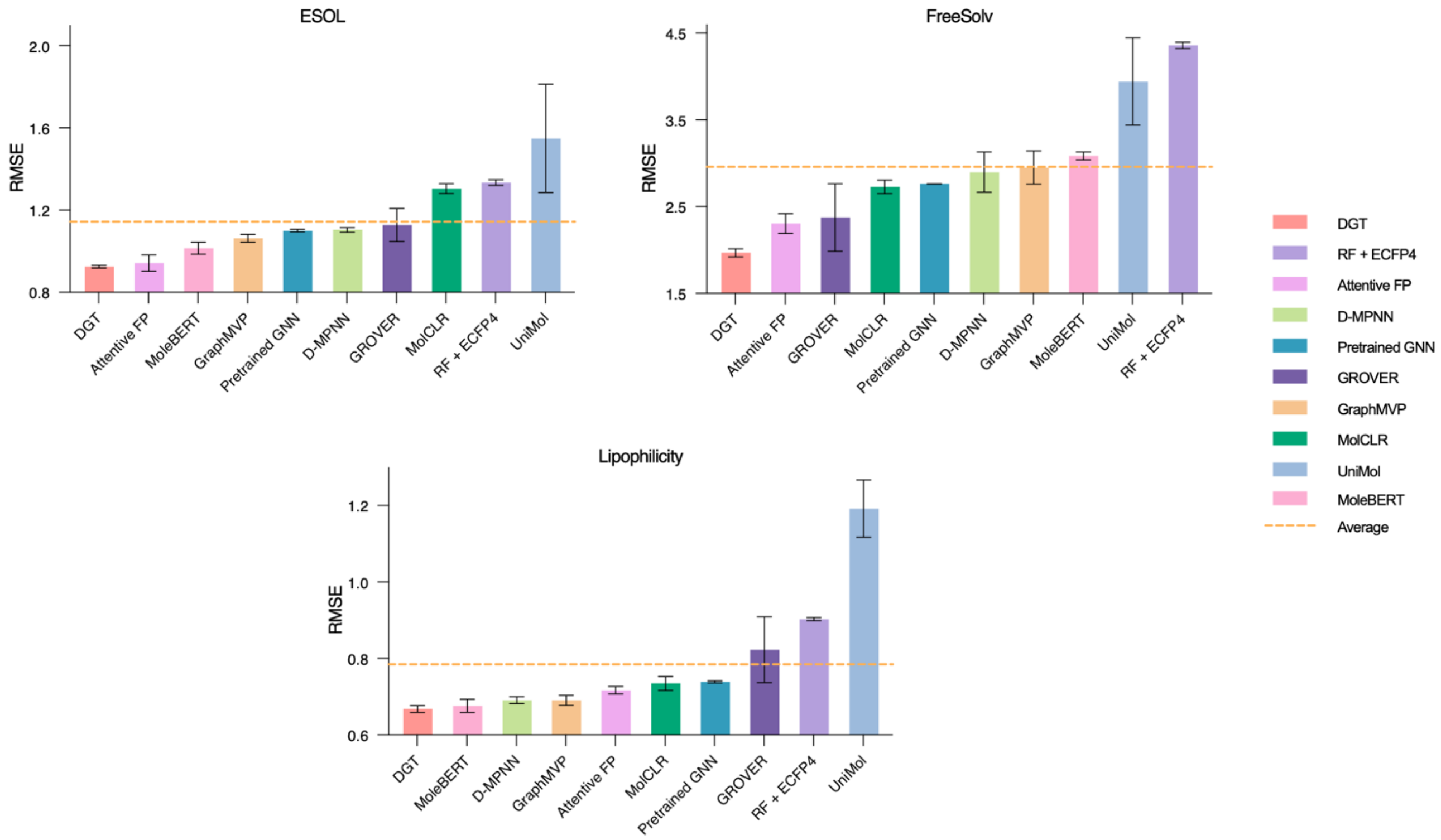

Supplementary Fig. S6 Detailed Performance comparison of DGT against nine baselines on ESOL, FreeSolv, and Lipophilicity datasets within the physical chemistry domain. DGT achieves the best performance except the Lipophilicity dataset, where it slightly underperforms MolBERT. All error bars represent the standard deviation derived from four runs. Source data are provided as a Source Data file.

Suppl. Note 6.4 Detailed performance benchmark of DGT in the quantum mechanics domain

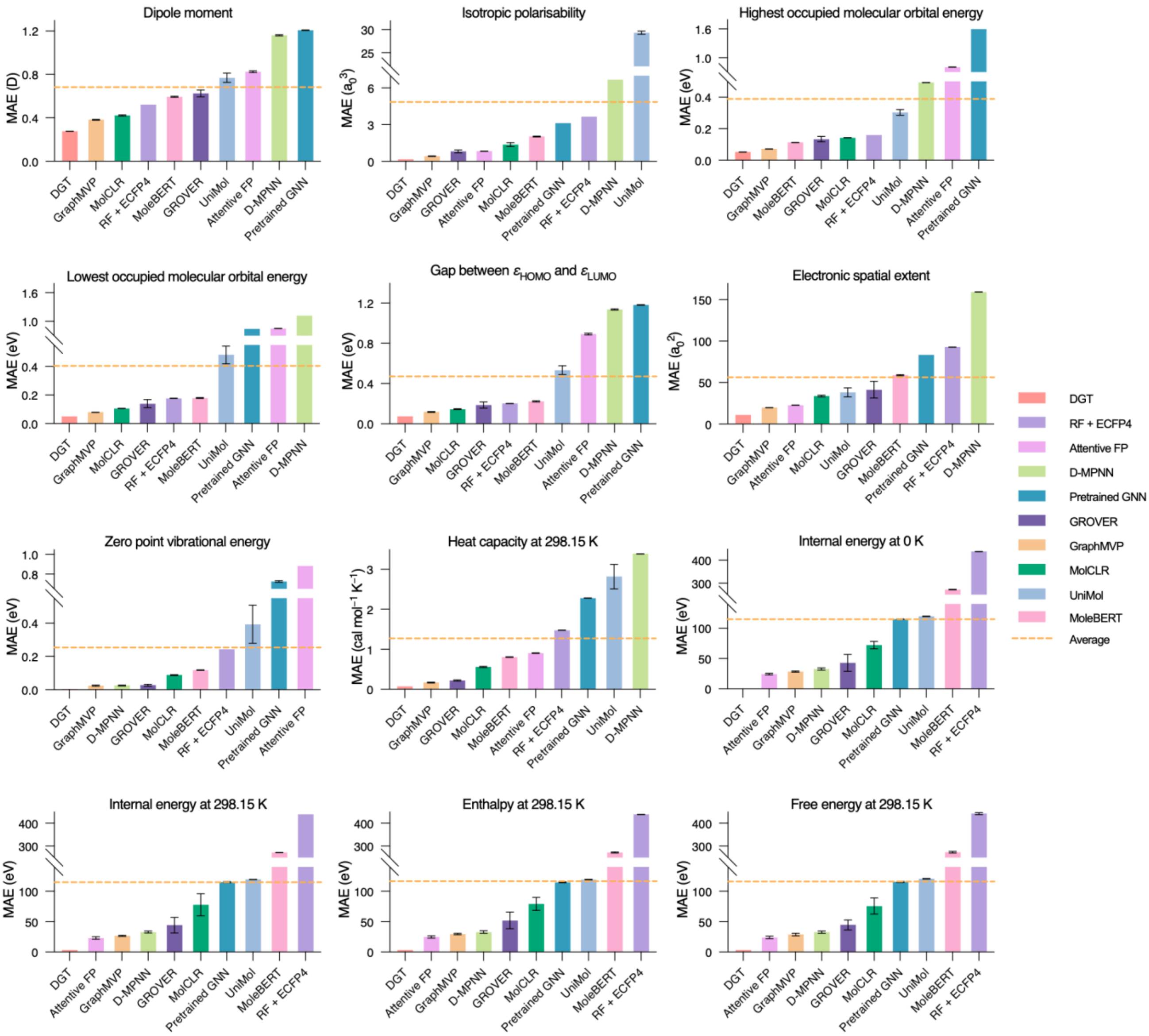

Supplementary Fig. S7 Detailed Performance comparison of DGT against nine baselines on the 12 tasks of the QM9 dataset within the quantum mechanics domain. DGT achieves the best performance on all tasks. All error bars represent the standard deviation derived from four runs. Source data are provided as a Source Data file.

Suppl. Note 7. Detailed performance comparison of DGT(3D) against other models

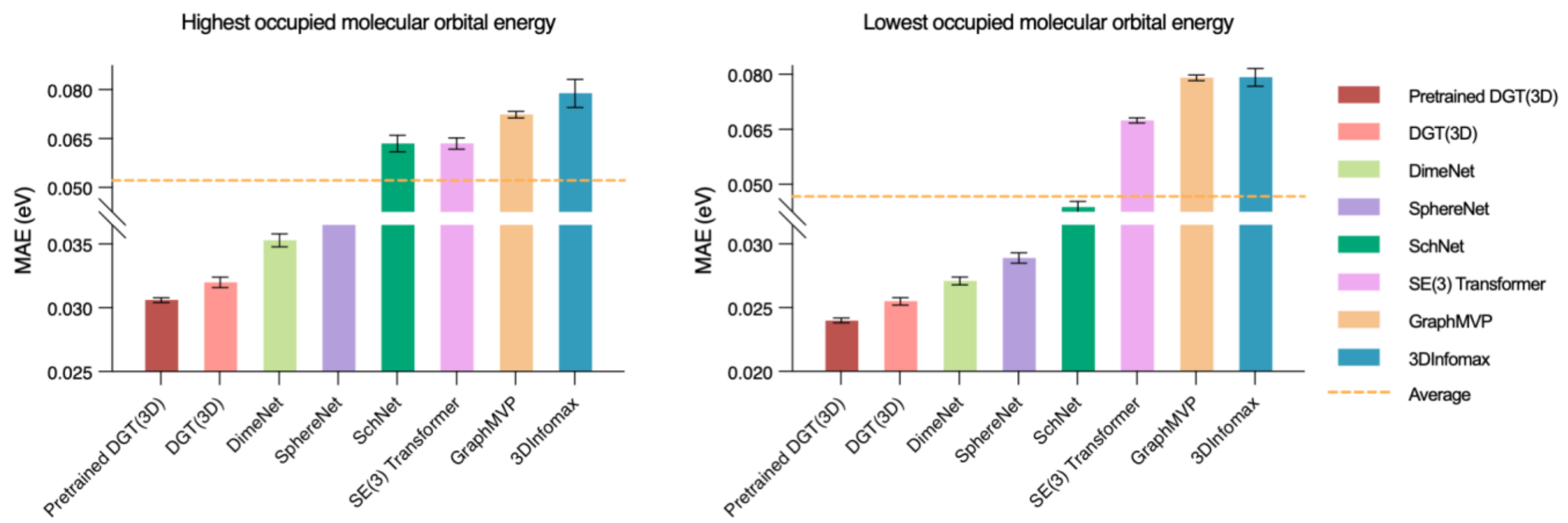

Supplementary Fig. S8 Detailed performance comparison of DGT(3D) against SchNet, DimeNet, SphereNet, SE(3) Transformer, GraphMVP, and 3DInfomax on HOMO and LUMO energy prediction tasks of the QM9 dataset. All error bars represent the standard deviation derived from four runs. Source data are provided as a Source Data file.

Suppl. Note 8. Impact of pretraining data scale on DGT(3D) performance

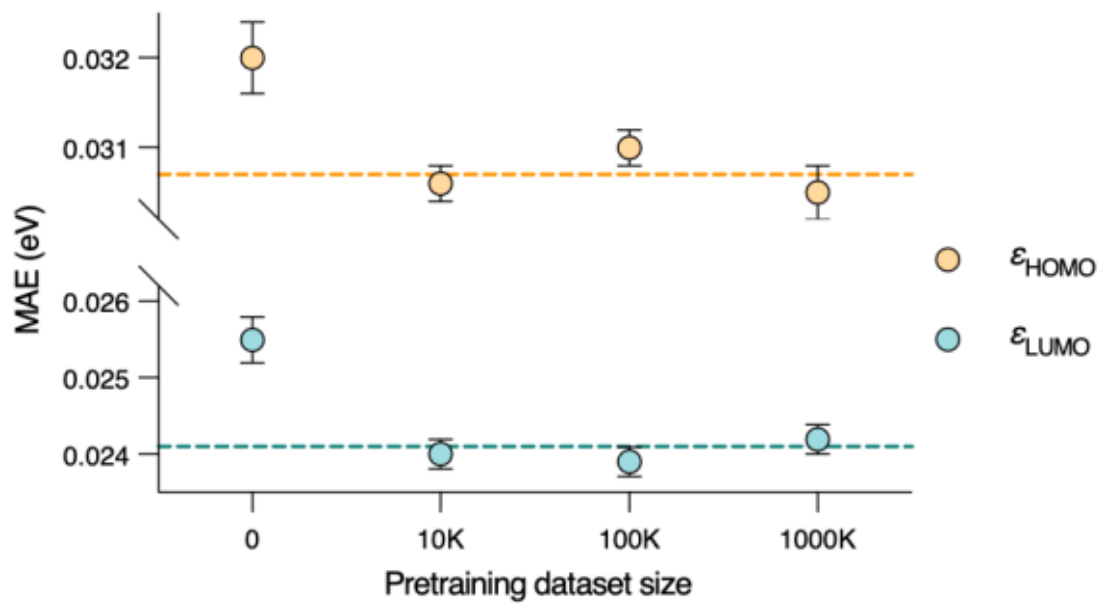

Supplementary Fig. S9 Impact of pretraining data scale on DGT(3D) performance. Performance trajectory of DGT(3D) is evaluated when pretrained on subsets of the PCQM4Mv2 dataset[7] of increasing size (0, 10K, 100K, and 1M molecules) for predicting ε_{HOMO} and ε_{LUMO}. All error bars represent the standard deviation derived from four runs. Source data are provided as a Source Data file.

Supplementary Table 3 Statistical information of the PCQM4Mv2 dataset[7] and sampled subsets.

Dataset	$\Delta\varepsilon$ average (eV)	$\Delta\varepsilon$ standard deviation (eV)	Compounds
PCQM4Mv2	5.6843	1.1646	3,378,606
PCQM4Mv2-subset-10K	5.6171	1.1169	10,000
PCQM4Mv2-subset -100K	5.6152	1.1338	100,000
PCQM4Mv2-subset -1000K	5.6181	1.1382	1,000,000

Suppl. Note 9. Model parameter amounts and FLOPs during inference

Supplementary Table 4 Summary of model parameter amounts and floating-point operations (FLOPs) during inference. The value of FLOPs is benchmarked on methane, which is the first molecule of the QM9 dataset.

Model	Parameter amount	FLOPs
DGT	2.9M	28M
RF + ECFP4	13.3M	0.02M
Attentive FP	0.2M	2M
D-MPNN	0.4M	6M
Pretrained GNN	1.9M	25M
GROVER	~100M	~1G
GraphMVP	5.8M	6M
MolCLR	1.0M	5M
UniMol	~90M	~140M
MoleBERT	1.8M	5M
DimeNet	1.8M	2M
SphereNet	1.8M	3M
SchNet	1.6M	8M
SE(3) Transformer	1.4M	9M
3DInfomax	2.1M	14M

Note: Parameters and FLOPs for DGT are based on the QM9 model configuration. The computational overhead of DGT(3D) is close to DGT due to their identical core structure of stacked graph transformer layers.

Suppl. Note 10. Performance benchmark of DGT(R/S) for chiral classification

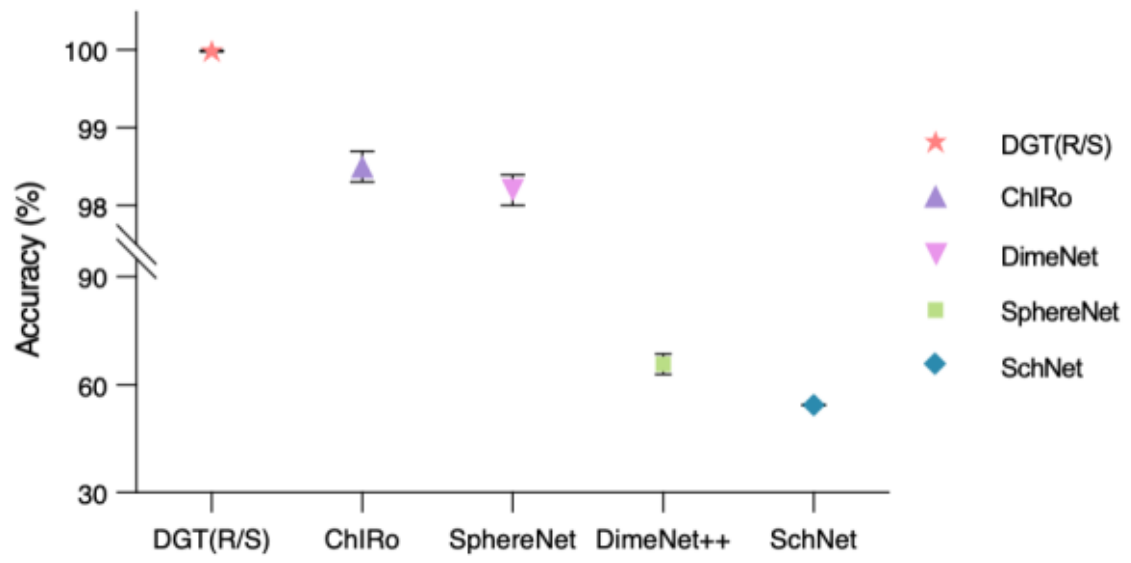

Supplementary Fig. S10 Performance benchmark of DGT(R/S) for chiral classification on the PubChem3D subset against ChIRo, DimeNet, SphereNet and SchNet. Mean DGT(R/S) performance is derived from four independent experimental runs. Performance metrics of other models are adapted from ref. 8. The performance advantage of DGT(R/S) primarily arises from the explicit encoding of chiral volume based on the Cahn-Ingold-Prelog ordering rules, whereas other models must implicitly infer a canonical ordering of substituents around a stereocentre from atom identities and geometric information. All error bars represent the standard deviation derived from four runs. Source data are provided as a Source Data file.

Suppl. Note 11. Units for QM9 tasks

Supplementary Table 5 Units for QM9 tasks.

QM9 task	Unit	Description
μ	Debye (D)	Dipole moment
α	Bohr^3 (a_0^3)	Isotropic polarisability
$\varepsilon_{\mathrm{HOMO}}$	eV	Highest occupied molecular orbital energy
$\varepsilon_{\mathrm{LUMO}}$	eV	Lowest unoccupied molecular orbital energy
$\Delta\varepsilon$	eV	Gap between $\varepsilon_{\mathrm{HOMO}}$ and $\varepsilon_{\mathrm{LUMO}}$
$\langle R^2 \rangle$	Bohr^2 (a_0^2)	Electronic spatial extent
ZPVE	eV	Zero point vibrational energy
c_v	cal/(mol·K)	Heat capacity at 298.15 K
U_0	eV	Internal energy at 0 K
U	eV	Internal energy at 298.15 K
H	eV	Enthalpy at 298.15 K
G	eV	Free energy at 298.15 K

Suppl. Note 12. Attention-based interpretation

We refer to the gradient self-attention maps method for interpreting the DGT model[9]. This method is capable of yielding ad-hoc explanations for attention-based models by deciphering which parts of the input correspond with the predictions. The importance value of the target s w.r.t. the node x_i can be given as:

$$r_{x_i} = \frac{1}{MN} \sum_{m=1}^{M} \sum_{j=1}^{N} [H_x^m]_{ij}$$

$$H_x^m = A_x^m \circ \mathrm{ReLU}(G_x^m)$$

where $G_x^m := \frac{\partial s}{\partial A_x^m}$ is the element-wise gradients of s w.r.t. to A_x^m and $\circ$ is the Hadamard product. m denotes the index of attention head, j the index of node, M the number of attention heads, and N the number of nodes.

Supplementary References

1. Park, W., Chang, W.-G., Lee, D., Kim, J. & hwang, seung-won. GRPE: Relative Positional Encoding for Graph Transformer. in *ICLR2022 Machine Learning for Drug Discovery* (2022).
2. Zhu, J. *et al.* O-GNN: incorporating ring priors into molecular modeling. in *The Eleventh International Conference on Learning Representations* (2023).
3. Ying, C. *et al.* Do transformers really perform badly for graph representation? *Adv. Neural Inf. Process. Syst.* **34**, 28877–28888 (2021).
4. Menegaux, R., Jehanno, E., Selosse, M. & Mairal, J. Self-Attention in Colors: Another Take on Encoding Graph Structure in Transformers. *Trans. Mach. Learn. Res.* (2023).
5. Gasteiger, J., Yeshwanth, C. & Günnemann, S. Directional message passing on molecular graphs via synthetic coordinates. *Adv. Neural Inf. Process. Syst.* **34**, 15421–15433 (2021).
6. Wu, Z. *et al.* MoleculeNet: a benchmark for molecular machine learning. *Chem. Sci.* **9**, 513–530 (2018).
7. Hu, W. *et al.* OGB-LSC: A Large-Scale Challenge for Machine Learning on Graphs. Preprint at https://arxiv.org/abs/2103.09430 (2021).
8. Adams, K., Pattanaik, L. & Coley, C. W. Learning 3D Representations of Molecular Chirality with Invariance to Bond Rotations. Preprint at https://doi.org/arXiv.2110.04383 (2021).
9. Barkan, O. *et al.* Grad-SAM: Explaining Transformers via Gradient Self-Attention Maps. in *Proceedings of the 30th ACM International Conference on Information & Knowledge Management* 2882–2887 (2021).
