# Supplementary material for: Enhancing molecular property prediction of transformer models with dual graph representation
Source: Nat Commun. 2026 Jun 27;17:8039. doi: 10.1038/s41467-026-75005-9 (PMC13454579; doi:10.1038/s41467-026-75005-9)
Supplement: Supplementary file 2 [file 41467_2026_75005_MOESM2_ESM.pdf]

nature portfolio

Peer Review File

Enhancing molecular property prediction of transformer models with dual graph representation

Corresponding Author: Dr Shuyuan Zhang

This file contains all reviewer reports in order by version, followed by all author rebuttals in order by version.

Version 0:

Reviewer comments:

Reviewer #1

(Remarks to the Author)
This work presents the dual graph transformer (DGT) for molecular property prediction, integrating atom and bond graphs with 3D information and graph-specific encodings. The benchmark results across multiple datasets are promising, and the attention-based interpretability is a notable strength. However, several issues need to be addressed to enhance the study's rigor and comprehensiveness.
First, the current DGT design fails to distinguish chiral isomers, as its 3D information (bond lengths, interatomic distances, bond angles) and rotation-invariant encodings cannot capture the absolute spatial configuration differences of enantiomers. The authors should adopt rotation-sensitive 3D encodings (e.g., direction vector-based geometric encodings or chirality-specific basis functions) and design chirality-related pretraining tasks (e.g., enantiomer recognition) using chiral molecule subsets (e.g., from ChEMBL).
Second, benchmark comparisons lack detailed subtask breakdowns for datasets like QM9 (12 regression tasks covering basic physical and electronic/energy properties) and physiology/biophysics datasets. These properties vary in structural sensitivity, and a detailed breakdown would clarify DGT's generalized advantages or potential biases (e.g., strong enthalpy prediction vs. insufficient polarizability accuracy).
Third, the dual graph representation increases model complexity, but trainable parameter counts of DGT and all baselines are unreported—critical to rule out "parameter scaling dividends" (e.g., as in GROVER). Additionally, mixed comparisons of pretrained/non-pretrained baselines confound structural innovations with pretraining gains; benchmarks should be split into "non-pretrained" and "pretrained" groups for fair comparison. Furthermore, the attention-based interpretability should be validated with experimental evidence or chemical principles for more datasets to strengthen credibility. Computational efficiency (training/inference time, memory usage) relative to baselines should be reported for practical high-throughput screening, and limitations in handling large molecules (beyond QM9's 9 heavy atoms) should be discussed.
Addressing these points will improve the work's scientific rigor and practical value, and I recommend revision for publication.

(Remarks on code availability)

Reviewer #2

(Remarks to the Author)

Zhang et al. present a new architecture that advances molecular property prediction through a dual-graph transformer (DGT) with integrated structural and spatial encodings. The major novelty of the architecture is that it integrates the atom graphs and the bond (line) graphs directly into the self-attention mechanism. Through the mechanism, the framework fuses multiple molecular information for the property prediction. The advantages of the framework is further demonstrated via comprehensive benchmark results and the attention-based interpretability across atoms and bonds. Overall, there are some novelties of the study, but there are some key issues.

1) In the abstract, the authors state that "DGT facilitates the fusion of atom and bond features, graph topology and structure, and three-dimensional information within the self-attention module to learn an effective molecular representation", but from Figure 1 and the method, I did not see there is a real fusion here. it is at most a loose and indirect fusion. Both atom and bond features are neither jointly represented nor fused into a shared embedding space.

2) The current description of DGT lacks sufficient mathematical detail. Key components of the attention mechanism and feature fusion are not fully specified, which hinders a clear understanding of the model architecture.

3) As a new molecular representation approach, I think the authors need to evaluate the generalization of DGT to unseen and out-of-distribution molecular datasets. Especially, MoleculeNet benchmark datasets have a number of major flaws (doi: 10.1038/s43588-024-00699-0, 10.1038/s42256-024-00856-0, 10.1038/s41467-023-41948-6).

4) Since the authors have run the analyses 4 times, the result plots should add the error bars.

5). For the ablation analysis and the 3D information incorporation in Figure 3, since the scale is too small, it is very hard to see whether there are any difference or significantly different, I would suggest to repeat the analyses for classification data sets.

(Remarks on code availability)

Reviewer #3

(Remarks to the Author)

The manuscript is well-structured and clearly presented. The authors propose the dual graph transformer (DGT) model, which effectively integrates atom and bond representations to enhance molecular property predictions. They provide a thorough benchmarking of DGT across multiple datasets, demonstrating its superior performance compared to existing state-of-the-art models.
However, there are several critical weaknesses that need to be addressed.

1. Limited Interaction Representation: The manuscript concludes with a simple concatenation of atom representation and bond representation. As shown in Figure 1, this approach fails to effectively capture the intricate interactions between atoms and bonds within the molecular structure. Recent studies, such as those utilizing attention-based mechanisms or message-passing frameworks, have demonstrated that explicitly modeling the interactions between different types of nodes can significantly enhance the predictive power of graph neural networks (GNNs). The lack of a mechanism to model these interactions limits the model's ability to fully leverage the complementary information provided by both representations.
2. Insufficient Utilization of Structural and Spatial Information: The incorporation of structural and spatial information in the model appears to be minimal, as it is treated merely as input features with relatively low significance in the overall architecture. To enhance the predictive capability of the DGT model, I suggest that the authors consider employing advanced techniques, such as 3D equivariant graph neural networks (e.g., SchNet and DimeNet), which can better capture the geometric and spatial relationships inherent in molecular structures. These methods have shown promise in leveraging 3D spatial information to improve predictions of molecular properties, thus providing a more robust representation of molecular behavior.
3. Inadequate Discussion of Encoding Methods: Although the manuscript briefly introduces various encoding methods, such as relative positional encoding (RPE) and ring structural encoding (RSE), it lacks a comprehensive discussion on the limitations of existing methods for atom and edge representations. For instance, while RPE and RSE are valuable, they may not fully capture the complex dependencies that exist within molecular graphs. Additionally, the manuscript does not adequately address how edge features are utilized in current implementations, particularly regarding their aggregation and contribution to updating node features prior to attention calculations. A more thorough examination of these aspects would provide clearer insights into the effectiveness of the proposed dual graph representation.

(Remarks on code availability)

Version 1:

Reviewer comments:

Reviewer #1

(Remarks to the Author)
The authors have made meaningful improvements in response to my comments. The updated model now includes stereochemical descriptors (chiral volume for R/S and an E/Z-related bond encoding), and the additional ablations on stereoselective tasks help support the claims. The added reporting of parameter counts/FLOPs is also appreciated.
I recommend minor revision, mainly to strengthen the credibility and reproducibility of the new R/S chirality benchmark result (reported extremely high accuracy). Please clarify the splitting protocol (2D-graph vs stereoisomer vs conformer level), whether any conformers/stereoisomers from the same 2D graph can appear in different splits, and any de-duplication performed. Ideally, please also provide either (i) results under a strict 2D-graph-level split, or (ii) quantitative overlap statistics demonstrating no cross-split duplicates, and report an additional metric such as balanced accuracy/AUROC (mean±std over runs).
With these clarifications, the revision should be suitable for acceptance.

(Remarks on code availability)

Reviewer #2

(Remarks to the Author)
Thanks for the authors to make the detailed revisions.
Most of my comments have been addressed. I just have below two minors:

1. In Fig. 2b, the physical chemistry domain reports averaged RMSE values. However, in Supplementary Fig. S6, the metric for FreeSolv appears to be MAE rather than RMSE. It may be helpful for the authors to clarify this for consistency.
2. In the physiology domain, the major performance gain appears to come from the SIDER dataset (Fig. S4), where the improvement is particularly significant. It would be interesting if the authors could further discuss why the proposed method is especially effective on this dataset, and whether this may relate to the dual-graph representation or the incorporation of structural/stereochemical information.

(Remarks on code availability)

Reviewer #3

(Remarks to the Author)
I appreciate the authors' careful and comprehensive responses to my previous comments. The revised manuscript has adequately addressed my concerns, and the additional analyses and textual revisions have strengthened the study. I have no further major comments and support publication of the manuscript.

(Remarks on code availability)
The code is well organized, clearly documented, and easy to follow. I have examined the accompanying code and found it to be of high quality. It supports the analyses presented in the manuscript.

Version 2:

Reviewer comments:

Reviewer #1

(Remarks to the Author)
I find the responses satisfactory and have no further comments or requested revisions.

(Remarks on code availability)

Reviewer #2

(Remarks to the Author)
My comments have been addressed. I have no other comments.

(Remarks on code availability)

Open Access This Peer Review File is licensed under a Creative Commons Attribution 4.0 International License, which permits use, sharing, adaptation, distribution and reproduction in any medium or format, as long as you give appropriate credit to the original author(s) and the source, provide a link to the Creative Commons license, and indicate if changes were made.
In cases where reviewers are anonymous, credit should be given to 'Anonymous Referee' and the source.
The images or other third party material in this Peer Review File are included in the article's Creative Commons license, unless indicated otherwise in a credit line to the material. If material is not included in the article's Creative Commons license and your intended use is not permitted by statutory regulation or exceeds the permitted use, you will need to obtain permission directly from the copyright holder.
To view a copy of this license, visit https://creativecommons.org/licenses/by/4.0/

Authors' Response to Reviews of

Enhancing molecular property prediction via transformer with dual graph representation

Shuyuan Zhang, Alexei A. Lapkin
Nature Communications, *E-mail: sz469@cam.ac.uk*

RC: ***Reviewers' Comment***, AR: Authors' Response, □ Manuscript Text

We appreciate the editor and reviewers for their valuable feedback and suggestions. We believe that we have addressed all comments in this point-by-point response and made the requisite revisions to our manuscript accordingly, which have significantly strengthened this work. All relevant changes to the manuscript text are reproduced in this response, with newly added text highlighted in blue. Reviewers' comments are also reproduced in full below.

Reviewer 1

RC: ***This work presents the dual graph transformer (DGT) for molecular property prediction, integrating atom and bond graphs with 3D information and graph-specific encodings. The benchmark results across multiple datasets are promising, and the attention-based interpretability is a notable strength. However, several issues need to be addressed to enhance the study's rigor and comprehensiveness.***

AR: Thank you for the overall positive assessment of our work. We believe that this DGT represents a forward step in molecular machine learning. We hope our responses and revisions to DGT outlined below will improve the scientific rigor and practical value of this work.

RC: ***First, the current DGT design fails to distinguish chiral isomers, as its 3D information (bond lengths, interatomic distances, bond angles) and rotation-invariant encodings cannot capture the absolute spatial configuration differences of enantiomers. The authors should adopt rotation-sensitive 3D encodings (e.g., direction vector-based geometric encodings or chirality-specific basis functions) and design chirality-related pretraining tasks (e.g., enantiomer recognition) using chiral molecule subsets (e.g., from ChEMBL).***

AR: We appreciate this insightful comment regarding the model's ability in recognising enantiomers. To address your concern, we have added a chirality-specific node–node encoding and a *E/Z* isomerism-specific bond–bond encoding to DGT. The renewed DGT illustration (Fig. 1) is reproduced here as Fig. R1, where atom and bond encodings are fused before graph transformer layers.

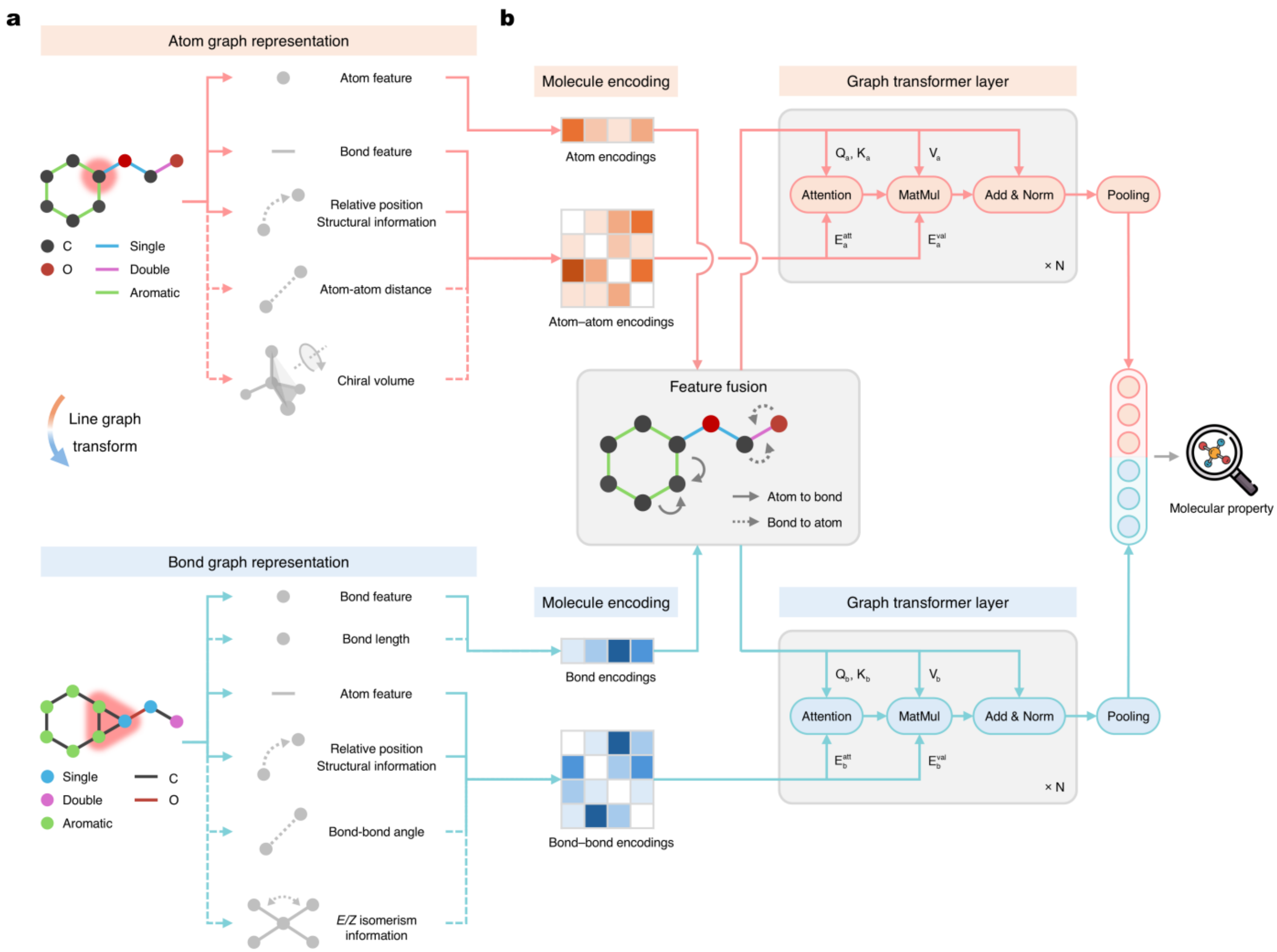

Fig. R1: Reproduced Fig. 1, an illustration of the Dual Graph Transformer (DGT). **a**, Atom and bond graph representations of molecules. Positions of atoms and bonds are exchanged in the dual graph representation, accommodating atom and bond features, relative position, and structural information. For instance, the highlighted *ipso* carbon in the atom graph corresponds to the three highlighted edges in the corresponding bond graph. Bond length, atom–atom distance, and bond–bond angle can also be integrated into DGT as geometric information when available. DGT is able to incorporate chiral volume and *E/Z* isomerism information to enhance performances on stereoselective tasks. **b**, Molecule encodings, feature fusion, and graph transformer structure. DGT encodes molecules into atom and bond vectors, atom–atom matrices, and bond–bond matrices. Atom and bond encodings are fused before the query-key-value operation by stacked graph transformer layers, followed by the pooling layer for predicting molecular properties.

For a chiral atom, the chiral volume of a directed bond is calculated as the signed volume of the other three directed bonds arranged using the Cahn-Ingold-Prelog priority rules[1]. Given vectors of those three ordered directed bonds as $\boldsymbol{v}_1$, $\boldsymbol{v}_2$ and $\boldsymbol{v}_3$, the signed chiral volume is calculated as $(\boldsymbol{v}_1 \times \boldsymbol{v}_2) \cdot \boldsymbol{v}_3$, which is dependent on chirality and invariant to bond rotations[2]. Therefore, DGT is endowed with the ability to distinguish configuration differences of enantiomers. We benchmarked DGT incorporating chiral volume, hereafter denoted as DGT(R/S), on a published subset of PubChem3D for *R/S* classification (Fig. R2, now

included in Supplementary Information as Fig. S9). The dataset consists of 2.98M conformers of 598K stereoisomers of 257K 2D graphs[3]. DGT(R/S) delivers a high *R/S* classification accuracy of 99.99%, outperforming other reported models under the same data splitting practice.

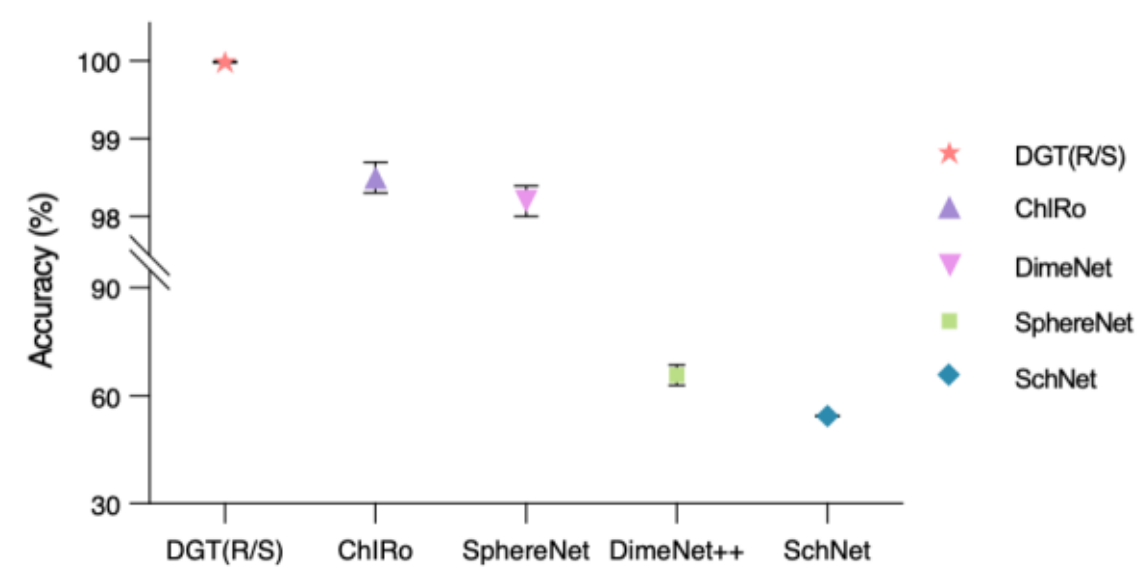

Fig. R2: Performance benchmark of DGT(R/S) for chiral classification on the PubChem3D subset against ChIRo, DimeNet, SphereNet and SchNet. Mean DGT(R/S) performance is derived from four independent experimental runs. Performance metrics of other models are adapted from ref. 3.

To evaluate the specific contributions of stereochemical encodings, we then conducted ablation analysis on the Tox21 Aromatase and Estrogen Receptor (ER) classification tasks. These targets are selected because their biological activity is highly sensitive to the steroidal scaffold, where both *R/S* chirality and *E/Z* isomerism have a prominent impact[4–6]. As hypothesised, adding stereochemical information to DGT improved its classification performance (Fig. R3). AUC-ROC was increased by about 1.5% for both tasks after incorporating the *E/Z* isomerism information into the bond–bond encoding (DGT(E/Z)). This performance was enhanced in DGT(R/S+E/Z), which, compared to DGT(E/Z), further incorporates chiral volume into the atom–atom encodings to resolve enantiomeric ambiguity. Furthermore, leveraging a pretraining strategy on the PubChem3D subset for enantiomer recognition, as your suggestion, resulted in an approximate 1.4% gain for both tasks.

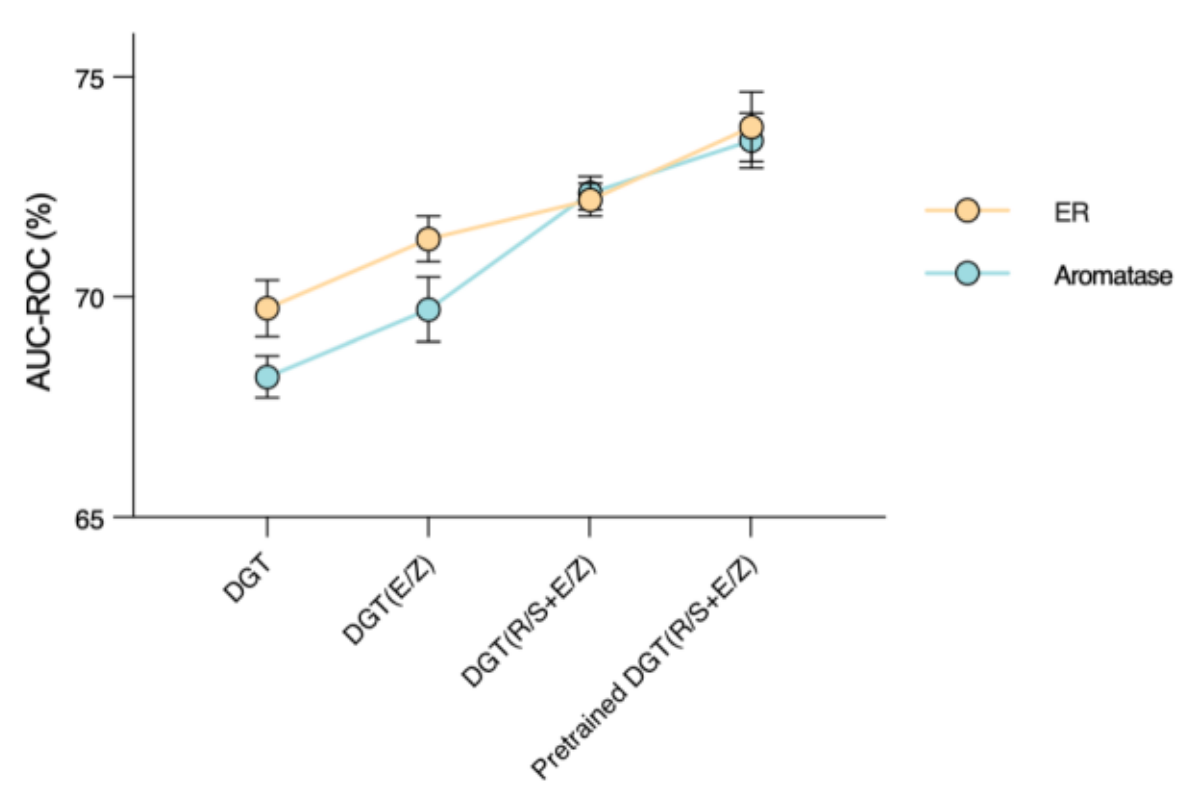

Fig. R3: Ablation analysis of DGT incorporating *R/S* and *E/Z* isomerisms on the Tox21 Aromatase and Estrogen Receptor (ER) classification tasks. Pretraining is conducted on the PubChem3D subset for enantiomer recognition. All performance results are averaged over four independent runs.

In response to the reviewer's comment, we have revised our manuscript accordingly to reflect the modified model structure (Fig. 1). Since chirality and *E/Z* isomerism are features primarily relevant to specific stereochemical prediction tasks (such as those involving protein-ligand interactions), these two encodings are evaluated in Fig. 3e for the Tox21 Aromatase and ER classification tasks but not included in the DGT model for the general benchmark in Fig 2. Other subfigures in Fig. 3 have been also updated to align with the model structure modification.

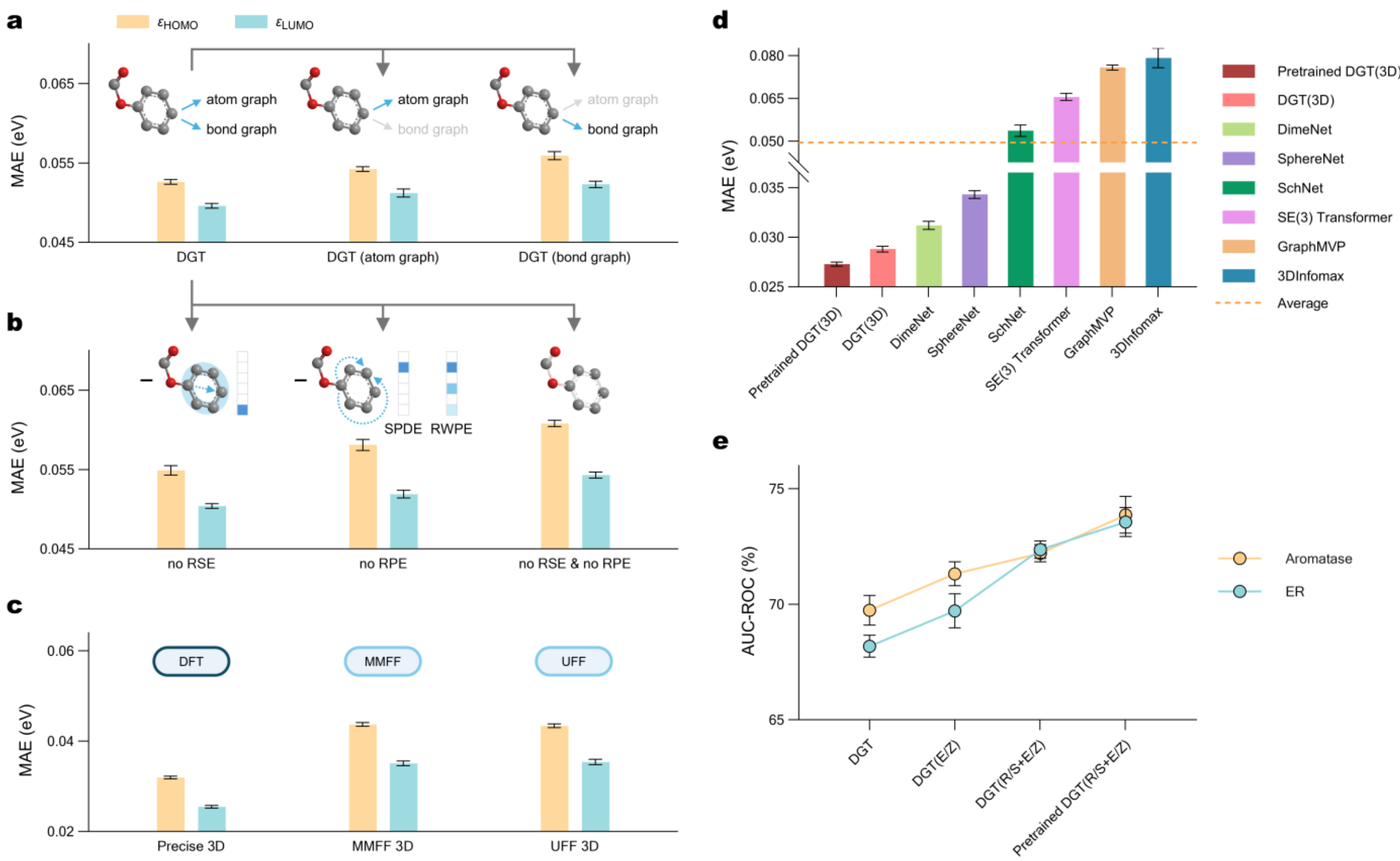

Fig. R4: Reproduced Fig. 3, performance investigation of DGT and stereogeometric information incorporation. The efficacy of incorporating 3D information was evaluated through HOMO and LUMO energy predictions on the QM9 dataset, while the impact of R/S chirality and E/Z isomerism was validated using the Tox21 Aromatase and Estrogen Receptor (ER) classification tasks. **a**, Ablation study evaluating the dual graph representation. **b**, Ablation study of graph-specific encodings. **c**, Effect of incorporating 3D information at different fidelity levels on DGT's predictive performance. **d**, Comparative assessment of the 3D information-augmented DGT, denoted as DGT(3D), against state-of-the-art models for 3D molecular learning using the average prediction MAE of HOMO and LUMO energies. The pretraining of DGT(3D) is conducted on molecules from the PCQM4Mv2 dataset targeting the HOMO–LUMO energy gap. **e**, Ablation study evaluating the contribution of *R/S* chirality and *E/Z* isomerism to DGT performance. A reported PubChem3D subset for enantiomer recognition[3] is adopted for pretraining.

We have updated the overview Section to introduce the integration of chiral volume and *E/Z* isomerism information to DGT.

Additionally, this dual graph representation accommodates diverse stereogeometric information: Bond lengths can be embedded within bond features; atom–atom distances and bond–bond angles should fit into the pairwise feature matrices of atom and bond graphs, respectively; chiral volume can be captured as a stereochemical descriptor in the atom–atom encodings; relationships of bonds in *E/Z* isomers align with the dimensions of bond–bond encodings. To smoothen the continuous geometric information of bond–bond angles, the normalised spherical harmonics are employed to provide an orthonormal representation of angular dependencies (Fig. S2).

The discussion pertaining to Figs. 3d and 3e has been revised as follows:

~~As depicted in Fig. 3d, we meticulously assessed the performance trajectory of DGT(3D) when pretrained on subsets of the PCQM4Mv2 dataset of increasing size (0, 10K, 100K, and 1M molecules) for predicting $\varepsilon_{\mathrm{HOMO}}$ and $\varepsilon_{\mathrm{LUMO}}$. Enlarging the pretraining dataset delivered a monotonic reduction in MAE, underscoring the profound benefit of large-scale pretraining in facilitating DGT(3D) to learn a more generalised molecular representation for relevant downstream tasks. Quantitatively, the ultimate prediction MAEs for $\varepsilon_{\mathrm{HOMO}}$ and $\varepsilon_{\mathrm{LUMO}}$ were as low as 0.0302 eV and 0.0231 eV, respectively, corresponding to considerate improvements of 9.3% and 7.2% attributable to pretraining.~~

Fig. 3~~e~~d compares DGT(3D) against some established state-of-the-art models designed for 3D molecular learning. The benchmark models included SchNet, DimeNet, SphereNet, SE(3) Transformer, GraphMVP, and 3DInfomax. SchNet, DimeNet, and SphereNet represented 3D GNNs that use rotationally invariant features to predict scalar molecular properties. The SE(3) Transformer was included as a typical equivariant GNN that is robust to 3D roto-translations of molecules. GraphMVP and 3DInfomax were models adopting contrastive learning to enrich 2D graph structure embeddings with 3D conformational information. DGT(3D), whether pretrained or not, outperformed all other evaluated models, firmly establishing it as a highly effective 3D machine learning architecture for molecular property prediction. When pretrained on 10K random molecules sampled from the PCQM4Mv2 dataset, DGT(3D) attained prediction MAEs of 0.0306 eV for $\varepsilon_{\mathrm{HOMO}}$ and and 0.0240 eV for $\varepsilon_{\mathrm{LUMO}}$, though no significant correlation was observed between pretraining dataset size and downstream prediction accuracy (Fig. S9).

The efficacy of integrating chiral volume and *E/Z* isomerism with DGT was validated on the Tox21 Aromatase and ER tasks, both of which exhibit high stereoselectivity due to their steroidal-binding motifs. As hypothesised, the inclusion of stereochemical descriptors consistently improved the classification performance (Fig. 3e). Incorporating *E/Z* isomerism information into the bond–bond encodings (DGT(E/Z)) yielded an approximate 1.5% increase in AUC-ROC across both tasks. This performance was further enhanced in DGT(R/S+E/Z), which incorporates chiral volume into the atom–atom encodings to resolve enantiomeric ambiguity. Based on this architecture, a pretraining strategy was leveraged using a PubChem3D subset, where DGT outperformed other stereochemical models in enantiomer recognition (Fig. S10). This specific pretraining stage facilitated an additional gain of about 1.4% in AUC-ROC for both Tox21 tasks.

RC: ***Second, benchmark comparisons lack detailed subtask breakdowns for datasets like QM9 (12 regression tasks covering basic physical and electronic/energy properties) and physiology/biophysics datasets. These properties vary in structural sensitivity, and a detailed breakdown would clarify DGT's***

generalized advantages or potential biases (e.g., strong enthalpy prediction vs. insufficient polarizability accuracy).

AR: Thank you for pointing this out. The breakdown performance of all tasks across four domains of the MoleculeNet dataset is now detailed in Figs. S4–S7. No particular subtask biases can be observed for DGT, showing its general performance advantage over other models. The performance results on subtasks of QM9 are reproduced here as Fig. R5 as an example.

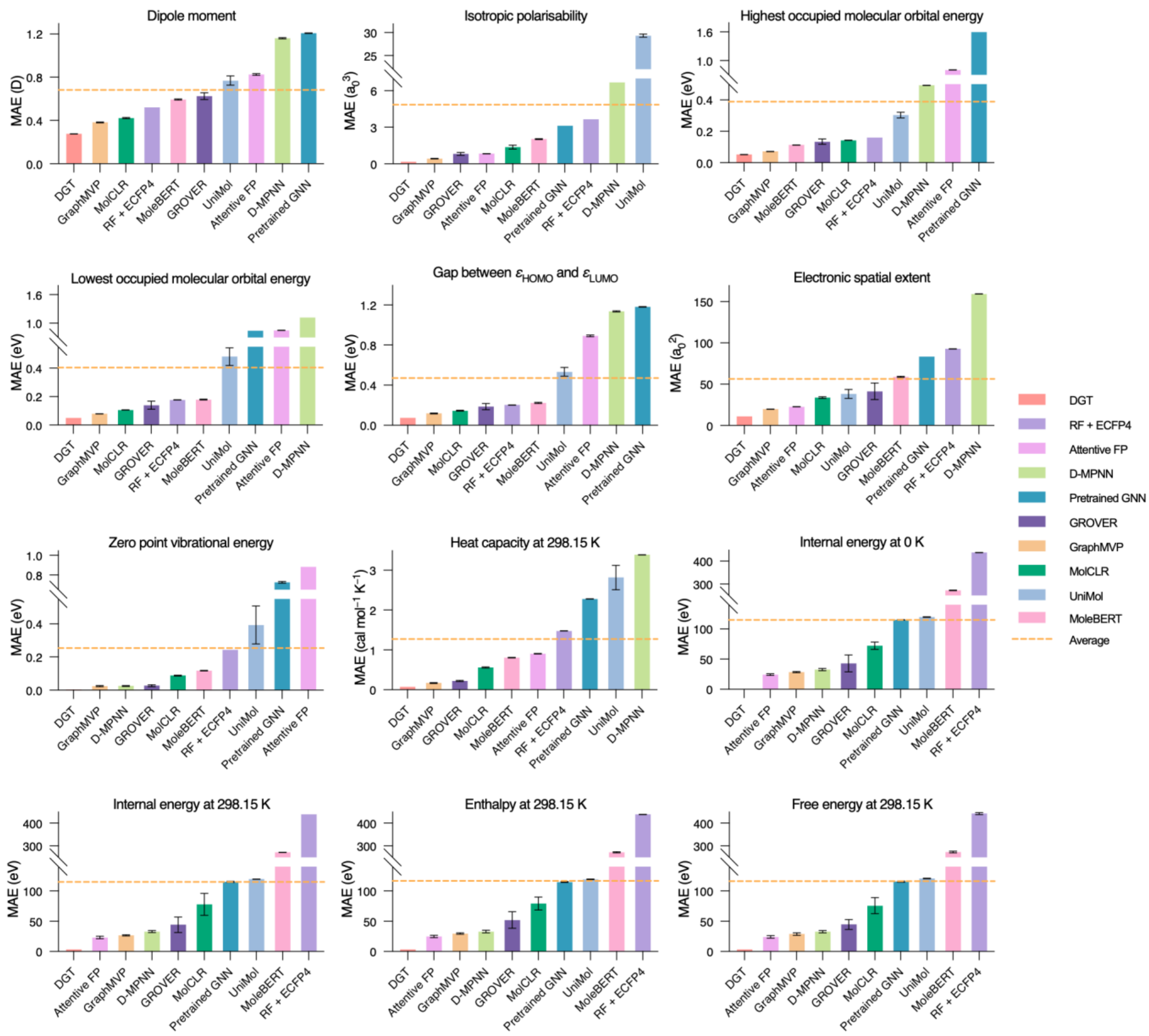

Fig. R5: Reproduced Fig. S7, detailed Performance comparison of DGT against nine baselines on the 12 tasks of the QM9 dataset within the quantum mechanics domain. DGT achieves the best performance on all tasks.

These detailed benchmark figures are now referenced in the revised manuscript to clarify DGT's performance on breakdown subtasks.

> Model performance breakdowns for subtasks in the physiology and biophysics domains are detailed in Figs. S4 and S5. These collective results affirm DGT's broad applicability and high accuracy in predicting molecular properties relevant to drug discovery, including pharmacokinetics, toxicity, bioactivity, and potential adverse effects.
>
> …
>
> Figs. S6 and S7 display model performance breakdowns for subtasks of physical chemistry and quantum mechanics domains, respectively. These results unveil the exceptional ability of DGT to precisely predict numerical physicochemical and quantum mechanical properties for molecules.

RC: ***Third, the dual graph representation increases model complexity, but trainable parameter counts of DGT and all baselines are unreported—critical to rule out "parameter scaling dividends" (e.g., as in GROVER).***

AR: We thank the reviewer for the suggestion to provide details about model complexity. Table R1 (now included in the Supplementary Information as Table S5) details the parameter counts and floating-point operations (FLOPs) for models evaluated in this work. Notably, our proposed DGT does not exhibit a significantly larger parameter amount than its counterparts. As a comparison, even though the GROVER model utilises ~ 100M parameters and requires 1G FLOPs for inference on methane (the first entry of the QM9 dataset), it underperforms DGT across all four MoleculeNet domains. These results indicate that DGT's performance is derived from model architecture rather than mere parameter scaling. We have updated the Results Section to explicitly discuss "parameter scaling dividends" and the computational efficiency of our approach.

Table R1: Summary of model parameter amounts and floating-point operations (FLOPs) during inference. The value of FLOPs is benchmarked on methane, which is the first molecule of the QM9 dataset.

Model	Parameter amount	FLOPs
DGT	2.9M	28M
RF + ECFP4	13.3M	0.02M
Attentive FP	0.2M	2M
D-MPNN	0.4M	6M
Pretrained GNN	1.9M	25M
GROVER	~100M	~1G
GraphMVP	5.8M	6M
MolCLR	1.0M	5M
UniMol	~90M	~140M
MoleBERT	1.8M	5M
DimeNet	1.8M	2M
SphereNet	1.8M	3M
SchNet	1.6M	8M
SE(3) Transformer	1.4M	9M
3DInfomax	2.1M	14M

Note: Parameters and FLOPs for DGT are based on the QM9 model configuration. The computational overhead of DGT(3D) is close to DGT due to their identical core structure of stacked graph transformer

layers.

> These results highlight the potential of leveraging stereogeometric information in the dual graph representation to enhance the self-attention mechanism in molecular machine learning. Furthermore, model parameter amount and floating-point operations during inference are summarised in Table S5, demonstrating that DGT's performance stems from the dual graph representation rather than parameter scaling.

RC: ***Additionally, mixed comparisons of pretrained/non-pretrained baselines confound structural innovations with pretraining gains; benchmarks should be split into "non-pretrained" and "pretrained" groups for fair comparison.***

AR: We appreciate the reviewer's thoughtful suggestion regarding the categorization of model performance. We agree that distinguishing between "non-pretrained" and "pretrained" models is a valuable perspective for understanding the origins of performance gains.

However, because baseline models included in this work adopt different architectures and training strategies, isolating the quantitative contribution of pretraining or other factors is a significant challenge. Furthermore, our intention here is to benchmark the intrinsic performance of DGT in molecular machine learning, and the results reveal that DGT achieves state-of-the-art results even without pretraining. As a comparison, GraphMVP, despite being pretrained on 50k qualified molecules from GEOM, only delivers the second-best performance in quantum mechanics and the average performance across the other three domains in the MoleculeNet benchmark.

To preserve the comparative clarity of the overall benchmark while addressing the reviewer's concern, we cautiously opted to maintain the current format of Fig. 2 but have added a sentence to the Results section to clarify the role of pretraining in the benchmark. This additional sentence underscores DGT's molecular learning ability even without pretraining, showing that DGT's performance is driven by its dual graph representation for molecules.

> The graph transformer layer addresses this by enabling nodes to aggregate information directly from the entire graph, rather than being restricted to their *k*-hop neighbourhoods, while embedding graph-specific encodings into the attention score matrix, including topological and structural information. Furthermore, the DGT's outstanding performance is underscored by its ability to outperform baselines with pretraining, such as GraphMVP, despite their extensive molecular data exposure.

RC: ***Furthermore, the attention-based interpretability should be validated with experimental evidence or chemical principles for more datasets to strengthen credibility.***

AR: Thanks for pointing out the need to strengthen the credibility of our attention-based interpretability. Following the complementation of chirality and *E/R* isomerism encodings in DGT, we have incorporated fumagillin and tamoxifen in Fig. 4 as representative stereoisomeric inhibitors for the Tox21 Aromatase and ER classification assays, respectively.

The revised Fig. 4 is reproduced here as Fig. R6. For fumagillin, the attention is primarily localized on the spiro-oxirane moiety and the chiral centres of the cyclohexane ring. This attention distribution aligns with the established inhibitory mechanism[7]: the highly strained and electrophilic spiro-oxirane acts as a reactive site for irreversible covalent bonding with a histidine residue in the enzyme pocket. Meanwhile, the 1,4-trans-configuration between C3 oxygen and C6 side chain is structurally critical to ensure the precise spatial orientation of the spiro-oxirane toward the active site residues.

In the case of tamoxifen, the normalized attention scores highlight the dimethylaminoethoxy side chain and the triphenylethylene core. Upon binding to the ER, the triphenylethylene core serves as a structural anchor to occupy the hydrophobic binding pocket, while the bulky, basic dimethylaminoethoxy chain protrudes out of the binding pocket, preventing Helix 12 from folding over the pocket[8]. Notably, the *E*-isomer (where the two phenyl rings without a dimethylaminoethoxy chain are *cis*-positioned) exhibits significantly lower affinity for the ER[4].

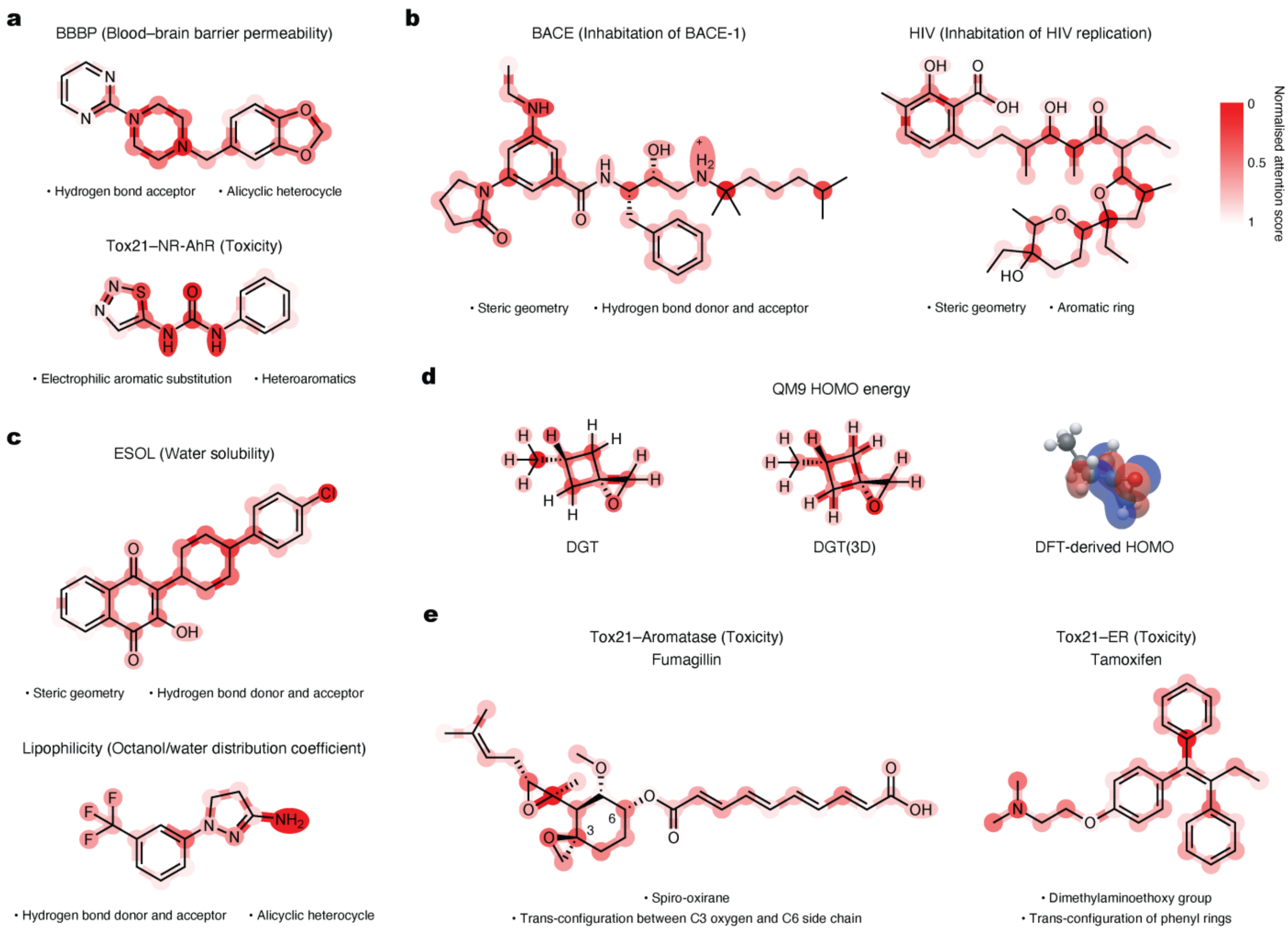

Fig. R6: Reproduced Fig. 4, attention-based interpretation analysis results. a–d, Attention-based interpretation is conducted on molecular property prediction tasks across domains of physiology (a), biophysics (b), physical chemistry (c), and quantum mechanics (d). Attention scores calculation concurrently considers the attention score matrices of atoms and bonds, as well as the absolute gradients of the prediction with respect to them. Darker colours denote a higher level of attention score assigned to

the atom or bond. Attention scores calculated using DGT(3D) are provided for the QM9 HOMO energy prediction example, along with the DFT-derived HOMO at the B3LYP/6-31G(2df,p) level of theory. e, Attention-based stereochemical interpretability analysis using DGT with chirality and *E/Z* isomerism encodings incorporated. Fumagillin and tamoxifen are presented as representative stereoisomeric inhibitors for the Tox21 Aromatase and ER assays, respectively.

By incorporating these two stereoselective inhibitors with reported mechanisms, we hope the revised manuscript have strengthened the credibility for the attention-based interpretability. Explanations for fumagillin and tamoxifen have been included in our revised manuscript.

> Compared to DGT, DGT(3D) performs better to distribute greater attention to the epoxide and cyclobutane rings in the illustrated molecule, which is corroborated by the DFT-derived HOMO (Fig. 4d).
>
> Fumagillin and tamoxifen are incorporated in Fig. 4e as representative stereoisomeric inhibitors for the Tox21 Aromatase and ER classification assays, respectively. For fumagillin, the attention is primarily localised on the spiro-oxirane moiety and the chiral centres of the cyclohexane ring. This attention distribution aligns with the established inhibitory mechanism: the highly strained and electrophilic spiro-oxirane acts as a reactive site for irreversible covalent bonding with a histidine residue in the enzyme pocket. Meanwhile, the 1,4-*trans*-configuration between C3 oxygen and C6 side chain is structurally critical to ensure the precise spatial orientation of the spiro-oxirane toward the active site residues. In the case of tamoxifen, the normalised attention scores highlight the dimethylaminoethoxy side chain and the triphenylethylene core. Upon binding to the ER, the triphenylethylene core serves as a structural anchor to occupy the hydrophobic binding pocket, while the bulky, basic dimethylaminoethoxy chain protrudes out of the binding pocket, preventing Helix 12 from folding over the pocket. Notably, the *E*-isomer (where the phenyl rings are cis-positioned) exhibits significantly lower affinity for the ER.
>
> These findings underscore the potential of DGT to offer valuable insights for molecular property at the molecular structural level.

RC: ***Computational efficiency (training/inference time, memory usage) relative to baselines should be reported for practical high-throughput screening, and limitations in handling large molecules (beyond QM9's 9 heavy atoms) should be discussed.***

AR: We thank the reviewer for highlighting the importance of computational efficiency and the potential limitations concerning molecular scale. In response, we have provided a comparative summary of parameter counts and floating-point operations (FLOPs) for DGT and baseline models in the Supplementary Information (Table S5) as aforementioned.

The analysis indicates that DGT does not significantly exceed the parameterization or FLOP requirements of existing state-of-the-art models. However, we acknowledge that the integration of atom–atom and bond–bond encodings introduces a computational complexity that scales quadratically with the molecular size. For the context of drug discovery, over 90% of marketed drugs are small molecules (typically < 1000 Daltons)[9]; consequently, the proposed DGT remains feasible for most high-throughput screening tasks, as evidenced by its application to the HIV dataset (molecular weights ranging from ~30 to 1,800 Daltons).

To extend DGT's utility to larger molecules in drug discovery or other domains, two primary strategies are proposed: i) On the hardware-level scalability side, implementation of model parallelism across multiple GPUs is considered to be feasible, because all calculations in this work are conducted on a single RTX 4090 GPU card; ii) On the algorithmic optimisation side: the adoption of hierarchical or sparse attention mechanisms to bypass the quadratic scaling bottleneck can be considered[10]. These points have now been included in the discussion part as prospects for DGT.

> DGT also holds the potential to promote more advanced domains such as conformation generation, molecular generative modelling, and chemical reaction prediction. It is also notable that the integration of atom–atom and bond–bond encodings introduces a computational complexity that scales quadratically with the molecular size. Model parallelism and the adoption of hierarchical or sparse attention mechanisms can be considered to extend the DGT's utility on large molecules.

Reviewer 2

RC: ***Zhang et al. present a new architecture that advances molecular property prediction through a dual-graph transformer (DGT) with integrated structural and spatial encodings. The major novelty of the architecture is that it integrates the atom graphs and the bond (line) graphs directly into the self-attention mechanism. Through the mechanism, the framework fuses multiple molecular information for the property prediction. The advantages of the framework is further demonstrated via comprehensive benchmark results and the attention-based interpretability across atoms and bonds. Overall, there are some novelties of the study, but there are some key issues.***

AR: Thank you for the overall positive feedback on our work. We hope our responses and revisions to the manuscript outlined below will further increase the clarity of our work and address the comments raised.

RC: ***1) In the abstract, the authors state that "DGT facilitates the fusion of atom and bond features, graph topology and structure, and three-dimensional information within the self-attention module to learn an effective molecular representation", but from Figure 1 and the method, I did not see there is a real fusion here. it is at most a loose and indirect fusion. Both atom and bond features are neither jointly represented nor fused into a shared embedding space.***

AR: We thank the reviewer for this insightful comment on the model structure design regarding feature fusion. In response to this comment, we have added a feature fusion module before stacked graph transformer layers. The renewed DGT illustration (Fig. 1) is reproduced here as Fig. R7, where atom and bond encodings are fused in the feature fusion module. Chiral volume and *E/Z* isomerism information are also added as optional molecule encodings for stereoselective tasks.

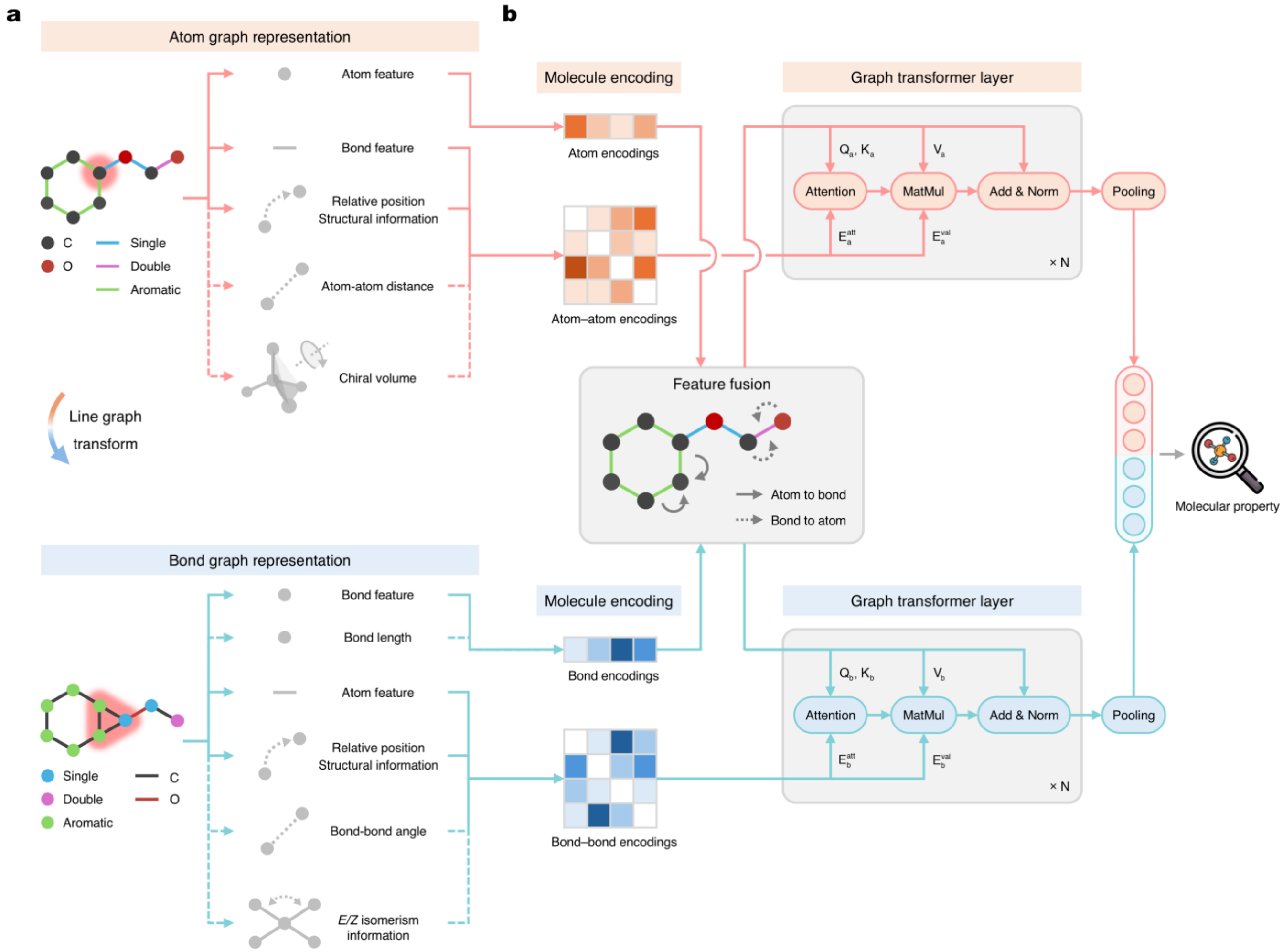

Fig. R7: Reproduced Fig. 1, an illustration of the Dual Graph Transformer (DGT). **a**, Atom and bond graph representations of molecules. Positions of atoms and bonds are exchanged in the dual graph representation, accommodating atom and bond features, relative position, and structural information. For instance, the highlighted *ipso* carbon in the atom graph corresponds to the three highlighted edges in the corresponding bond graph. Bond length, atom–atom distance, and bond–bond angle can also be integrated into DGT as geometric information when available. DGT is able to incorporate chiral volume and *E/Z* isomerism information to enhance performances on stereoselective tasks. **b**, Molecule encodings, feature fusion, and graph transformer structure. DGT encodes molecules into atom and bond vectors, atom–atom matrices, and bond–bond matrices. Atom and bond encodings are fused before the query-key-value operation by stacked graph transformer layers, followed by the pooling layer for predicting molecular properties.

Following the integration of the feature fusion module, we re-evaluated the DGT model (Fig. R8) on the MoleculeNet dataset. The core conclusions of our study remain unchanged, with a slight performance gain observed on the QM9 dataset, as shown in Fig. R9. The atom–bond feature fusion led to a decrease in MAE across 10 of the 12 QM9 subtasks, resulting in an average reduction of approximately 10%.

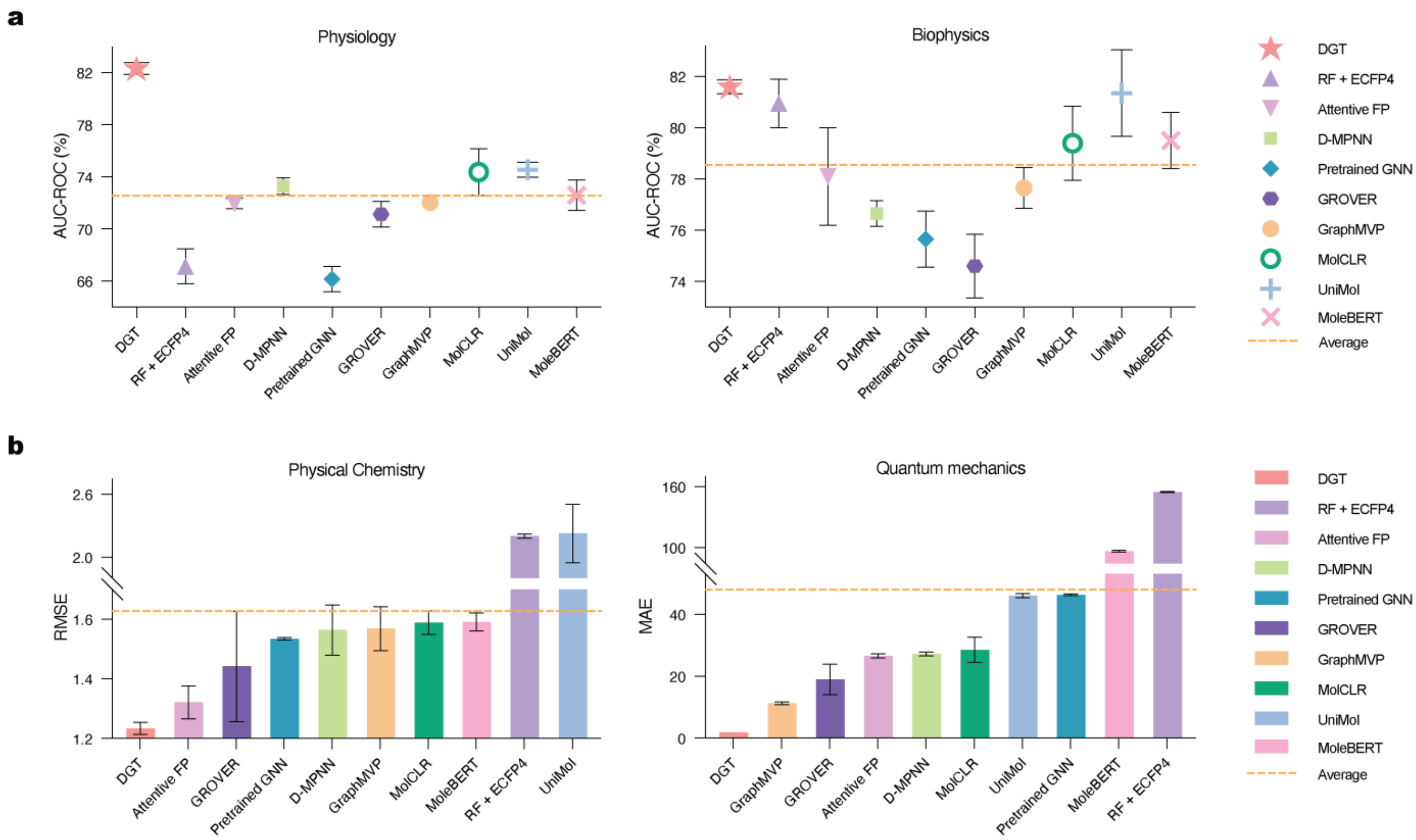

Fig. R8: Reproduced Fig. 2, benchmarking DGT against baselines across four domains. a, Performance comparison of DGT against baselines for predicting categorical molecular properties in physiology and biophysics domains. b, Performance comparison of DGT against baselines for predicting numerical molecular properties in physical chemistry and quantum mechanics domains.

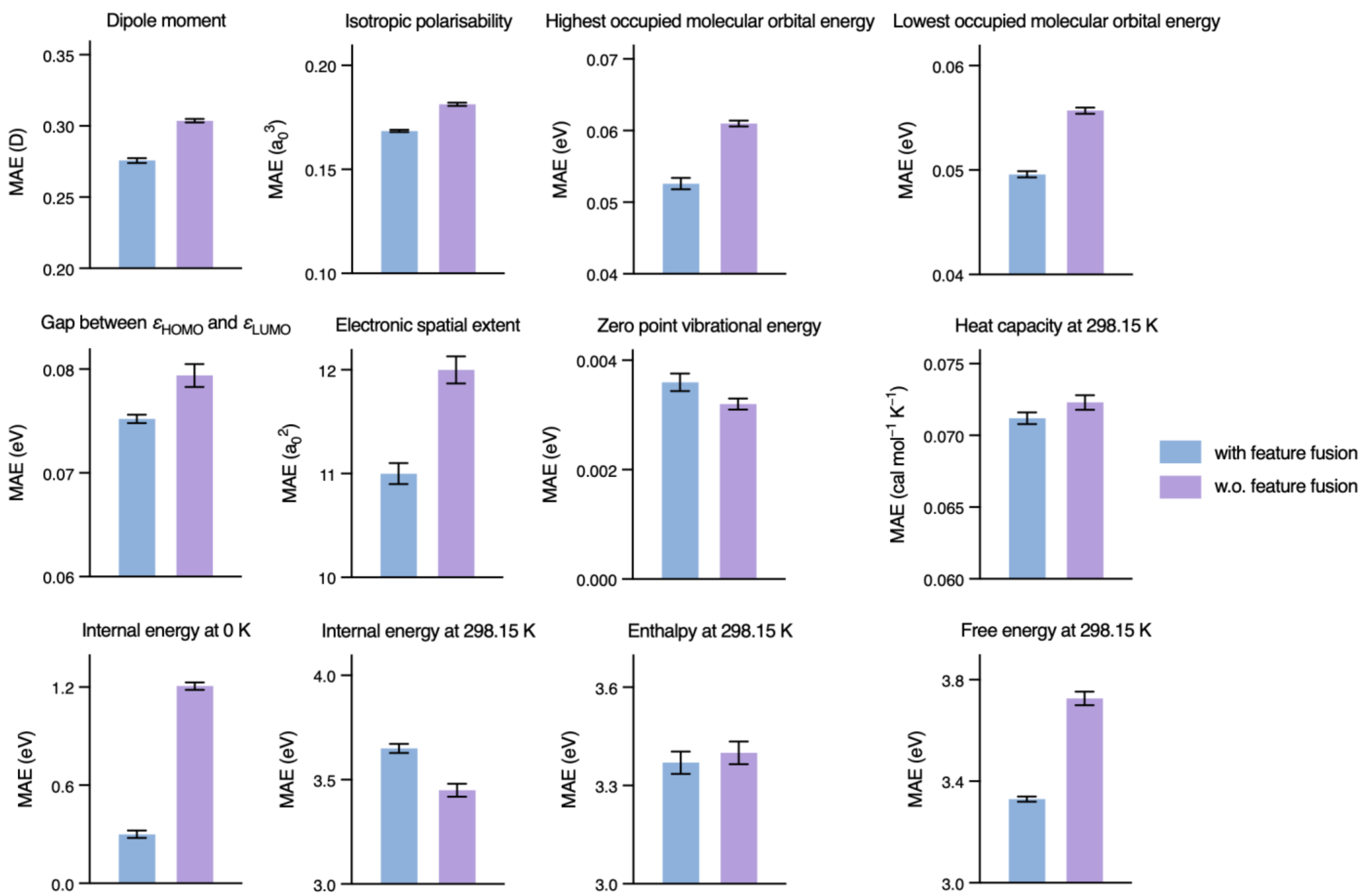

Fig. R9: Performance improvement on the QM9 dataset after introducing the feature fusion module to DGT.

We have revised the DGT overview Section accordingly and updated the results of DGT on the MoleculeNet benchmark in the main text.

Additionally, this dual graph representation accommodates diverse stereogeometric information: Bond lengths are embedded within bond features, while atom–atom distances and bond–bond angles are mapped onto the pairwise feature matrices of atom and bond graphs, respectively. The chiral volume is encoded as a stereochemical descriptor in the atom–atom encodings; relative orientation of vicinal bonds in *E/Z* isomers is integrated into the bond–bond encodings. To smoothen the continuous geometric information of bond–bond angles, the normalised spherical harmonics are employed to provide an orthonormal representation of angular dependencies (Fig. S2).

The feature fusion module positioned upstream stacked graph transformer layers employs a bidirectional aggregation strategy to synthesise an integrated representation: bond embeddings are augmented by concatenating their feature vectors with the mean representation of their incident atoms. Conversely, atomic embeddings are enriched by incorporating the average of their adjacent edge representations (Fig. 1b). The graph transformer layer extends the conventional query-key-value operation by merging the pairwise feature matrices from atom and bond graphs into their respective

attention score matrices and passing messages, allowing DGT to learn both local and global dependencies for atoms and bonds.

RC: ***2) The current description of DGT lacks sufficient mathematical detail. Key components of the attention mechanism and feature fusion are not fully specified, which hinders a clear understanding of the model architecture.***

AR: We apologise for missing mathematical details of the feature fusion and attention mechanism of DGT. As a response, we have supplemented explanations for molecule encoding, feature fusion, and graph transformer layer of DGT in the Methods Section. The Supplementary Information also includes a schematic illustration (Fig. S3) for the graph transformer layer, which is reproduced here as Fig. R10.

Molecule encoding. For the DGT architecture, atom encodings are constructed from a comprehensive suite of categorical descriptors—including atomic number, chirality, formal valency, hybridization state, aromaticity, and a ring membership indicator. Parallel to this, bond encodings of DGT encompass bond order, stereoisomerism type, and a conjunction indicator. DGT can be supplemented with bond lengths, atom–atom distances, and bond–bond angles as 3D molecular descriptors. Additionally, chiral volume and inter-bond *E/Z* isomeric descriptor can be captured as stereogeometric information for DGT.

Specifically, the chiral volume is defined as the scalar triple product of the vectors originating for a directed bond from a stereocentre. To ensure a unique and physically meaningful value, its three neighbouring bonds are ordered as vectors $\boldsymbol{v}_1$, $\boldsymbol{v}_2$ and $\boldsymbol{v}_3$ according to the Cahn-Ingold-Prelog (CIP) rules. The signed chiral volume is then calculated as $(\boldsymbol{v}_1 \times \boldsymbol{v}_2) \cdot \boldsymbol{v}_3$. The inter-bond *E/Z* isomeric descriptor embeds the relative *cis/trans* orientation of vicinal bonds across the double bond into the bond–bond encodings.

Feature fusion. The feature fusion module provides a mutual extension of atom and bond encodings. It concatenates the feature vector of each bond with the mean representations of its incident atoms; likewise, each atomic feature vector is appended with the average of its adjacent bond features. Formally, we define atom and bond graphs as $\mathcal{G}_a = (\mathcal{N}_a, \mathcal{E}_a)$ and $\mathcal{G}_b = (\mathcal{N}_b, \mathcal{E}_b)$, respectively, where $\mathcal{N}_a$ indicates the set of atoms, $\mathcal{E}_a \subseteq \mathcal{N}_a \times \mathcal{N}_a$ the set of directed bonds, $\mathcal{N}_b$ the set of undirected bonds, and $\mathcal{E}_b \subseteq \mathcal{N}_b \times \mathcal{N}_b$ the set of neighbour pairs of undirected bonds. Given the atom feature dimension size as d_a, the atom features are encoded as rows in a matrix $\mathbf{N}_a \in \mathbb{R}^{|\mathcal{N}_a| \times d_a}$; likewise, the bond features are encoded as $\mathbf{N}_b \in \mathbb{R}^{|\mathcal{N}_b| \times d_b}$, and d_b is the bond feature dimension size. The feature fusion operation can be expressed as:

$$\mathbf{N}_{a,i} = \mathbf{N}_{a,i}^0 \parallel \frac{1}{|\mathcal{N}_{b,i}|} \sum_{j \in \mathcal{N}_{b,i}} \mathbf{N}_{b,j}^0$$

$$\mathbf{N}_{b,i} = \mathbf{N}_{b,i}^0 \parallel \frac{1}{2} \sum_{j \in \mathcal{N}_{a,i}} \mathbf{N}_{a,j}^0$$

where $\mathbf{N}_{a,i}$ and $\mathbf{N}_{b,i}$ indicate the i-th node embedding of atom and bond graphs, respectively; $\mathbf{N}_{a,i}^0$ and $\mathbf{N}_{b,i}^0$ their counterparts before concatenation; $\mathcal{N}_{b,i}$ the index set of bonds incident to the i-th atom; and

$\mathcal{N}_{a,i}$ the index set of the pair of atoms terminating the i-th bond. After the concatenation operation, multilayer perceptron (MLP) is applied to both atom and bond encodings for feature fusion.

Graph transformer layer. The biased multi-head attention mechanism can be formulated as:

$$a_{i,j,k} = exp\left(\frac{\mathbf{Q}_i \cdot \mathbf{K}_j}{\sqrt{d}} + e_{i,j,k}^{att}\right)$$

$$s_{i,j,k} = a_{i,j,k} \,/ \sum_j a_{i,j,k}$$

$$\mathbf{H}_i = \sum_j \mathbf{S}_{i,j} \odot (\mathbf{V}_j + \mathbf{E}_{i,j}^{val})$$

where **Q**, **K**, and **V** are respective projections of the input **X** for query, key, and value; $\mathbf{E}^{att}$ and $\mathbf{E}^{val}$ are respective projections of the node–node encoding **E** to attention bias and edge feature injection. d, which denotes dimensionality of each head, is involved for stabilising the training process. $a_{i,j,k}$ is the calculated attention of node i to node j at the hidden index k, and $s_{i,j,k}$ is the corresponding attention score. **H** is the output hidden embedding.

The node–node encoding **E** is the sum of encodings of relative position, ring structure and stereogeometric descriptors. DGT(3D) incorporates bond lengths to $\mathbf{N}_b$, atom–atom distances into $\mathbf{E}_a$, and bond–bond angles to $\mathbf{E}_b$. To inform the model with enriched local geometric representation, bond angular information is encoded with spherical harmonics functions defined on the unit sphere to ensure rotational consistency. In addition to that, chiral volume and *E/Z* isomerism information are integrated into $\mathbf{E}_a$ and $\mathbf{E}_b$, respectively, as stereogeometric descriptors to enhance DGT's performance on stereoselective tasks.

With this attention calculation hereafter referred to as $\mathrm{MHA}(\mathbf{X}, \mathbf{E}^{att}, \mathbf{E}^{val})$, the DGT layer can be given as:

$$\bar{\mathbf{H}} = \mathrm{BatchNorm}(\mathbf{X} + \mathrm{MHA}(\mathbf{X}, \mathbf{E}^{att}, \mathbf{E}^{val}))$$

$$\bar{\mathbf{X}} = \mathrm{BatchNorm}(\mathrm{FFN}(\bar{\mathbf{H}}))$$

Here, the input hidden node feature matrix $\mathbf{X}$ is added to $\mathrm{MHA}(\mathbf{X}, \mathbf{E}^{att}, \mathbf{E}^{val})$ and transformed by MLP. Feed forward network (FFN) follows to implement nonlinear transformations to each token embedding to obtain the output node features matrix $\bar{\mathbf{X}}$. The batch normalisation (BatchNorm) is applied to avoid overfitting.

For the readout part, atom and bond features are globally averaged and concatenated for predicting the molecular property as $\mathrm{MLP}(\mathrm{GAP}(\mathbf{X}_a) \parallel \mathrm{GAP}(\mathbf{X}_b))$, where GAP is the global average pooling operation. Activation function is attached after each hidden layer of the MLP.

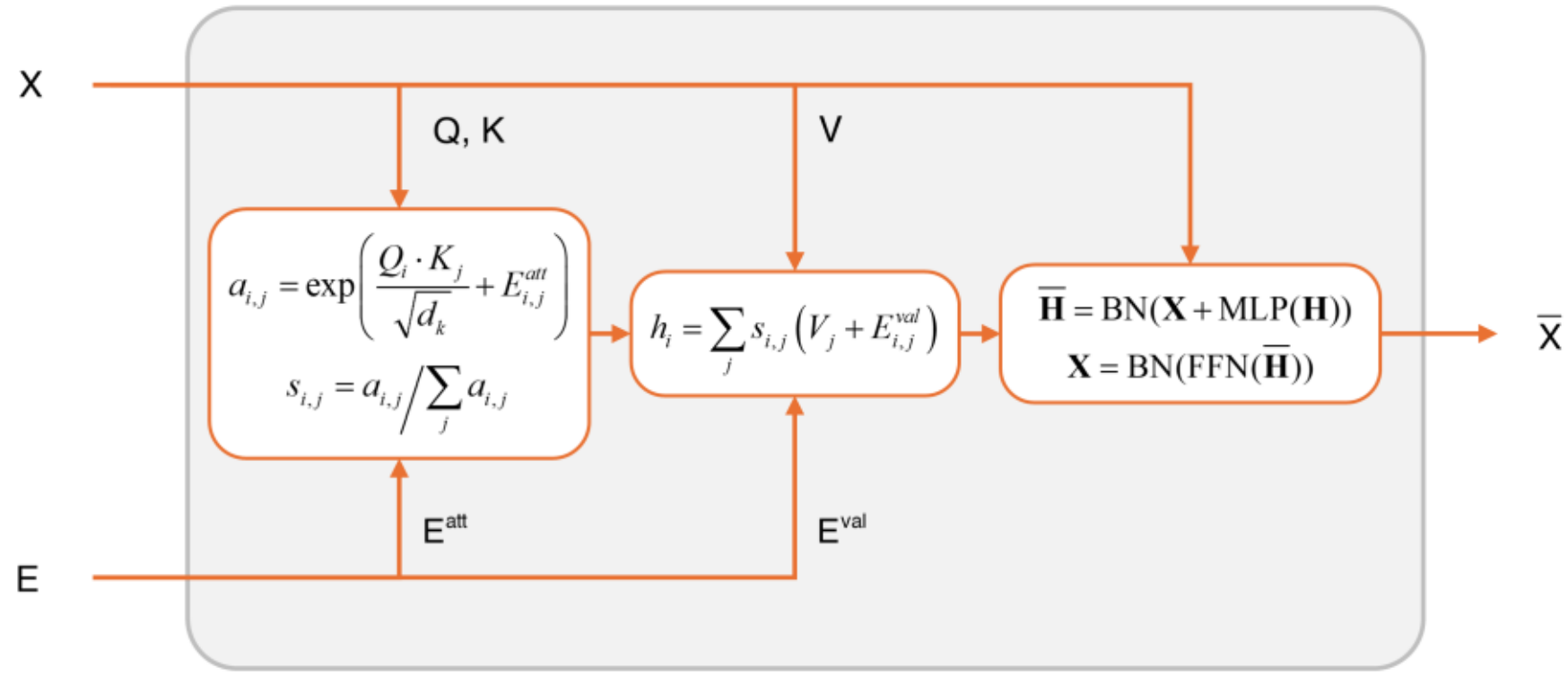

Fig. R10: Reproduced Fig. S3, detailed DGT layer architecture. This DGT layer highlights the incorporation of node–node pairwise features into both attention score calculation and passing message. Notably, only the node feature matrix is layer-wisely updated; the node–node pairwise feature matrix is the same for all DGT layers.

RC: ***3) As a new molecular representation approach, I think the authors need to evaluate the generalization of DGT to unseen and out-of-distribution molecular datasets. Especially, MoleculeNet benchmark datasets have a number of major flaws (doi: 10.1038/s43588-024-00699-0, 10.1038/s42256-024-00856-0, 10.1038/s41467-023-41948-6).***

AR: We appreciate the reviewer's insightful comment about model generalisation. We did notice this issue during the benchmarking phase and adopted the Murcko scaffold splitting strategy (as per ref. 11, doi: 10.1038/s42256-024-00856-0) to benchmark DGT's performance. Unlike a random splitting, which may lead to an overestimation of predictive accuracy due to structural redundancy between training and test sets, scaffold splitting ensures that the model is tested on chemical architectures it has not previously encountered.

However, we acknowledge that the MoleculeNet benchmark do exhibit limitations in benchmarking molecular representation approaches for real-world drug discovery applications[12,13]. The officially recommended evaluation metrics, area under the receiver operating characteristic curve (AUC-ROC) and root mean square error (RMSE), may fail to reflect real drug discovery, as the dataset is often severely imbalanced with much less actives. Furthermore, while scaffold splitting offers a more rational test than random splitting, it is often insufficient for evaluating the model's generalisation ability in terms of intra-scaffold activity cliffs, where a subtle structural change causes a drastic activity despite a conserved scaffold. In addition, the off-target effect is also ignored in the MoleculeNet benchmark.

We have expanded the applied datasets for evaluating DGT's performance in molecular learning. Following the introduction of stereogeometric descriptors to DGT, a PubChem3D subset was employed to assess the DGT's ability in *R/S* isomer recognition. The dataset consists of 2.98M conformers of 598K stereoisomers of 257K 2D graphs[3]. DGT(R/S), the specific DGT model with chiral volume incorporated, delivered a high *R/S* classification accuracy of 99.99%, outperforming other reported models under the same data splitting practice (Fig. R11). Using this as a pretraining task, DGT's performance was enhanced on downstream stereoselective Tox21 Aromatase and Estrogen Receptor (ER) classification tasks, as shown in Fig. R12 (included in the revised manuscript as Fig. 3e). Meanwhile, we are currently exploring

the utilisation of this model in reaction prediction when combined with self-driving laboratories. Please stay tuned for our continued reports of the practical application of this model.

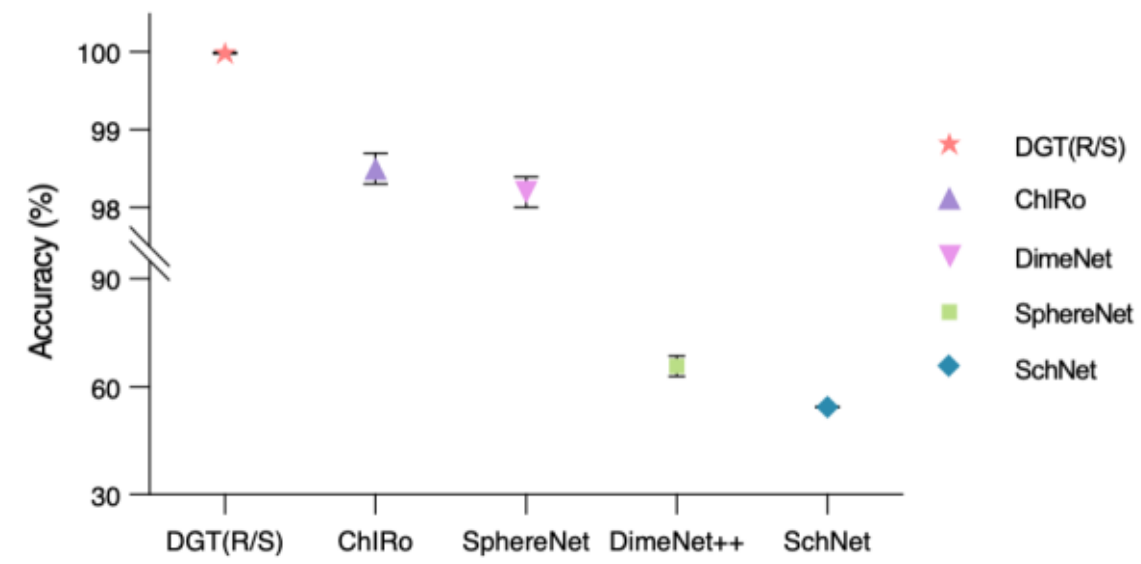

Fig. R11: Performance benchmark of DGT(R/S) for chiral classification on the PubChem3D subset against ChIRo, DimeNet, SphereNet and SchNet. Mean DGT(R/S) performance is derived from four independent experimental runs. Performance metrics of other models are adapted from ref. 3.

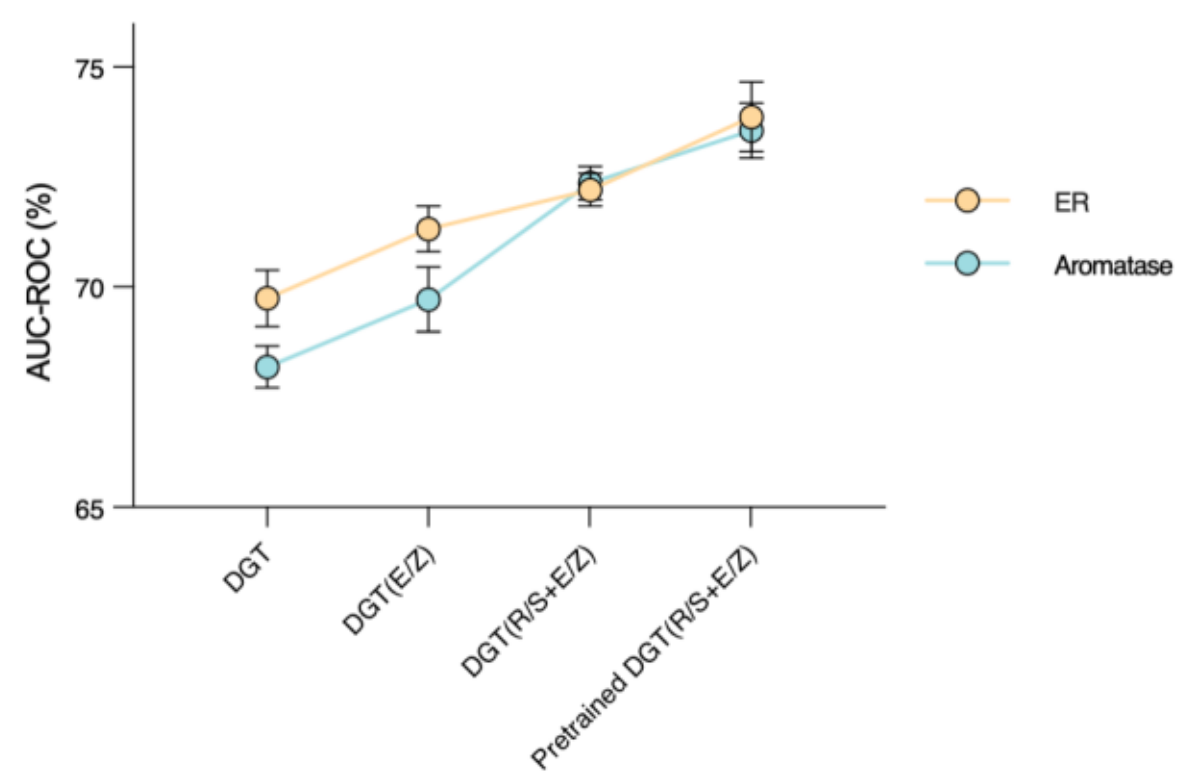

Fig. R12: Ablation analysis of DGT incorporating *R/S* and *E/Z* isomerisms on the Tox21 Aromatase and Estrogen Receptor (ER) classification tasks. Pretraining is conducted on the PubChem3D subset for enantiomer recognition. All performance results are averaged over four independent runs.

As a response, we cautiously position our work as an advancement in molecular representation for applying the powerful self-attention mechanism within the graph learning paradigm. We have revised the Discussion Section accordingly to emphasise the limitations of the MoleculeNet benchmark as discussed above.

DGT also holds the potential to promote more advanced domains such as conformation generation, molecular generative modelling, and chemical reaction prediction. It is also notable that the integration of atom–atom and bond–bond encodings introduces a computational complexity that scales quadratically with the molecular size. Model parallelism and the adoption of hierarchical or sparse attention mechanisms can be considered to extend the DGT's utility on large molecules. Furthermore, despite the performance on MoleculeNet, we recognise that a gap remains between benchmark performance and real-world molecular development. Bridging this divide necessitates metrics tailored

for extreme class imbalance, rational dataset splitting strategies that evaluate both inter- and intra-scaffold generalisation, and the accounting of side effects, such as off-target binding liabilities in drug discovery.

RC: ***4) Since the authors have run the analyses 4 times, the result plots should add the error bars.***

AR: Thanks for attention to this detail. We have added error bars to all result plots in our revised manuscript.

RC: ***5). For the ablation analysis and the 3D information incorporation in Figure 3, since the scale is too small, it is very hard to see whether there are any difference or significantly different, I would suggest to repeat the analyses for classification data sets.***

AR: Following the integration of the feature fusion module, we re-evaluated DGT's performance with weights pretrained on subsets randomly sampled from the PCQM4Mv2 dataset[14]. While pretraining remains beneficial, increasing the pretraining dataset size did not yield marginal returns in DGT's performance. We hypothesise that the previously observed gains associated with pretraining scale stem from the feature fusion learned in the last readout phase during pretraining. We have added this observation to our manuscript and Fig. R13 has been included in the Supplementary Information as Fig. S9.

DGT(3D) outperformed all other evaluated models, firmly establishing it as a highly effective 3D machine learning architecture for molecular property prediction (detailed results in Fig. S8). When pretrained on 10K random molecules sampled from the PCQM4Mv2 dataset, DGT(3D) attained prediction MAEs of 0.0306eV for $\varepsilon_{\mathrm{HOMO}}$ and 0.0240 eV for $\varepsilon_{\mathrm{LUMO}}$, though no significant correlation was observed between pretraining dataset size and downstream prediction accuracy (Fig. S9).

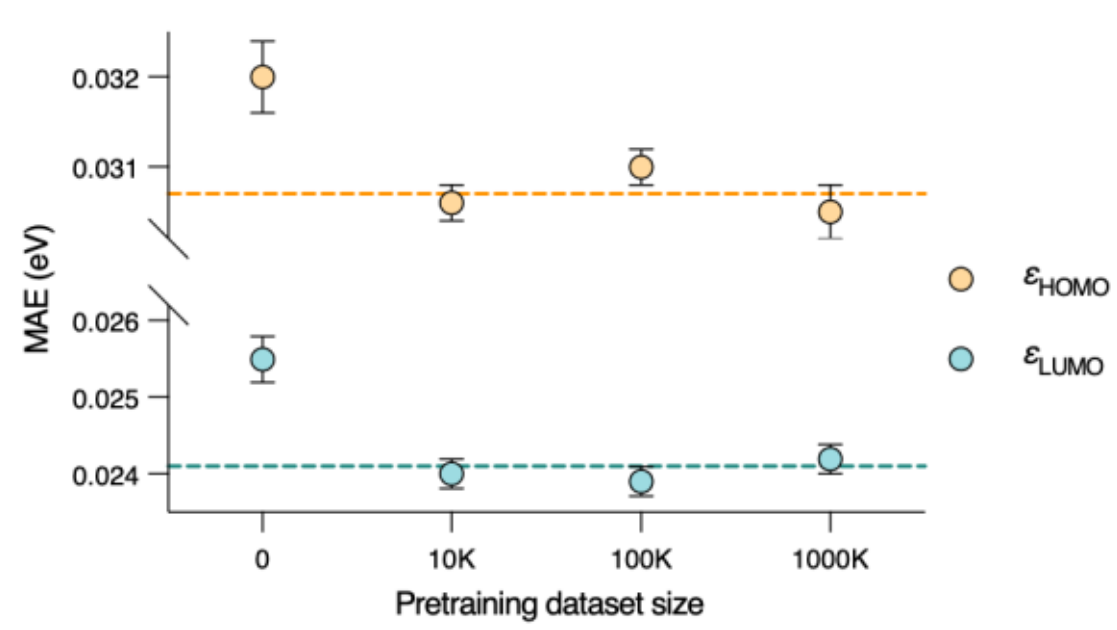

Fig. R13: Impact of pretraining data scale on DGT(3D) performance. Performance trajectory of DGT(3D) is evaluated when pretrained on subsets of the PCQM4Mv2 dataset of increasing size (0, 10K, 100K, and 1M molecules) for predicting $\varepsilon_{\mathrm{HOMO}}$ and $\varepsilon_{\mathrm{LUMO}}$.

Reviewer 3

RC: ***The manuscript is well-structured and clearly presented. The authors propose the dual graph transformer (DGT) model, which effectively integrates atom and bond representations to enhance molecular property predictions. They provide a thorough benchmarking of DGT across multiple datasets, demonstrating its superior performance compared to existing state-of-the-art models. However, there are several critical weaknesses that need to be addressed.***

AR: Thanks for your appreciation of our work. We hope our responses outlined below can address your major concern about the model structure design, as well as some others.

RC: ***1. Limited Interaction Representation: The manuscript concludes with a simple concatenation of atom representation and bond representation. As shown in Figure 1, this approach fails to effectively capture the intricate interactions between atoms and bonds within the molecular structure. Recent studies, such as those utilizing attention-based mechanisms or message-passing frameworks, have demonstrated that explicitly modeling the interactions between different types of nodes can significantly enhance the predictive power of graph neural networks (GNNs). The lack of a mechanism to model these interactions limits the model's ability to fully leverage the complementary information provided by both representations.***

AR: We thank the reviewer for this insightful comment on the model structure design regarding feature fusion between atoms and bonds. A feature fusion module has been positioned upstream the stacked graph transformer layers in DGT. The renewed DGT structure is shown in Fig. R14, where atom and bond encodings are fused in the feature fusion module. Chiral volume and *E/Z* isomerism information are also added as optional molecule encodings for stereoselective tasks.

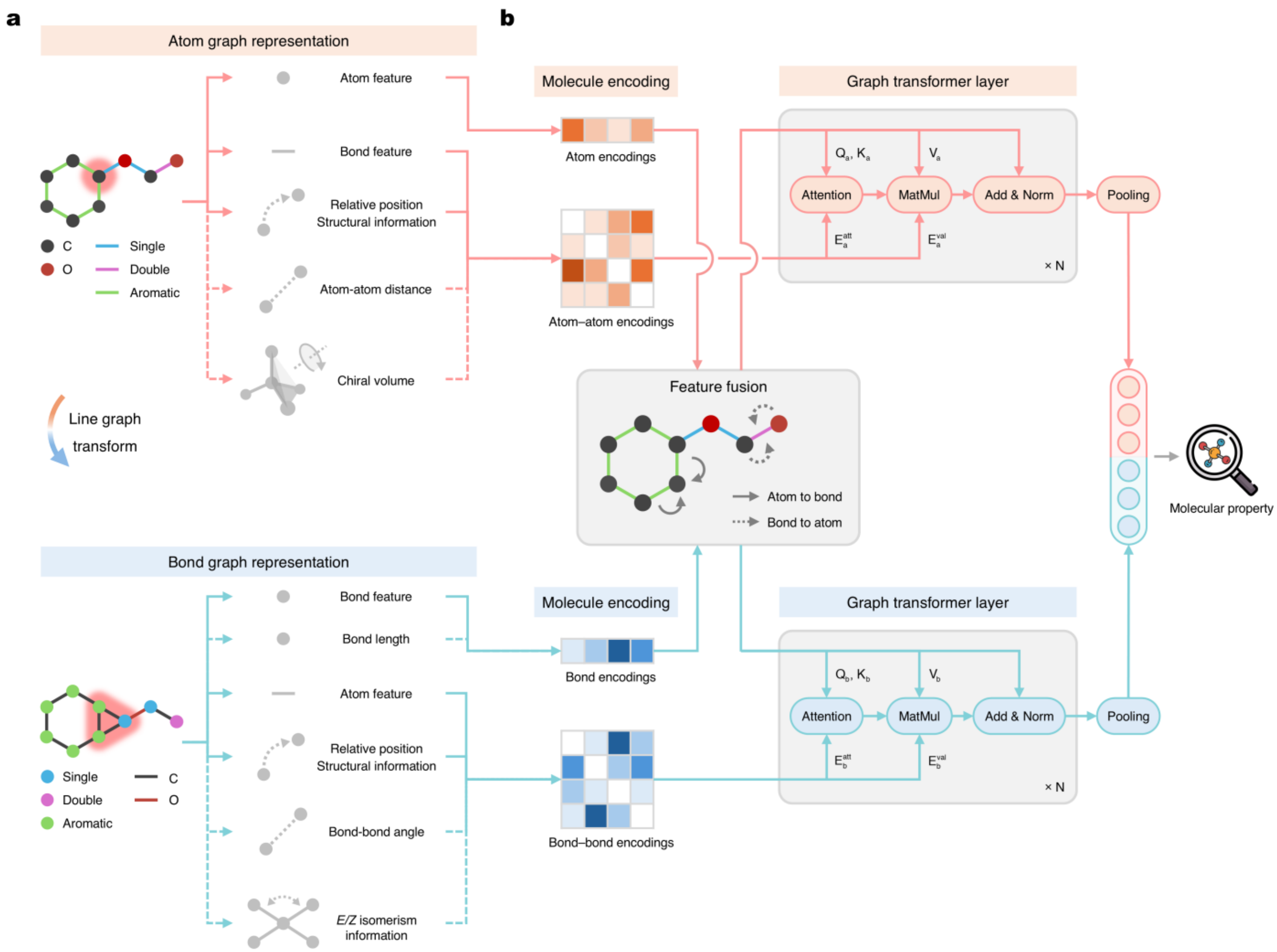

Fig. R14: Reproduced Fig. 1, an illustration of the Dual Graph Transformer (DGT). **a**, Atom and bond graph representations of molecules. Positions of atoms and bonds are exchanged in the dual graph representation, accommodating atom and bond features, relative position, and structural information. For instance, the highlighted *ipso* carbon in the atom graph corresponds to the three highlighted edges in the corresponding bond graph. Bond length, atom–atom distance, and bond–bond angle can also be integrated into DGT as geometric information when available. DGT is able to incorporate chiral volume and *E/Z* isomerism information to enhance performances on stereoselective tasks. **b**, Molecule encodings, feature fusion, and graph transformer structure. DGT encodes molecules into atom and bond vectors, atom–atom matrices, and bond–bond matrices. Atom and bond encodings are fused before the query-key-value operation by stacked graph transformer layers, followed by the pooling layer for predicting molecular properties.

Following the integration of the feature fusion module, we re-evaluated the DGT model (Fig. R15) on the MoleculeNet dataset. The core conclusions of our study remain unchanged, with a slight performance gain observed on the QM9 dataset, as shown in Fig. R16. The atom–bond feature fusion led to a decrease in MAE across 10 of the 12 QM9 subtasks, resulting in an average reduction of approximately 10%.

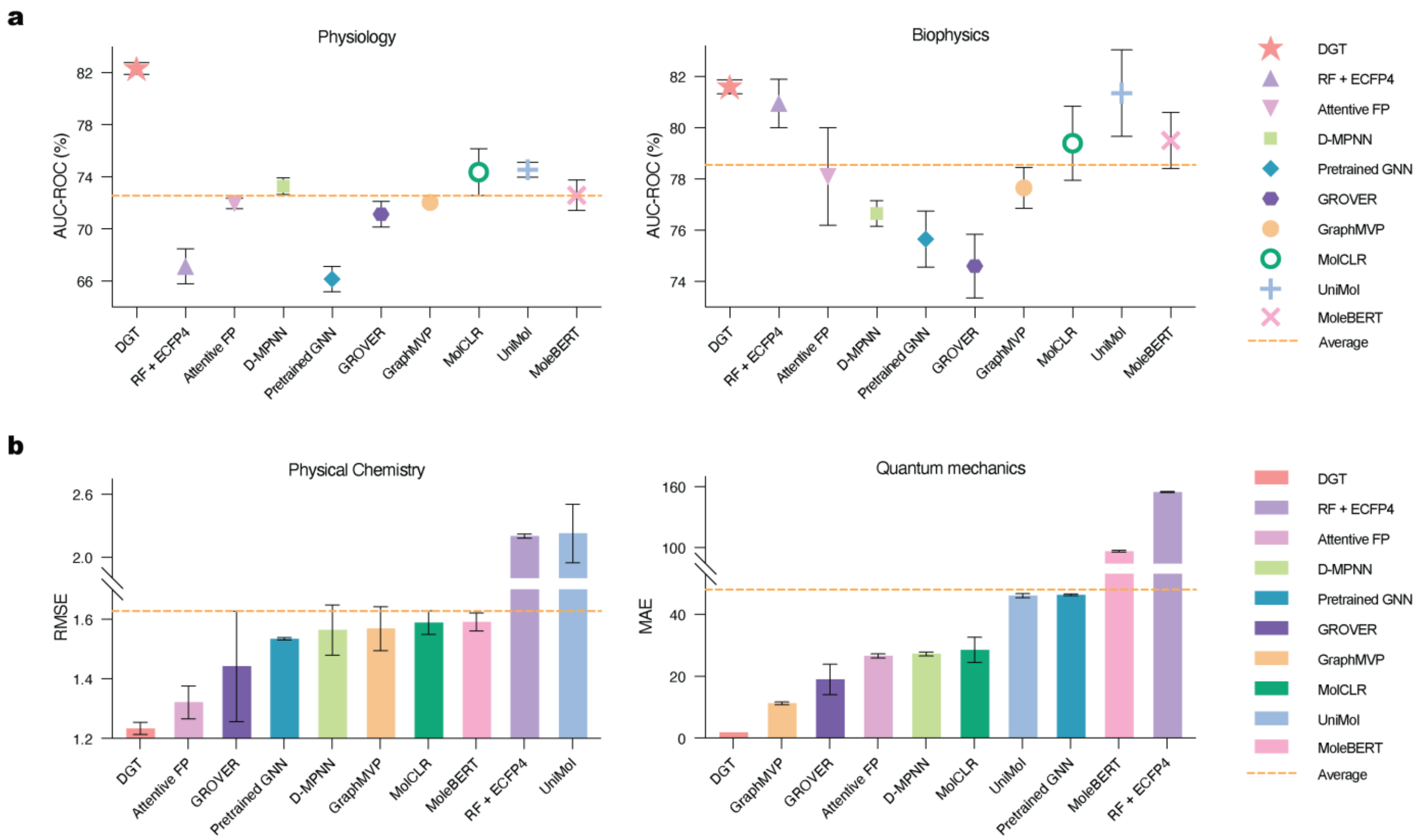

Fig. R15: Reproduced Fig. 2, benchmarking DGT against baselines across four domains. a, Performance comparison of DGT against baselines for predicting categorical molecular properties in physiology and biophysics domains. b, Performance comparison of DGT against baselines for predicting numerical molecular properties in physical chemistry and quantum mechanics domains.

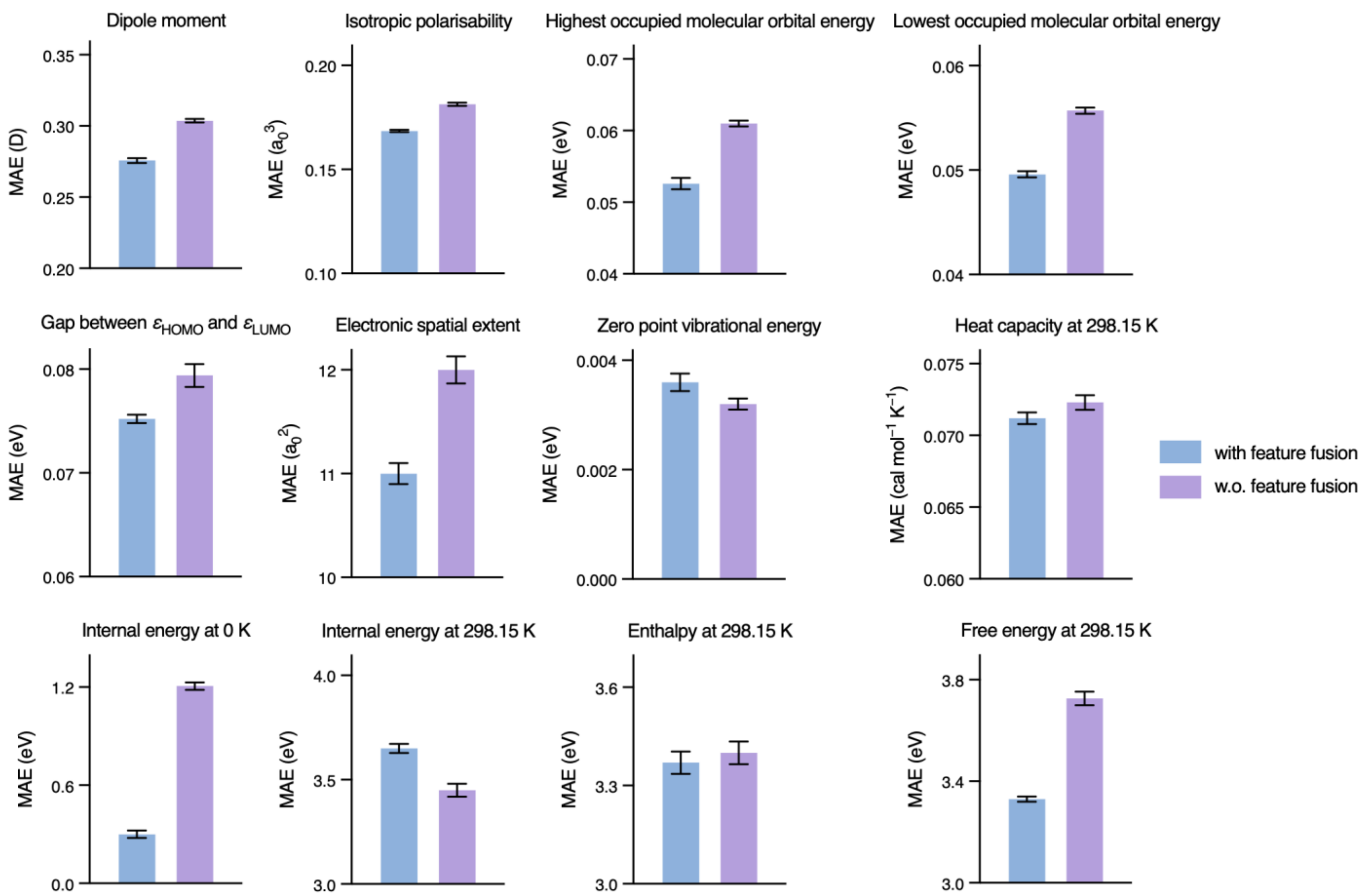

Fig. R16: Performance improvement on the QM9 dataset after introducing the feature fusion module to DGT.

We have revised the DGT overview Section accordingly and updated the results of DGT on the MoleculeNet benchmark in the main text.

> The feature fusion module positioned upstream stacked graph transformer layers employs a bidirectional aggregation strategy to synthesise an integrated representation: bond embeddings are augmented by concatenating their feature vectors with the mean representation of their incident atoms. Conversely, atomic embeddings are enriched by incorporating the average of their adjacent edge representations (Fig. 1b). The graph transformer layer extends the conventional query-key-value operation by merging the pairwise feature matrices from atom and bond graphs into their respective attention score matrices and passing messages, allowing DGT to learn both local and global dependencies for atoms and bonds.

RC: ***2. Insufficient Utilization of Structural and Spatial Information: The incorporation of structural and spatial information in the model appears to be minimal, as it is treated merely as input features with relatively low significance in the overall architecture. To enhance the predictive capability of the DGT model, I suggest that the authors consider employing advanced techniques, such as 3D equivariant***

graph neural networks (e.g., SchNet and DimeNet), which can better capture the geometric and spatial relationships inherent in molecular structures. These methods have shown promise in leveraging 3D spatial information to improve predictions of molecular properties, thus providing a more robust representation of molecular behavior.

AR: We appreciate the reviewer's constructive suggestion. We did reference to 3D equivariant graph neural networks, such as SchNet and DimeNet, when designing DGT. Bond length, atom–atom distance and bond–bond angle are captured as 3D information. Notably, these encoded features are also equivariant to rotation and translation of the molecular coordinate system. Besides, drawing inspiration from SchNet and DimeNet, the normalised spherical harmonics are employed to provide an orthonormal representation of angular dependencies (Fig. S2). This approach ensures a smooth, symmetry-aware encoding of the bond–bond angular space.

In addition to that, to specifically capture stereochemical information for molecular machine learning, we have adopted chiral volume and *E/Z* isomerism information for molecule encoding in DGT, as shown in Fig. R14. For a chiral atom, the chiral volume of a directed bond is calculated as the signed volume of the other three directed bonds arranged using the Cahn-Ingold-Prelog priority rules[1]. Given vectors of those three ordered directed bonds as $\boldsymbol{v}_1$, $\boldsymbol{v}_2$ and $\boldsymbol{v}_3$, the signed chiral volume is calculated as $(\boldsymbol{v}_1 \times \boldsymbol{v}_2) \cdot \boldsymbol{v}_3$, which is dependent on chirality and invariant to bond rotations[2]. We benchmarked the DGT model incorporating chiral volume, hereafter denoted as DGT(R/S), on a published subset of PubChem3D for *R/S* classification (Fig. R17, now included in Supplementary Information as Fig. S9). The dataset consists of 2.98M conformers of 598K stereoisomers of 257K 2D graphs[3]. DGT(R/S) delivers a high *R/S* classification accuracy of 99.99%, outperforming other reported models under the same data splitting practice.

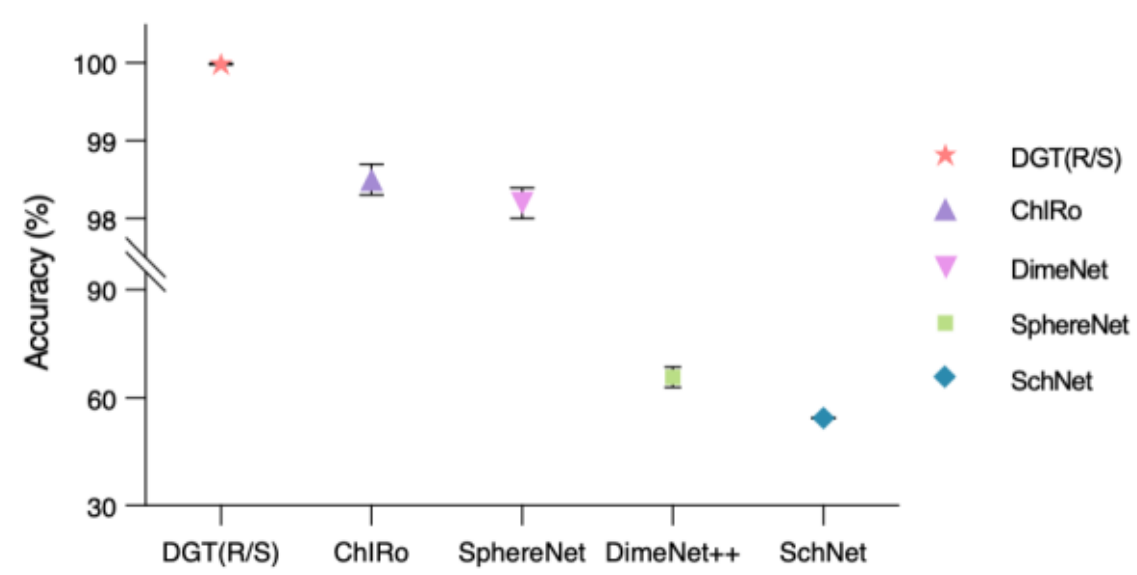

Fig. R17: Performance benchmark of DGT(R/S) for chiral classification on the PubChem3D subset against ChIRo, DimeNet, SphereNet and SchNet. Mean DGT(R/S) performance is derived from four independent experimental runs. Performance metrics of other models are adapted from ref. 3.

To evaluate the specific contributions of stereochemical encodings, we then conducted ablation analysis on the Tox21 Aromatase and Estrogen Receptor (ER) classification tasks. These targets are selected because their biological activity is highly sensitive to the steroidal scaffold, where both *R/S* chirality and *E/Z* isomerism have a prominent impact[4–6]. As hypothesised, adding stereochemical information to DGT improved its classification performance (Fig. R18). AUC-ROC was increased by about 1.5% for both tasks after incorporating the *E/Z* isomerism information into the bond–bond encodings (DGT(E/Z)). This performance was enhanced in DGT(R/S+E/Z), which, compared to DGT(E/Z), further incorporates chiral volume into the atom–atom encodings to resolve enantiomeric ambiguity. Further leveraging a pretraining

strategy on the PubChem3D subset for enantiomer recognition resulted in an approximate 1.4% gain for both tasks.

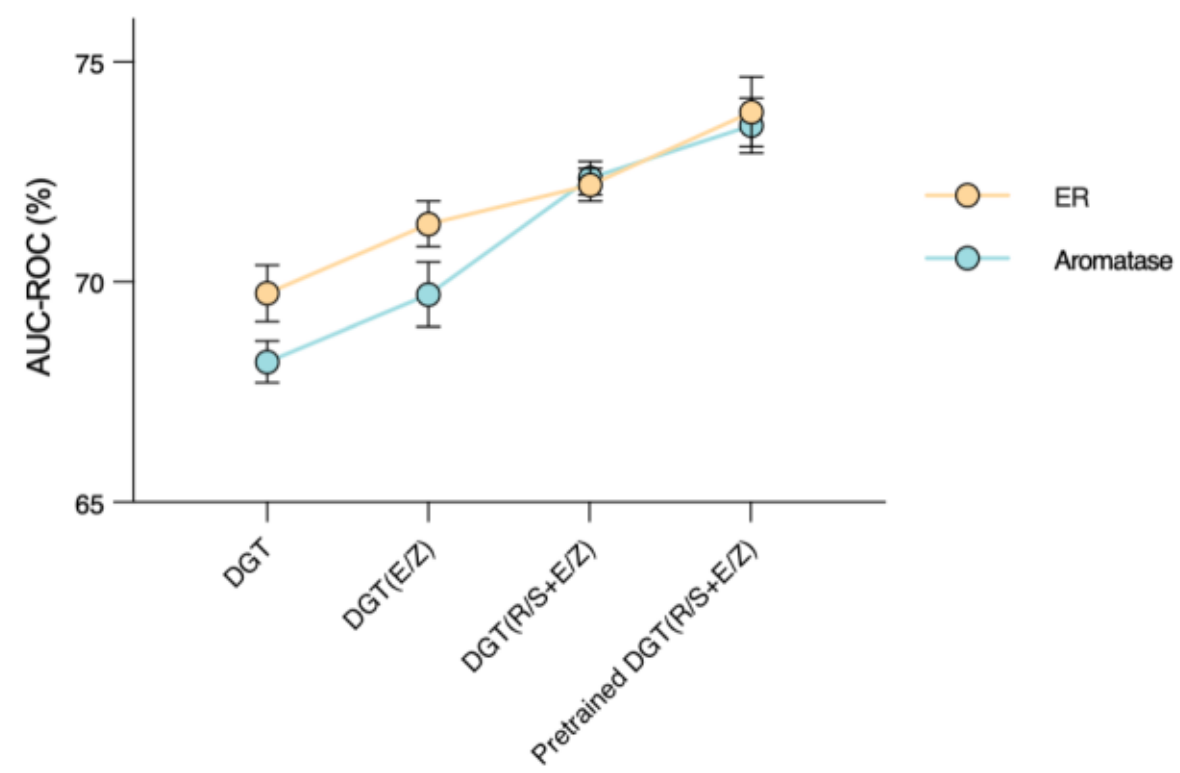

Fig. R18: Ablation analysis of DGT incorporating *R/S* and *E/Z* isomerisms on the Tox21 Aromatase and Estrogen Receptor (ER) classification tasks. Pretraining is conducted on the PubChem3D subset for enantiomer recognition. All performance results are averaged over four independent runs.

In response, we have revised our manuscript accordingly to reflect the modified model structure (Fig. 1). Since chirality and *E/Z* isomerism are features primarily relevant to specific stereochemical prediction tasks (such as those involving protein-ligand interactions), these two encodings are evaluated in Fig. 3e for the Tox21 Aromatase and ER classification tasks but not included in the DGT model for the general benchmark in Fig 2. Other subfigures in Fig. 3 have been also updated to align with the model structure modification.

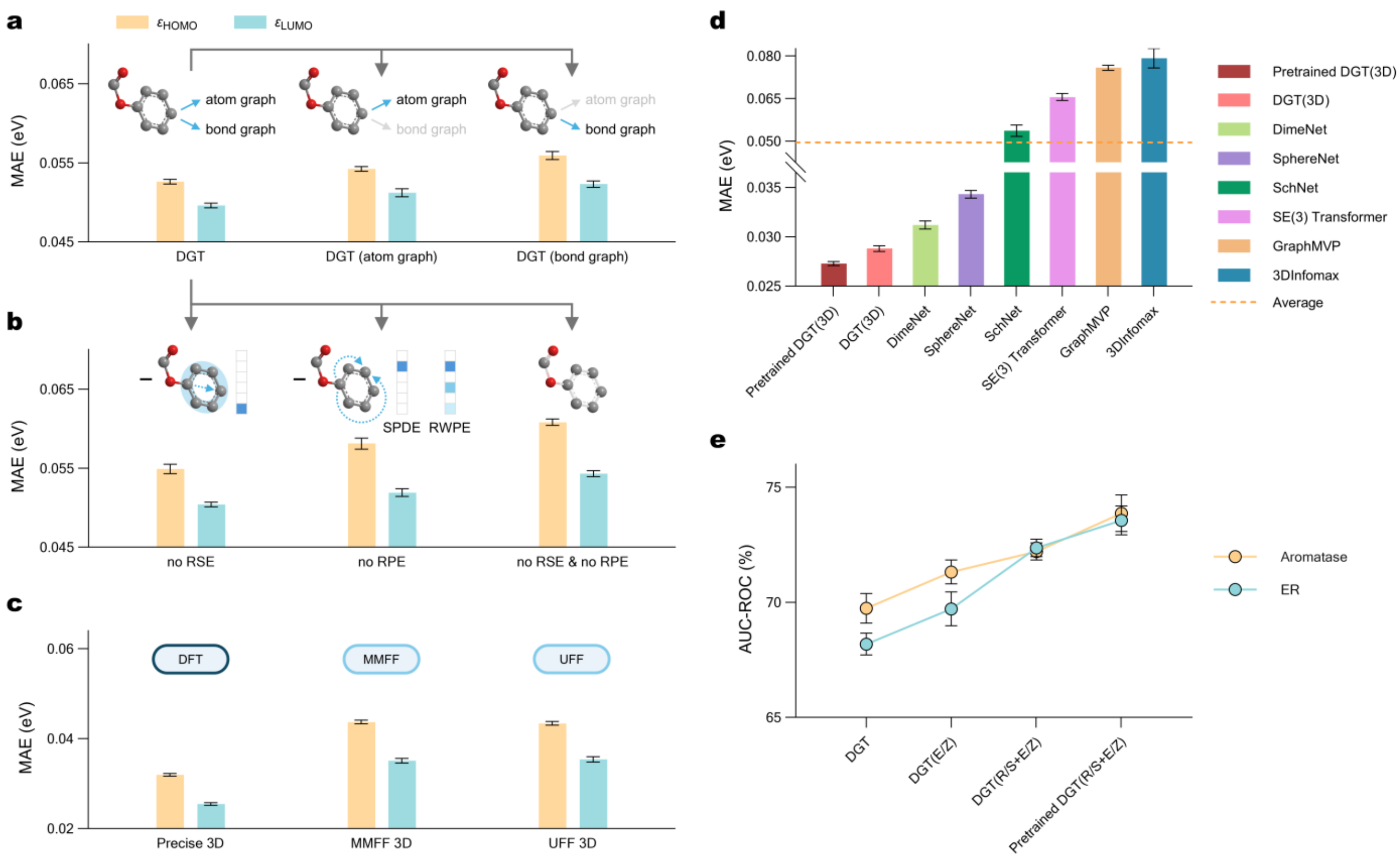

Fig. R19: Reproduced Fig. 3, performance investigation of DGT and stereogeometric information incorporation. The efficacy of incorporating 3D information was evaluated through HOMO and LUMO energy predictions on the QM9 dataset, while the impact of R/S chirality and E/Z isomerism was validated using the Tox21 Aromatase and Estrogen Receptor (ER) classification tasks. **a**, Ablation study evaluating the dual graph representation. **b**, Ablation study of graph-specific encodings. **c**, Effect of incorporating 3D information at different fidelity levels on DGT's predictive performance. **d**, Comparative assessment of the 3D information-augmented DGT, denoted as DGT(3D), against state-of-the-art models for 3D molecular learning using the average prediction MAE of HOMO and LUMO energies. The pretraining of DGT(3D) is conducted on molecules from the PCQM4Mv2 dataset targeting the HOMO–LUMO energy gap. **e**, Ablation study evaluating the contribution of *R/S* chirality and *E/Z* isomerism to DGT performance. A reported PubChem3D subset for enantiomer recognition[3] is adopted for pretraining.

We have updated the overview Section to introduce the integration of chiral volume and *E/Z* isomerism information to DGT.

> Additionally, this dual graph representation accommodates diverse stereogeometric information: Bond lengths can be embedded within bond features; atom–atom distances and bond–bond angles should fit into the pairwise feature matrices of atom and bond graphs, respectively; chiral volume can be captured as a stereochemical descriptor in the atom–atom encodings; relationships of bonds in *E/Z* isomers align with the dimensions of bond–bond encodings. To smoothen the continuous geometric information of bond–bond angles, the normalised spherical harmonics are employed to provide an orthonormal representation of angular dependencies (Fig. S2).

The discussion pertaining to Figs. 3d and 3e has been revised as follows:

~~As depicted in Fig. 3d, we meticulously assessed the performance trajectory of DGT(3D) when pretrained on subsets of the PCQM4Mv2 dataset of increasing size (0, 10K, 100K, and 1M molecules) for predicting ε_{HOMO} and ε_{LUMO}. Enlarging the pretraining dataset delivered a monotonic reduction in MAE, underscoring the profound benefit of large-scale pretraining in facilitating DGT(3D) to learn a more generalised molecular representation for relevant downstream tasks. Quantitatively, the ultimate prediction MAEs for ε_{HOMO} and ε_{LUMO} were as low as 0.0302 eV and 0.0231 eV, respectively, corresponding to considerate improvements of 9.3% and 7.2% attributable to pretraining.~~

Fig. 3ed compares DGT(3D) against some established state-of-the-art models designed for 3D molecular learning. The benchmark models included SchNet, DimeNet, SphereNet, SE(3) Transformer, GraphMVP, and 3DInfomax. SchNet, DimeNet, and SphereNet represented 3D GNNs that use rotationally invariant features to predict scalar molecular properties. The SE(3) Transformer was included as a typical equivariant GNN that is robust to 3D roto-translations of molecules. GraphMVP and 3DInfomax were models adopting contrastive learning to enrich 2D graph structure embeddings with 3D conformational information. DGT(3D), whether pretrained or not, outperformed all other evaluated models, firmly establishing it as a highly effective 3D machine learning architecture for molecular property prediction. When pretrained on 10K random molecules sampled from the PCQM4Mv2 dataset, DGT(3D) attained prediction MAEs of 0.0306 eV for ε_{HOMO} and and 0.0240 eV for ε_{LUMO}, though no significant correlation was observed between pretraining dataset size and downstream prediction accuracy (Fig. S9).

The efficacy of integrating chiral volume and *E/Z* isomerism with DGT was validated on the Tox21 Aromatase and ER tasks, both of which exhibit high stereoselectivity due to their steroidal-binding motifs. As hypothesised, the inclusion of stereochemical descriptors consistently improved the classification performance (Fig. 3e). Incorporating *E/Z* isomerism information into the bond–bond encodings (DGT(E/Z)) yielded an approximate 1.5% increase in AUC-ROC across both tasks. This performance was further enhanced in DGT(R/S+E/Z), which incorporates chiral volume into the atom–atom encodings to resolve enantiomeric ambiguity. Based on this architecture, a pretraining strategy was leveraged using a PubChem3D subset, where DGT outperformed other stereochemical models in enantiomer recognition (Fig. S10). This specific pretraining stage facilitated an additional gain of about 1.4% in AUC-ROC for both Tox21 tasks.

RC: ***3. Inadequate Discussion of Encoding Methods: Although the manuscript briefly introduces various encoding methods, such as relative positional encoding (RPE) and ring structural encoding (RSE), it lacks a comprehensive discussion on the limitations of existing methods for atom and edge representations. For instance, while RPE and RSE are valuable, they may not fully capture the complex dependencies that exist within molecular graphs. Additionally, the manuscript does not adequately address how edge features are utilized in current implementations, particularly regarding their aggregation and contribution to updating node features prior to attention calculations. A more thorough examination of these aspects would provide clearer insights into the effectiveness of the proposed dual graph representation.***

AR: Thanks for your valuable comment, which has allowed us to broaden our discussion on graph encoding methodologies. While the current DGT framework adopts RPE and RSE for their intuitive representation

of node–node interactions, the architecture is flexible to incorporate other topological or structural priors. These include descriptors derived from the Graph Laplacian[15], spectral kernels[16], or Green's functions[17], as well as hierarchical features from specific molecular motifs[18]—such as the chiral volume and *E/Z* isomerism information introduced in this work. We have updated the Discussion Section to emphasise that DGT can be further extended to these representation methods to refine molecular encoding.

> Such structure generation model can be added to the current workflow of DGT as an end-to-end approach. The node–node embeddings within DGT can be further extended to incorporate structural information derived from specific molecular motifs and topological descriptors derived from the Graph Laplacian, spectral kernels, or Green's functions.

Regarding the role of edge features in DGT, we have added explanations to the Methods Section. In detail, the edge features are merged into the node–node encoding $\mathbf{E}$ and projected into $\mathbf{E}^{att}$ and $\mathbf{E}^{val}$, which are respectively appended to the attention matrix and the value projection of the input $\mathbf{X}$.

> The biased multi-head attention mechanism can be formulated as:
>
> $$a_{i,j,k} = exp\left(\frac{\mathbf{Q}_i \cdot \mathbf{K}_j}{\sqrt{d}} + e^{att}_{i,j,k}\right)$$
>
> $$s_{i,j,k} = a_{i,j,k} \,/ \sum_j a_{i,j,k}$$
>
> $$\mathbf{H}_i = \sum_j \mathbf{S}_{i,j} \odot (\mathbf{V}_j + \mathbf{E}^{val}_{i,j})$$
>
> where $\mathbf{Q}$, $\mathbf{K}$, and $\mathbf{V}$ are respective projections of the input $\mathbf{X}$ for query, key, and value; $\mathbf{E}^{att}$ and $\mathbf{E}^{val}$ are respective projections of the node–node encoding $\mathbf{E}$ to attention bias and edge feature injection. d, which denotes dimensionality of each head, is involved for stabilising the training process. $a_{i,j,k}$ is the calculated attention of node i to node j at the hidden index k, and $s_{i,j,k}$ is the corresponding attention score. $\mathbf{H}$ is the output hidden embedding.

References

1. Cahn, R. S., Ingold, C. & Prelog, V. Specification of Molecular Chirality. *Angew. Chem. Int. Ed.* https://doi.org/10.1002/anie.196603851 (1966) doi:10.1002/anie.196603851.
2. Evans, P. R. An introduction to stereochemical restraints. *Acta Crystallogr. D Biol. Crystallogr.* **63**, 58–61 (2007).
3. Adams, K., Pattanaik, L. & Coley, C. W. Learning 3D Representations of Molecular Chirality with Invariance to Bond Rotations. Preprint at https://doi.org/10.48550/arXiv.2110.04383 (2021).
4. Ford, B. M., Franks, L. N., Radominska-Pandya, A. & Prather, P. L. Tamoxifen Isomers and Metabolites Exhibit Distinct Affinity and Activity at Cannabinoid Receptors: Potential Scaffold for Drug Development. *PLoS ONE* **11**, e0167240 (2016).

5. Hou, Y. *et al.* Benzestrol Isomer Stereochemistry Determines the Distinct Estrogenic Activities and Conformations of the Eight Isomers When Bound to Estrogen Receptor α. *ACS Pharmacol. Transl. Sci.* **8**, 2526–2535 (2025).
6. Chayawan, C., Toma, C., Benfenati, E. & Caballero Alfonso, A. Y. Towards an Understanding of the Mode of Action of Human Aromatase Activity for Azoles through Quantum Chemical Descriptors-Based Regression and Structure Activity Relationship Modeling Analysis. *Molecules* **25**, 739 (2020).
7. Morgen, M. *et al.* Spiroepoxytriazoles Are Fumagillin-like Irreversible Inhibitors of MetAP2 with Potent Cellular Activity. *ACS Chem. Biol.* **11**, 1001–1011 (2016).
8. Shiau, A. K. *et al.* The Structural Basis of Estrogen Receptor/Coactivator Recognition and the Antagonism of This Interaction by Tamoxifen. *Cell* **95**, 927–937 (1998).
9. Southey, M. W. Y. & Brunavs, M. Introduction to small molecule drug discovery and preclinical development. *Front. Drug Discov.* **3**, (2023).
10. Hong, H. *et al.* A hierarchical interaction message net for accurate molecular property prediction. *Commun. Chem.* **9**, 150 (2026).
11. Boulougouri, M., Vandergheynst, P. & Probst, D. Molecular set representation learning. *Nat. Mach. Intell.* **6**, 754–763 (2024).
12. Deng, J. *et al.* A systematic study of key elements underlying molecular property prediction. *Nat. Commun.* **14**, 6395 (2023).
13. Durant, G., Boyles, F., Birchall, K. & Deane, C. M. The future of machine learning for small-molecule drug discovery will be driven by data. *Nat. Comput. Sci.* **4**, 735–743 (2024).
14. Hu, W. *et al.* OGB-LSC: A Large-Scale Challenge for Machine Learning on Graphs. Preprint at https://arxiv.org/abs/2103.09430 (2021).
15. Mialon, G., Chen, D., Selosse, M. & Mairal, J. GraphiT: Encoding Graph Structure in Transformers. Preprint at https://doi.org/10.48550/arXiv.2106.05667 (2021).
16. Kreuzer, D., Beaini, D., Hamilton, W. L., Létourneau, V. & Tossou, P. Rethinking Graph Transformers with Spectral Attention. Preprint at https://doi.org/10.48550/arXiv.2106.03893 (2021).
17. Venturella, C., Hillenbrand, C., Li, J. & Zhu, T. Machine Learning Many-Body Green's Functions for Molecular Excitation Spectra. *J. Chem. Theory Comput.* **20**, 143–154 (2024).
18. Chen, D., O'Bray, L. & Borgwardt, K. Structure-Aware Transformer for Graph Representation Learning. in *Proceedings of the 39th International Conference on Machine Learning* 3469–3489 (2022).

Authors' Response to Reviews of

Enhancing molecular property prediction via transformer with dual graph representation

Shuyuan Zhang, Alexei A. Lapkin
*Nature Communications, *E-mail: sz469@cam.ac.uk*

RC: ***Reviewers' Comment***, AR: Authors' Response, □ Manuscript Text

We appreciate the editor and reviewers for their valuable feedback and suggestions. We believe that we have addressed all comments in this point-by-point response and made the requisite revisions to our manuscript accordingly, which have significantly strengthened this work. All relevant changes to the manuscript text are reproduced in this response, with newly added text highlighted in blue. Reviewers' comments are also reproduced in full below.

Reviewer 1

RC: ***The authors have made meaningful improvements in response to my comments. The updated model now includes stereochemical descriptors (chiral volume for R/S and an E/Z-related bond encoding), and the additional ablations on stereoselective tasks help support the claims. The added reporting of parameter counts/FLOPs is also appreciated.***

AR: We thank the reviewer for the appreciation of our revisions regarding the model structure, parameter amount, FLOPs, and ablation studies on stereoselective tasks. In response to your concern regarding the credibility of the newly added *R/S* chirality benchmark results, we adopted the same data-splitting protocol used in the original publication and further confirmed that it does follow a strict 2D graph-level split. We hope that the detailed explanation provided below fully clarifies this issue.

RC: ***I recommend minor revision, mainly to strengthen the credibility and reproducibility of the new R/S chirality benchmark result (reported extremely high accuracy). Please clarify the splitting protocol (2D-graph vs stereoisomer vs conformer level), whether any conformers/stereoisomers from the same 2D graph can appear in different splits, and any de-duplication performed. Ideally, please also provide either (i) results under a strict 2D-graph-level split, or (ii) quantitative overlap statistics demonstrating no cross-split duplicates, and report an additional metric such as balanced accuracy/AUROC (mean±std over runs). With these clarifications, the revision should be suitable for acceptance.***

AR: We appreciate the reviewer's attention to this important detail. In our study, we adopted the open-source pickle files released by Adams et al. (https://figshare.com/s/e23be65a884ce7fc8543) as the raw dataset[1]. The original authors partitioned the conformers according to their SMILES strings to ensure that conformers derived from the same 2D molecular graph do not appear across different training, validation, and test sets. Specifically, as stated in their publication: "We use a subset of PubChem3D containing 466K conformers of 78K enantiomers with one tetrahedral chiral center. We create 70/15/15 train/validation/test splits, keeping conformers corresponding to the same 2D graphs in the same partition." Therefore, the adopted data split follows a strict 2D-graph-level separation protocol, preventing conformer-level data leakage between different dataset partitions.

Actually, the performance advantage of DGT(R/S) over the second-best ChIRo primarily arises from the explicit encoding of chiral volume through the Cahn-Ingold-Prelog (CIP) canonical ordering. Although ChIRo is also designed to be SE(3)-invariant, it relies mainly on torsional information to characterise relative geometric relationships along molecular bonds. However, R/S stereochemical assignment requires a canonical ordering of substituents around a stereocentre, whereas such ordering is explicitly defined in DGT(R/S) through the CIP rules. This explicit chirality-sensitive representation provides a deterministic local stereochemical frame for distinguishing enantiomeric configurations, thereby leading to improved R/S recognition performance.

We have revised the Methods Section and Supplementary Information accordingly to further emphasise both the data-splitting strategy and the underlying reason for the improved performance of DGT(R/S) in *R/S* enantiomer recognition.

> Randomly sampled subsets of the PCQM4Mv2 dataset were utilised as the training set for investigating the pretraining of DGT(3D). The chiral classification ability of DGT(R/S) was validated on a subset of

PubChem3D released by Adams et al., which is available at https://figshare.com/s/e23be65a884ce7fc8543.

***R/S* enantiomer recognition.** A subset of PubChem3D was adopted to validate the introduced atom–atom chiral-volume encoding for chiral centre classification. The dataset comprises multiple OMEGA-generated conformers of organic molecules containing up to 50 heavy atoms and 15 rotatable bonds. The subset consists of 466K conformers of 78K enantiomers with one tetrahedral chiral centre. Each stereoisomer has at least two conformers, and each graph has at least two stereoisomers. The dataset was partitioned into training, validation, and testing sets using a 70/15/15 split while ensuring that all conformers associated with the same 2D graphs were assigned to the same data partition.

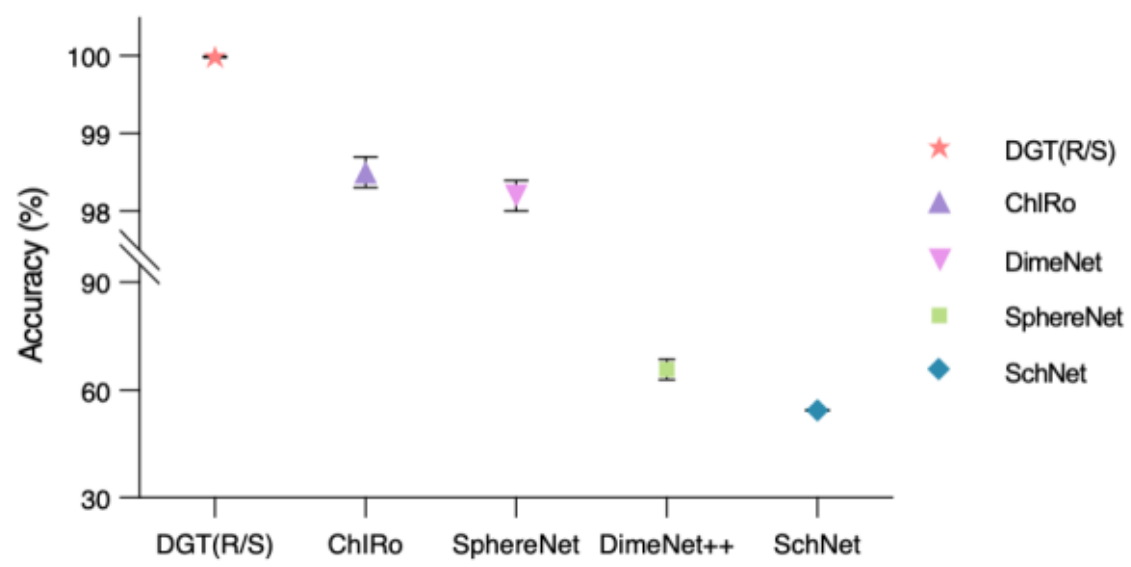

Fig. R1: Performance benchmark of DGT(R/S) for chiral classification on the PubChem3D subset against ChIRo, DimeNet, SphereNet and SchNet. Mean DGT(R/S) performance is derived from four independent experimental runs. Performance metrics of other models are adapted from ref. 1. The performance advantage of DGT(R/S) primarily arises from the explicit encoding of chiral volume based on the Cahn-Ingold-Prelog ordering rules, whereas other models must implicitly infer a canonical ordering of substituents around a stereocentre from atom identities and geometric information.

Reviewer 2

RC: ***Thanks for the authors to make the detailed revisions. Most of my comments have been addressed. I just have below two minors:***

AR: We thank the reviewer for the positive evaluation of our revised manuscript and for recognising that the majority of the previous comments have been satisfactorily addressed. We are also grateful for the additional comments provided in this round of review, which helped us identify and correct an important error in the model evaluation metric. We hope that the revisions made in response to these comments have fully addressed the remaining concerns and further strengthened the manuscript.

RC: ***1. In Fig. 2b, the physical chemistry domain reports averaged RMSE values. However, in Supplementary Fig. S6, the metric for FreeSolv appears to be MAE rather than RMSE. It may be helpful for the authors to clarify this for consistency.***

AR: We apologise for this typographical error. Upon review of the configuration files for FreeSolv, we confirm

that the metric used is RMSE rather than MAE. We have additionally checked all figure labels to ensure consistency and confirm that no further discrepancies are present.

RC: ***2. In the physiology domain, the major performance gain appears to come from the SIDER dataset (Fig. S4), where the improvement is particularly significant. It would be interesting if the authors could further discuss why the proposed method is especially effective on this dataset, and whether this may relate to the dual-graph representation or the incorporation of structural/stereochemical information.***

AR: We sincerely thank the reviewer for drawing our attention to this important detail. For multi-label classification tasks, the appropriate evaluation metric is the macro-average AUC-ROC, in which the AUC values are averaged across classes rather than across individual predictions[2]. However, due to an oversight, we inadvertently used the pre-set micro-average AUC-ROC setting during model evaluation.

After identifying and correcting this error, we re-evaluated our model on all multi-label classification datasets, including Tox21, ClinTox, and SIDER. The updated results are presented in Fig. R2. Despite the correction, DGT continues to demonstrate competitive performance, achieving the best results on BBBP and ranking second on both ClinTox and SIDER. On Tox21, DGT performs comparably to the leading methods and is only marginally outperformed by Pretrained GNN, UniMol, and MolBERT. The results for the single-label classification tasks are unaffected by this correction.

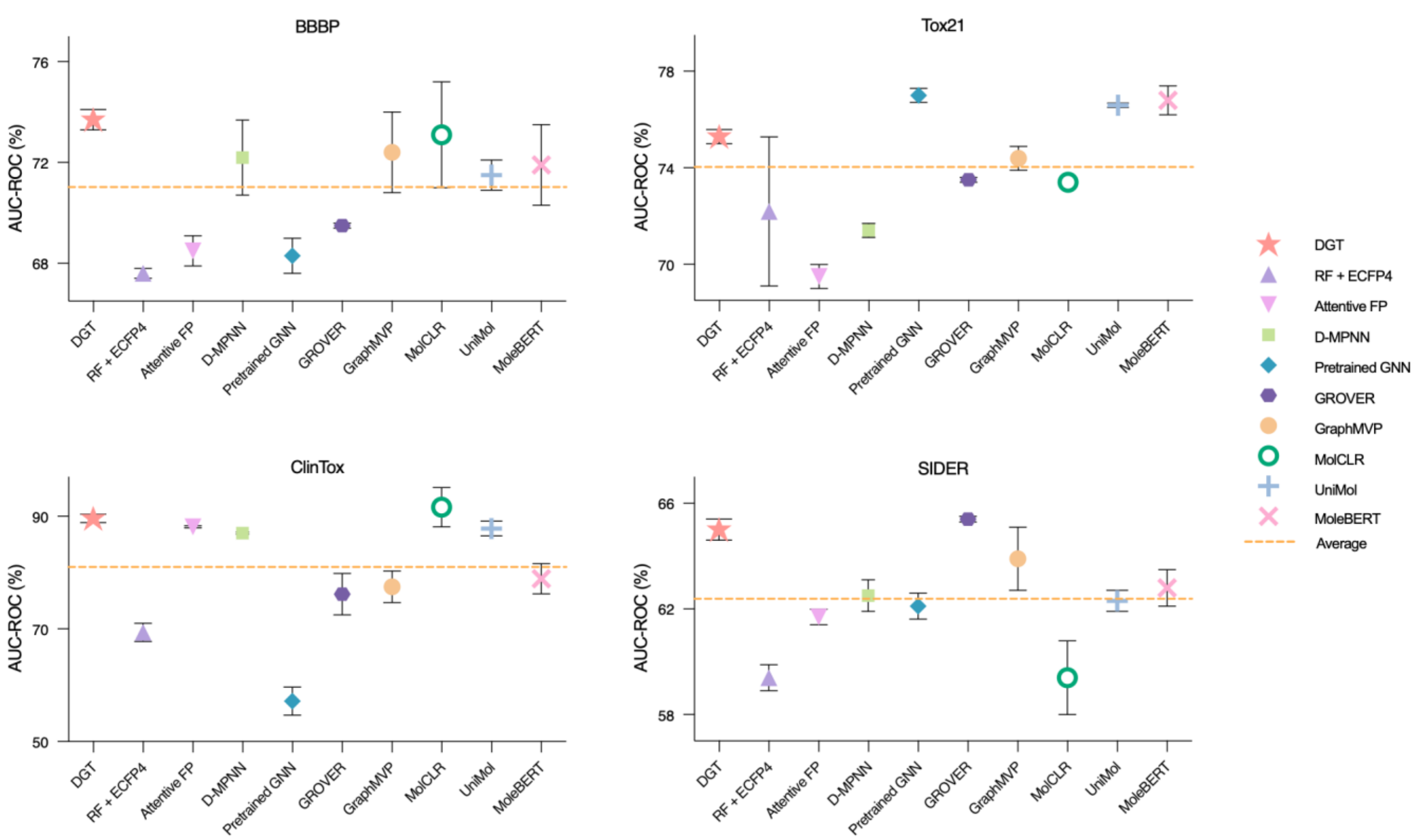

Fig. R2: Reproduced Fig. S4. Detailed Performance comparison of DGT against nine baselines on BBBP, Tox21, ClinTox, and SIDER datasets within the physiology domain. DGT achieves the best performance

on BBBP and ranks second on ClinTox and SIDER. On Tox21, DGT remains highly competitive, with performance slightly below that of Pretrained GNN, UniMol, and MolBERT.

Fig. 2 has been revised accordingly in the manuscript. Overall, DGT still achieves the best average performance in the physiology domain, comprising the BBBP, Tox21, ClinTox, and SIDER datasets. These updated results demonstrate that the main conclusions of the study remain unchanged.

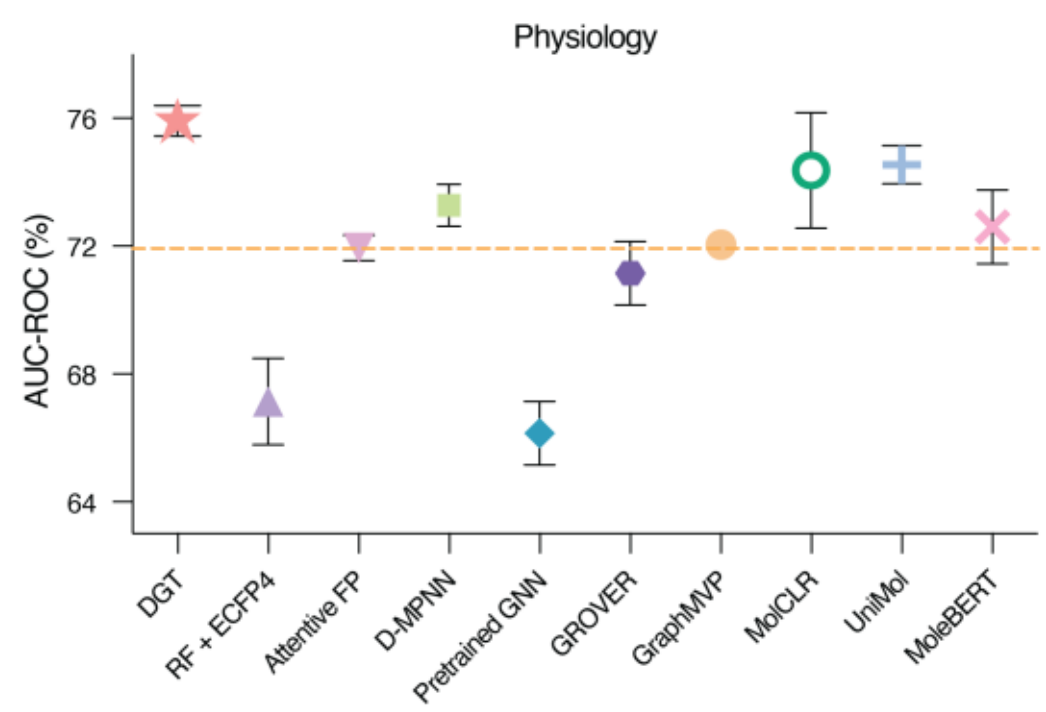

Fig. R3: Reproduced DGT benchmark results in the physiology domain.

All relevant text and numerical results have been updated accordingly in the manuscript. We have also clarified and explicitly emphasised the use of the macro-average AUC-ROC metric in the Methods Section to improve the clarity of the evaluation protocol.

> **Evaluation metrics.** We benchmarked DGT across 58 molecular property prediction tasks spanning four distinct domains, each evaluated using domain-appropriate metrics. The AUC-ROC was utilised for classification tasks within the physiology and biophysics domains, as it quantifies a model's ability to distinguish between classes across various threshold settings; a higher AUC-ROC value indicates better classification performance. Notably, for multi-label classification tasks, the macro-average AUC-ROC is adopted, where the AUC is first calculated for each class separately and subsequently averaged across all classes, thereby providing a balanced evaluation of model performance across heterogeneous labels.

Reviewer 3

RC: ***I appreciate the authors' careful and comprehensive responses to my previous comments. The revised manuscript has adequately addressed my concerns, and the additional analyses and textual revisions have strengthened the study. I have no further major comments and support publication of the manuscript.***

AR: We are grateful for the reviewer's acknowledgement that the previous concerns have been adequately addressed and appreciate the recommendation for publication.

References

1. Adams, K., Pattanaik, L. & Coley, C. W. Learning 3D Representations of Molecular Chirality with Invariance to Bond Rotations. Preprint at https://doi.org/10.48550/arXiv.2110.04383 (2021).
2. Fawcett, T. An introduction to ROC analysis. *Pattern Recognition Letters* **27**, 861–874 (2006).
